# Trunk postural control during unstable sitting among individuals with and without low back pain: A systematic review with an individual participant data meta-analysis

**DOI:** 10.1371/journal.pone.0296968

**Published:** 2024-01-24

**Authors:** Mansour Abdullah Alshehri, Hosam Alzahrani, Wolbert van den Hoorn, David M. Klyne, Albert H. Vette, Brad D. Hendershot, Brad W. R. Roberts, Christian Larivière, David Barbado, Francisco J. Vera-Garcia, Jaap H. van Dieen, Jacek Cholewicki, Maury A. Nussbaum, Michael L. Madigan, Norman Peter Reeves, Sheri P. Silfies, Stephen H. M. Brown, Paul W. Hodges

**Affiliations:** 1 NHMRC Centre of Clinical Research Excellence in Spinal Pain, Injury & Health, School of Health and Rehabilitation Sciences, The University of Queensland, Brisbane, Australia; 2 Physiotherapy Department, Faculty of Applied Medical Sciences, Umm Al-Qura University, Mecca, Saudi Arabia; 3 Department of Physical Therapy, College of Applied Medical Sciences, Taif University, Taif, Saudi Arabia; 4 Department of Mechanical Engineering, Donadeo Innovation Centre for Engineering, University of Alberta, Edmonton, Canada; 5 Glenrose Rehabilitation Hospital, Alberta Health Services, Edmonton, Canada; 6 Extremity Trauma and Amputation Center of Excellence, Defense Health Agency, Falls Church, Virginia, United States of America; 7 Institut de recherche Robert-Sauvé en santé et en sécurité du travail (IRSST), Montreal, Quebec, Canada; 8 Center for Interdisciplinary Research in Rehabilitation of Greater Montreal (CRIR), Montreal Rehabilitation Institute, Montreal, Quebec, Canada; 9 Sport Research Centre, Department of Sport Sciences, Miguel Hernández University of Elche, Alicante, Spain; 10 Institute for Health and Biomedical Research (ISABIAL Foundation), Miguel Hernández University of Elche, Alicante, Spain; 11 Department of Human Movement Sciences, Faculty of Behavioural and Movement Sciences, Vrije Universiteit Amsterdam, Amsterdam Movement Sciences, Amsterdam, Netherlands; 12 Center for Neuromusculoskeletal Clinical Research, Michigan State University, Lansing, Michigan, United States of America; 13 Department of Osteopathic Manipulative Medicine, Michigan State University, East Lansing, Michigan, United States of America; 14 Virginia Tech-Wake Forest School of Biomedical Engineering and Sciences, Virginia Tech, Blacksburg, Virginia, United States of America; 15 Department of Industrial and Systems Engineering, Virginia Tech, Blacksburg, Virginia, United States of America; 16 Sumaq Life LLC, East Lansing, Michigan, United States of America; 17 Department of Exercise Science, University of South Carolina, Columbia, South Carolina, United States of America; 18 Department of Human Health and Nutritional Sciences, University of Guelph, Guelph, Ontario, Canada; University of Illinois at Urbana-Champaign, UNITED STATES

## Abstract

**Introduction:**

Sitting on an unstable surface is a common paradigm to investigate trunk postural control among individuals with low back pain (LBP), by minimizing the influence lower extremities on balance control. Outcomes of many small studies are inconsistent (e.g., some find differences between groups while others do not), potentially due to confounding factors such as age, sex, body mass index [BMI], or clinical presentations. We conducted a systematic review with an individual participant data (IPD) meta-analysis to investigate whether trunk postural control differs between those with and without LBP, and whether the difference between groups is impacted by vision and potential confounding factors.

**Methods:**

We completed this review according to PRISMA-IPD guidelines. The literature was screened (up to 7^th^ September 2023) from five electronic databases: MEDLINE, CINAHL, Embase, Scopus, and Web of Science Core Collection. Outcome measures were extracted that describe unstable seat movements, specifically centre of pressure or seat angle. Our main analyses included: 1) a two-stage IPD meta-analysis to assess the difference between groups and their interaction with age, sex, BMI, and vision on trunk postural control; 2) and a two-stage IPD meta-regression to determine the effects of LBP clinical features (pain intensity, disability, pain catastrophizing, and fear-avoidance beliefs) on trunk postural control.

**Results:**

Forty studies (1,821 participants) were included for the descriptive analysis and 24 studies (1,050 participants) were included for the IPD analysis. IPD meta-analyses revealed three main findings: (a) trunk postural control was worse (higher root mean square displacement [RMS_displ_], range, and long-term diffusion; lower mean power frequency) among individuals with than without LBP; (b) trunk postural control deteriorated more (higher RMS_displ_, short- and long-term diffusion) among individuals with than without LBP when vision was removed; and (c) older age and higher BMI had greater adverse impacts on trunk postural control (higher short-term diffusion; longer time and distance coordinates of the critical point) among individuals with than without LBP. IPD meta-regressions indicated no associations between the limited LBP clinical features that could be considered and trunk postural control.

**Conclusion:**

Trunk postural control appears to be inferior among individuals with LBP, which was indicated by increased seat movements and some evidence of trunk stiffening. These findings are likely explained by delayed or less accurate corrective responses.

**Systematic review registration:**

This review has been registered in PROSPERO (registration number: CRD42021124658).

## 1. Introduction

Low back pain (LBP) is a multifactorial condition [1] and the leading cause of disability globally [2–4]. Recurrence of LBP episodes is common [1, 5, 6], and in some cases LBP becomes chronic [7, 8]. The quality of trunk postural control has been suggested as a risk factor for LBP development, recurrence, and/or perpetuation, mediated by effects of suboptimal loading on spine tissue health [9–11]. Although this proposal is plausible, LBP is heterogeneous and findings from many (small samples) studies are inconsistent and inconclusive.

Trunk postural control is critical for executing human motion and completing everyday activities [12]. Such control can be reflected in the capacity to maintain both postural equilibrium (control of the centre of mass over the base of support) and postural orientation between segments (within spine regions and between the spine and other body regions) [13–15]. Trunk postural control requires motor skill [16], involving the integration of kinematic (position and movement) feedback from visual, vestibular, and proprioceptive systems [17, 18], and the generation of coordinated motor output using an array of muscles [12, 19, 20].

**Fig 1:**
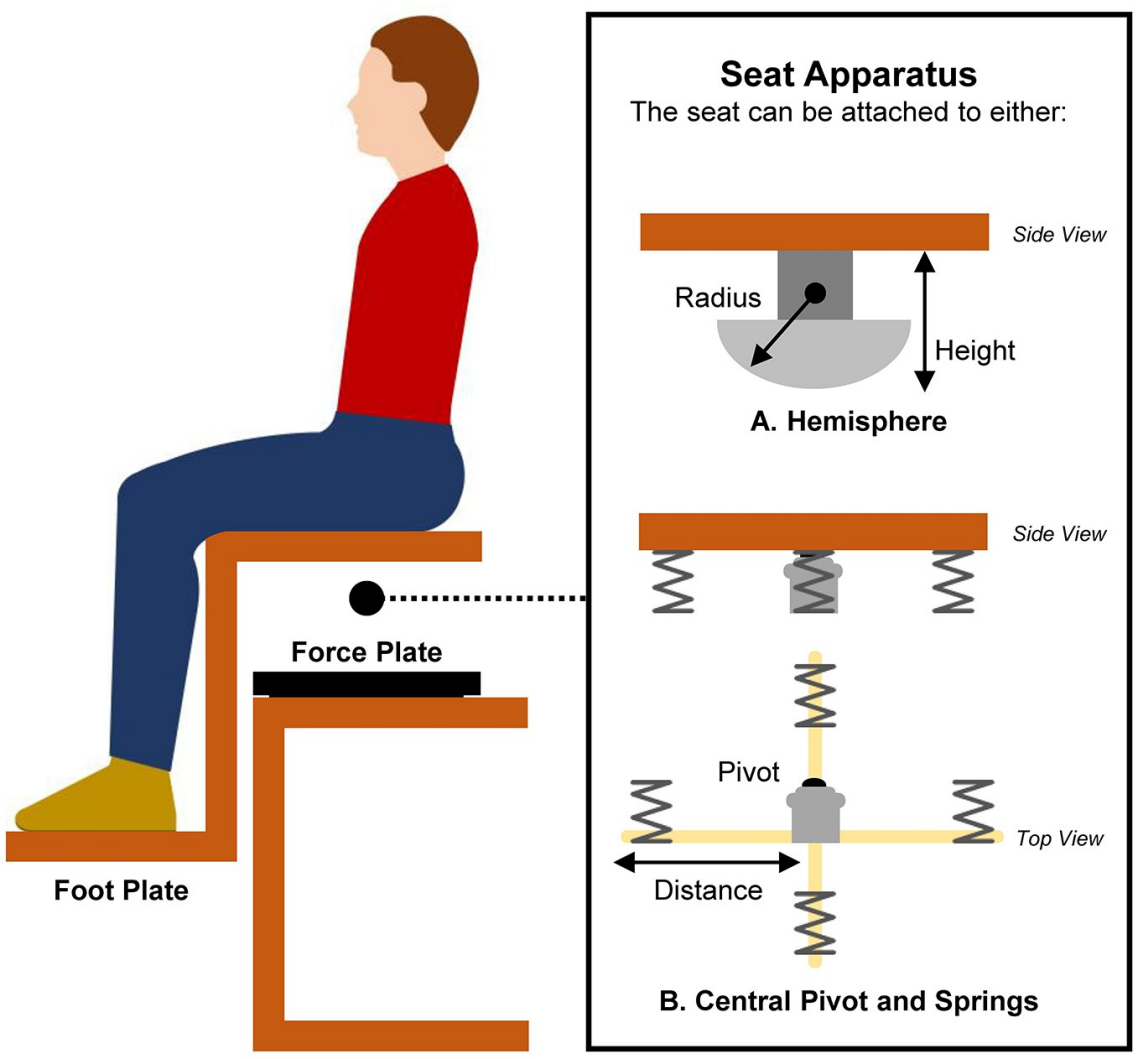
Illustration of the unstable sitting paradigm. A seat is attached to (A) a hemisphere or (B) a central pivot surrounded by four springs. Task difficulty depends on the seat apparatus and its build characteristics. For the hemisphere-based seat, task difficulty depends on the radius of a hemisphere and the seat height from the support surface. For the springs-based seat, task difficulty depends on the stiffness of springs and the distance of springs (R_spring_) from the pivot in percentage. The percentage of R_spring_ is relative to the gravitation gradient (e.g., 100% = the location of springs that would fully balance the mass of the participant as if sitting on a stable chair). Both seats are commonly positioned on a force platform. A foot plate is usually attached to the seat that maintains the knees at 90° flexion and reduces the contribution of the lower limbs to balance control. In most applications, participants are asked to keep their arms crossed at their chest, to minimize the contribution of the upper limbs.

An unstable sitting paradigm has been developed to assess the contribution of the trunk to postural control [21], by limiting contributions from the legs and arms [22]. This paradigm (Fig 1) involves sitting on an unstable surface attached to a hemisphere [21] or on a chair (aka “wobble chair”) that moves about a central pivot and is supported by four adjustable springs [23]. Typically, the seat is placed over a force platform, and the seat movement is measured by calculating time series of the centre of pressure (CoP)–the location of the point of contact of the seat hemisphere [21, 22], or the barycentre of the forces under the wobble chair with springs [24]. To maintain balance in this paradigm, the global position of the upper body is maintained via dynamic movements at the base/seat [25]. Movements at the base/seat are attenuated by coordinated movements of the hip and spine [26, 27] to limit upper body movements, and maintain the overall centre of mass close to the CoP.

Many outcome measures from this paradigm have been reported to have acceptable to excellent test-retest reliability for assessing trunk postural control among individuals with or without LBP [21, 24, 28, 29]. These outcome measures are usually related to the amplitude of CoP/seat motion, and include root mean square (RMS) displacement, mean velocity, range, mean frequency, and other measures related to CoP/seat dispersion dynamics (stabilogram diffusion analysis). In most cases, greater CoP/seat motion is interpreted as inferior or impaired trunk postural control [19, 22, 30].

Many individuals with and without LBP have been studied using the unstable sitting paradigm. Results from some studies suggest that trunk postural control is inferior among those with LBP [19, 22, 30–32], whereas other results indicate no difference from those without LBP [29, 33, 34]. These conflicting findings might be explained by differences in LBP presentation (e.g., LBP clinical features), experimental setup (e.g., seat apparatus and its build characteristics) and protocol (e.g., visual condition), or statistical approaches. Regarding the latter, there is variability in whether covariates/confounders were included, such as age, sex, and body mass index (BMI) [21, 34, 35]. Progress in understanding trunk postural control in LBP would be aided by a systematic review of available studies and an individual participant data (IPD) meta-analysis.

An IPD meta-analysis enables calculating more precise estimates of effects (due to increased statistical power) [36–40], inclusion of explanatory covariates [36–38], adjustment for confounding factors [38–40], more powerful investigation of interactions [36–38, 40], and exploring between-study heterogeneity [37, 38, 40]. Although prior systematic reviews have considered postural control among individuals with and/or without LBP during static/perturbed sitting [41–44], semi-sitting [41–43], standing [41–54], or walking [55], trunk postural control has not been evaluated in unstable sitting, and no reviews have used an IPD meta-analysis.

In the current study, we performed a systematic review with an IPD meta-analysis (when data were available) and a narrative review (when IPD were not available) of studies that used an unstable sitting paradigm to investigate trunk postural control among individuals with LBP and/or those without LBP (pain-free controls/individuals). Our aims were to:

1. Identify characteristics and summarize the main findings of studies that investigated trunk postural control among individuals with and/or without LBP when seated on an unstable surface.
2. Summarise the experimental methods used.
3. Assess the comprehensiveness of reporting and methodological quality.
4. Use IPD meta-analysis to determine whether trunk postural control (primary outcome measures included root mean square displacement [RMS_displ_] and mean velocity [M_vel_]) differs between individuals with and without LBP, by using studies that included both individuals with and without LBP.
5. Use IPD meta-analysis to determine whether similar conclusions are derived from alternative (secondary) outcome measures (e.g., stabilogram diffusion analysis) that are available for fewer studies, by using studies that included both individuals with and without LBP.
6. Use IPD meta-analysis to investigate the interaction between participant‐level variables (age, sex, and BMI) and the difference between groups on trunk postural control, by using studies that included both individuals with and without LBP.
7. Use IPD meta-analysis to determine whether trunk postural control differs between individuals with and without LBP when vision is removed, by using studies that included both individuals with and without LBP in conditions with eyes open and closed.
8. Use IPD meta-regression to identify whether trunk postural control among individuals with LBP depends on pain intensity, pain duration, disability, and psychological features, by using all studies that included individuals with LBP.
9. Use visual inspection of mean plots to compare the outcomes of studies that tested both individuals with and without LBP (IPD meta-analysis) with: (a) the outcomes of studies that tested only LBP or pain-free individuals using IPD analysis (standardised statistical methods); and (b) a narrative review of studies with outcomes for which IPD could not be obtained.

## 2. Methods

### 2.1. Design, registration, and ethics

This systematic review with IPD meta-analyses was planned according to the Preferred Reporting Items for Systematic Review and Meta-Analyses of Individual Participant Data (PRISMA-IPD) guidelines [56]. The study was registered in PROSPERO (registration number: CRD42021124658), and the study protocol has been published [57]. Ethics exemption for this systematic review with IPD analysis was obtained from the Institutional Human Research Ethics Committee (The University of Queensland: 2019003026). Other ethical considerations (e.g., ownership and confidentiality of the IPD set of included studies) have been described previously [57].

### 2.2. Eligibility criteria

#### 2.2.1. Inclusion criteria

Studies were included if they investigated trunk postural control using an unstable sitting paradigm among participants aged ≥18 years. Cross-sectional studies, clinical trials, and cohort studies with baseline data were considered. Studies had to include any of the following: (1) individuals with and without LBP (LBP versus pain-free control); (2) individuals with only LBP; or (3) only pain-free individuals. Participants with LBP of any duration were considered (e.g., acute, subacute, or chronic). Participants had to perform trunk postural control tasks using an unstable sitting paradigm that required individuals to control balance in the sagittal and frontal plane. Trunk postural control must have been measured by quantifying seat motion, either from CoP (force platform) or seat angle (from motion capture systems/sensors).

#### 2.2.2. Exclusion criteria

Studies with insufficient details to determine eligibility were excluded if the authors did not respond to requests (at least two attempts via email) to provide the required information. Non-English reports were excluded. For individuals with only LBP, studies were excluded if they included participants with: neurological disorders (e.g., stroke) except for sciatica (pain due to sciatic nerve compression); spinal structure deformities (e.g., scoliosis); cancer or infection; spine surgery; or a major injury/pain in any other body region within the preceding 12 months. For pain-free controls/individuals, studies were excluded if they included participants with: a history of LBP in the previous year; neurological disorders; structure deformities; cancer or infection; spine surgery; a major injury/pain in any body region within the preceding 12 months. In addition, studies were excluded if they: investigated disorders or diseases other than LBP; involved different seated balance tasks, such as provision of visual feedback or moving the seat to specific target locations; involved sitting on a hemisphere/springs but with feet supported on the floor; involved sitting tasks with perturbations; involved sitting on soft surfaces (e.g., a ball, foam, or air cushion); or studies that referred to an already identified dataset (e.g., studies that referred to secondary use of the same data presented in another study), these studies were excluded from the quantitative analysis but included in the descriptive analysis.

#### 2.2.3. Outcome measures

Table 1 presents additional information about the outcome measures. The primary outcome measures were RMS_displ_ and M_vel_ of the CoP/seat angle during trials in which participants balanced with eyes open or closed in the forward-to-backward (anteroposterior) and side-to-side (mediolateral) directions. Greater RMS_displ_ and M_vel_ are generally considered to reflect impaired postural control of the trunk [22, 30]. These measures are those most often used to quantify postural control. RMS_displ_ [21, 24] and M_vel_ [21, 24, 28] measures have high test-retest reliability during unstable sitting tasks. These measures of postural control have also successfully differentiated individuals with and without pathologies [58]. Secondary outcome measures included those less commonly reported/extracted, including range, mean power frequency (MPF), and measures related to stabilogram diffusion analysis [21, 24].

**Table 1:** Primary and secondary outcome measures.

Outcome		Unit		Description
		CoP	Angle*	
**Primary**	RMS_displ_	mm	degree (°)	Root mean square (RMS) of CoP (or seat angle) displacement time series after subtracting the mean position
	M_vel_	mm/s	°/s	Total path length travelled by CoP (or seat angle) divided by total trial duration
**Secondary**	Range	mm	°	Distance between minimum and maximum CoP (or seat angle) positions
	MPF	Hz	Hz	Mean power frequency (MPF) of CoP (or seat angle)
	D	mm^2^/s	°^2^/s	Diffusion coefficient (D) that reflects how fast (slope) CoP (or seat angle) is diffusing (spreading). Sometimes referred to as the energy/stochastic activity of CoP (or seat angle)
	D_short_	mm^2^/s	°^2^/s	Linear slope fitted to the early part of the diffusion-time profile (short-term diffusion coefficient)
	D_long_	mm^2^/s	°^2^/s	Linear slope fitted to the later part of the diffusion-time profile (long-term diffusion coefficient)
	CP			Critical point (CP) reflecting the intersection coordinates (time and distance) of the short and long-term slopes
	CP_time_	s	s	Mean time coordinate of the critical point
	CP_dist_	mm^2^	°^2^	Mean squared distance coordinate of the critical point

**Abbreviations:** CoP, centre of pressure; M_vel_, mean velocity.

*Some studies calculated seat angle (as a surrogate of CoP) from motion capture systems/sensors to assess trunk postural control.

### 2.3. Identifying studies

The following electronic databases were searched from their date of establishment to 25 March 2022 (the original search): MEDLINE and CINAHL via EBSCO, Embase and Scopus via Elsevier, and Web of Science Core Collection via Clarivate. An updated search was performed on the 7^th^ September 2023 to retrieve new records only (from March 2022 to September 2023). Reference lists of included studies were screened for other relevant studies. Corresponding authors of included studies were contacted and asked if they had other studies on the same topic. Search terms were determined based on the inclusion criteria (see S1–S8 Tables for details on our original and updated search strategies).

### 2.4. Study selection

EndNote software (version X9) was used to collect the search results, and to remove duplicates automatically and manually. Titles and abstracts of articles were screened for potential inclusion by two independent reviewers (MAA and HA) familiar with systematic reviews and meta-analyses. For articles that potentially met eligibility criteria, full texts were reviewed for final decisions. Disagreement between reviewers was resolved by consensus or a third reviewer (PWH). Cohen’s kappa (inter-rater reliability) analysis [59] was performed to assess the degree of agreement between reviewers. The number of included and excluded articles, and reasons for exclusion, were recorded.

### 2.5. IPD collection

The corresponding authors of included studies were asked to share their IPD using author information reported in the article, or profiles on their university websites. If no response was received, co-authors were contacted. Authors were informed about the proposal IPD meta-analysis study/methods, and were asked if they were willing to provide their IPD sets. Any eligible studies for which IPD could not be obtained (e.g., authors did not respond or did not have access/authorization to provide IPD set) were retained for narrative analysis.

### 2.6. Data items

Study- and individual-level data were extracted using a standardised form. Extracted data were [57]: study characteristics, participant characteristics, LBP clinical features, inclusion and exclusion criteria, experimental setup, experimental protocol, any reported adverse effects, outcome measures, and main findings. Collected IPD sets were stored in a master spreadsheet and were screened in terms of presentation of overall data and available variables. The unit of measurement for each outcome measure was unified. For instance, some outcome measures from CoP data, such as RMS_displ_, were reported in two different units (cm or mm) and the unit of RMS_displ_ was unified as “mm” before applying the IPD meta-analysis.

### 2.7. Comprehensiveness of reporting and methodological quality

Comprehensiveness of reporting and quality of methods were assessed with an adapted checklist [57], which includes components from a quality checklist developed by Mazaheri et al. [46] and Ruhe et al. [45] for systematic reviews of balance measures. This checklist has 25 items with three options to assess the comprehensiveness of reporting and quality of methods across five main domains: participant characteristics, LBP characteristics, experimental setup/protocol, confounding effects control, and statistical information (S9 Table presents the checklist content and item descriptions). Items were scored as ‘1’ (yes), ‘0.5’ (partially; some information was provided) or ‘0’ (no) by two independent reviewers (MAA and HA). The overall reporting/quality score was obtained from the sum of all scores converted to a percentage. Separate reporting/quality scores were calculated for each domain. Reporting/Quality scores ranged from 0 to 100%, with higher scores indicating higher reporting/quality. Discrepancies between reviewers were settled by consensus and a third reviewer (PWH) when necessary.

### 2.8. Synthesis methods

#### 2.8.1. Descriptive analysis

A descriptive analysis (see S10 Table for a detailed listing of items included for the descriptive analysis) was used to identify characteristics and to summarize main findings of studies that investigated trunk postural control among individuals with and/or without LBP when seated on an unstable surface (**Aim 1**), and to assess the comprehensiveness of reporting and quality of methods (**Aim 3**). All studies were included in the descriptive analysis, including those that reported data from only LBP or pain-free individuals, and studies for which IPD sets were unavailable. Outcomes of each specific study were summarised in tabular format (**Aim 1**) and were discussed narratively only for studies that did not provide IPD (**Aim 9**). A detailed descriptive analysis was also undertaken to describe the experimental setup and protocol (**Aim 2**). Comprehensiveness of reporting and quality of methods before and after obtaining the IPD were summarised in tabular format (**Aim 3**).

#### 2.8.2. IPD meta-analysis

Stata/IC 16.1 software (Release 16, StataCorp LLC, College Station, Texas, USA) was used for statistical analyses and generating forest plots. Stata packages/commands such as ipdmetan, meta esize, meta summarize, meta forestplot, meta funnelplot, meta bias, metareg, and xtmixed were used as appropriate. Results were regarded as statistically significant if *P<*0.05.

A quantitative analysis was conducted using a two-stage IPD meta-analysis to investigate trunk postural control among individuals with and without LBP when sitting on an unstable surface, while considering the characteristics of each individual participant (**Aims 4–5**). The two-stage IPD meta-analysis was obtained by (1) analysing IPD from each study separately to calculate aggregate data of interest using multilevel mixed-effects models, then (2) combining the results using conventional meta-analysis methods. The advantage of this approach is that it applies a standardised statistical method [38–40] and enables to control for covariates and confounding factors in the analysis [36–40]. Potential confounding variables of age and BMI (as covariates) and sex (as a fixed factor) [21, 34, 35] were included in the models. This analysis was limited to studies that included both individuals with and without LBP to identify between-group differences and was performed for each identified outcome (RMS_displ_, M_vel_, range, MPF, D_short_, D_long_, CP_time_ and CP_dist_; for definitions see Table 1), visual condition (eyes open and closed), and direction (anteroposterior and mediolateral directions).

A random effects model, fitted using the restricted maximum likelihood (REML) method, was used [60] to avoid misleading effect estimates and potentially inappropriate conclusions [61]. Overall and individual study (observed) effect sizes were estimated using standardised mean differences (SMDs, Hedge’s g), since different studies used different units to measure the same outcome [62]. SMD effect sizes can be classified as trivial (>0.2), small (0.2–0.5), medium (0.5–0.8) or large (>0.8) [63]. The I^2^ index [64] was calculated to assess the percentage of total variability due to between-study heterogeneity rather than sampling error (within-study variability) [65, 66]. Prediction interval (PI) can provide a predicted range for the true effect (without sampling error) in a new (similar) study [67–69]. This was calculated (when having a sufficient number of studies) to estimate how much the effect size varies across studies included in the IPD meta-analysis [70, 71]. The potential presence of small-study effects (also known funnel plot asymmetry or publication bias) in the IPD meta-analysis was tested using Egger’s test (random-effects model with the REML method) and visualized using funnel plots.

A two‐stage IPD analysis was performed to investigate the interaction between participant‐level variables (age, sex, and BMI) and the difference between groups (**Aim 6**). In the first stage, group and participant‐level variables and their interactions were entered using multilevel mixed-effects models. An interaction of one variable (age) with group was entered and the remaining variables (sex and BMI) were included without interaction terms in the models. This method was applied for each of the participant‐level variables, and was performed for each study separately. In the second stage, a conventional meta-analysis was performed to pool interaction effect (adjusted) coefficients using a random-effects model with the REML method. Both I^2^ index and PI were calculated. This analysis was limited to studies that included both individuals with and without LBP, and was performed for each identified outcome, visual condition, and direction.

A two‐stage IPD analysis was performed to investigate the interaction between vision (eyes open and closed) and the difference between groups (**Aim 7**). In the first stage, group and visual condition and their interaction were entered using multilevel mixed-effects models (for each study separately), including age and BMI as covariates, and sex as a fixed factor in the models. In the second stage, a conventional meta-analysis was performed to pool interaction effect (adjusted) coefficients using a random-effects model with the REML method. Both I^2^ index and PI were calculated. This analysis was limited to studies that included both individuals with and without LBP and that investigated the effect of vision on trunk postural control, and was performed for each identified outcome and direction.

A two‐stage IPD meta-regression was performed on data from studies that included individuals with LBP to identify the relationship between LBP clinical features and trunk postural control (**Aim 8**). In the first stage, each LBP clinical feature (pain intensity, disability, pain catastrophizing, and fear-avoidance beliefs) was entered using multilevel mixed-effects models (for each study separately), including participant‐level variables (age, sex, and BMI). In the second stage, a standard meta-regression was performed to pool regression (adjusted) coefficients using a random-effects model with the REML method. This analysis was performed for each identified outcome, visual condition, and direction.

#### 2.8.3. Other analysis

Descriptive statistics (e.g., means and standard errors) of the outcomes from studies with data from only LBP or pain-free individuals were plotted with the outcomes from other studies with both groups (individuals with and without LBP) using IPD (if available) or aggregate data (**Aim 9**). We did this by applying standardised statistical methods (multilevel mixed-effects models), including confounding variables (age, sex, and BMI). This analysis was performed for each identified outcome, visual condition, and direction. We compared between groups using the visual inspection of mean plots. Outcomes from studies for which IPD could not be obtained were summarised briefly in a narrative manner and were contrasted with the findings of the IPD meta-analysis.

## 3. Results

### 3.1. Study selection

**Fig 2:**
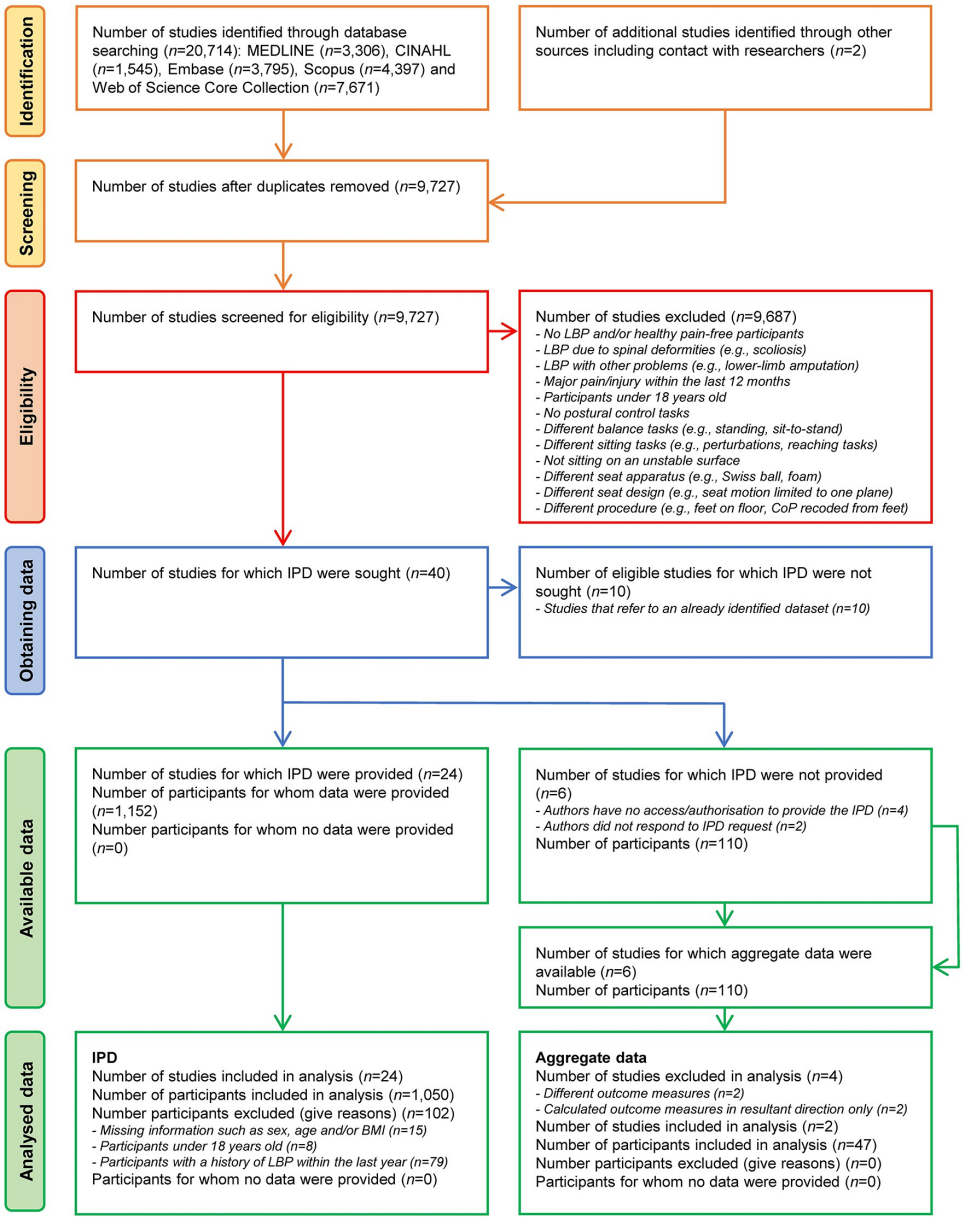
PRISMA-IPD flow diagram of the study selection process. LBP = low back pain; IPD = individual participant data; BMI = body mass index.

From among 18,595 identified articles, a total of 40 articles [19, 21–35, 72–95] met the eligibility criteria and was included for the descriptive analysis. After removal of studies that referred to secondary use of data presented in another study (*n* = 10), and studies for which individual-level data could not be provided (*n* = 6), IPD sets were obtained from 24 articles for the IPD analysis [19, 22, 23, 25, 28–35, 73, 77, 78, 80, 83–85, 87, 88, 90–92]. Fig 2 shows the PRISMA-IPD flow diagram. There was a moderate agreement (κ = 0.45) between both reviewers on study inclusion and exclusion decisions, and all disagreements were resolved by consensus.

### 3.2. Descriptive analysis

#### 3.2.1. Characteristics of participants

Forty studies with a total of 1,821 participants were included in this systematic review. Fourteen studies (1,236 participants) involved comparisons between individuals with (636 participants) and without (600 participants) LBP [19, 22, 24, 29–35, 72, 74–76], one study (18 participants) included individuals with only LBP [73] and the remaining 25 studies (567 participants) included pain-free individuals [21, 23, 25–28, 77–95] either alone or with comparison to individuals with conditions other than LBP. Sex, age, and BMI characteristics are described in Tables 2 and 3. S11 and S12 Tables show the inclusion and exclusion criteria used in the included studies.

**Table 2:** Participant characteristics for studies with data from individuals with LBP.

Study	Group *LBP stage (pain duration)* *Control*	Sample Size [IPD analysis]*			Mean ± Standard Deviation			
		Total	Male	Female	Age (years)	Height (cm)	Weight (kg)	BMI (kg/m^2^)
Radebold et al. [22]	*Chronic LBP (> 6 months)*	16 [16]	15 [15]	1 [1]	38.8±10.1	175.8±8.6	81.9±15.3	26.3±3.1
	*Control*	14 [14]	13 [13]	1 [1]	38.1±9.6	176.6±8.9	80.4±17.5	25.6±4.2
Reeves et al. [73]	*Chronic LBP (> 6 months)*	18 [18]	10 [10]	8 [8]	38.6±12.6	174.2±10	75.7±12.4	24.9±3
Navalgund [72]†	*Subacute LBP (≤ 8 weeks)*	10	5	5	32.6±11.1	174±13	84.2±19.9	27.3±4.5
	*Control*	10	5	5	35±8.3	175±9	79±22.3	26.1±7.1
van Dieën et al. [33]	*Current LBP (not available)*	58 [58]	21 [21]	37 [37]	42.1±0.7	176.3±8.5	77.7±14.2	24.9±3.8
	*Recent LBP (not available)* §	79	36	43	42.2±0.7	177.2±9	75.9±12.1	24.1±3.1
	*Control*	164 [160]	83 [82]	81 [78]	42±0.7	176.8±8.7	78.1±14.7	24.9±3.8
van Dieën et al. [74]‡	*Current LBP (not available)*	58	21	37	42.1±0.7	176.3±8.5	77.7±14.2	24.9±3.8
	*Recent LBP (not available)* §	79	36	43	42.2±0.7	177.2±9	75.9±12.1	24.1±3.1
	*Control*	164	82	78	42±0.7	176.8±8.7	78.1±14.7	24.9±3.8
Willigenburg et al. [31]	*Subacute to chronic LBP (≥ 6 weeks)*	20 [20]	11 [11]	9 [9]	33.4±15.6	178.7±11.2	76±15.5	23.6±3
	*Control*	11 [11]	7 [7]	4 [4]	32.6±10.4	178±12.2	71.3±9	22.5±2.5
Larivière et al. [24]‡	*Chronic LBP (≥ 3 months)*	17	9	8	38±13	169.6±10.5	70±12.7	24.2±2.5
	*Control*	19	9	10	38.7±14	170±7.1	67.4±12.8	23.1±2.7
Larivière et al. [34]	*Chronic LBP (≥ 3 months)*	17 [17]	9 [9]	8 [8]	38±13	169.6±10.5	70±12.7	24.2±2.5
	*Control*	19 [19]	9 [9]	10 [10]	38.7±14	170±7.1	67.4±12.8	23.1±2.7
Sung et al. [19]	*Acute to subacute LBP (< 3 months)*	33 [34]	13 [13]	20 [21]	33.6±14.9	170±8.5	75.5±16.4	26±4.9
	*Control*	33 [34]	13 [13]	20 [21]	35.4±13.8	169.4±9.4	68.4±11.9	23.8±3.3
Shahvarpour et al. [75]‡	*Chronic LBP (≥ 3 months)*	6	6	NA	-	179±7	82.8±18.9	-
	*Control*	6	6	NA	-	178±9	81.2±29	-
Shahvarpour et al. [29]	*Acute to chronic LBP (≥ 4 weeks)*	34 [35]	15 [16]	19 [19]	46.1±12.6	167.2±7.4	73.5±11.4	26.3±3.4
	*Control*	30 [30]	15 [15]	15 [15]	39.6±14	171.1±10	70±12.7	23.8±3.4
Shahvarpour et al. [32]	*Acute to chronic LBP (≥ 4 weeks)*	40 [40]	20 [20]	20 [20]	42.9±11.2	169.1±9.3	70.6±11.7	24.6±2.9
	*Control*	20 [19]	10 [9]	10 [10]	39.8±13.4	169.5±7.8	69±11.8	23.9±2.7
Cyr et al. [30]	*Chronic LBP (> 3 months)*	10 [10]	2 [2]	8 [8]	40.6±5.3	-	-	25.1±3.1
	*Control*	10 [10]	2 [2]	8 [8]	41.4±6.1	-	-	24.6±3.3
Larivière et al. [76]‡	*Acute to chronic LBP (≥ 4 weeks)*	30	15	15	♂43±14 ♀48±11	♂173±6 ♀164±6	♂76±13 ♀72±10	♂25±4 ♀27±3
	*Control*	28	14	14	♂38±14 ♀41±14	♂178±9 ♀164±6	♂77±11 ♀62±10	♂24±3 ♀23±4
van den Hoorn et al. [35]	*Acute LBP (< 2 weeks)*	129 [131]	62 [63]	67 [68]	28.7±8.1	172.6±9	72.7±14.8	24.3±4
	*Control*	72 [72]	29 [29]	43 [43]	26.6±6.6	169.6±10.5	64.6±13.7	22.3±3.1
[Overall]**	*LBP*	636 [361]	298 [172]	342 [189]	**[36.05±11.86]**	[172.36±9.43]	[74.13±14.37]	**[24.86±3.78]**
	*Control*	600 [369]	295 [180]	297 [189]	**[37.48±10.04]**	[173.45±9.87]	[72.61±14.87]	**[23.99±3.58]**

**Abbreviations/Symbols:** LBP, low back pain; IPD, individual participant data; BMI, body mass index; NA, not available; ♂, male; ♀, female.

**Statistics for IPD analysis:** Overall summary measures for studies that provided IPD sets. Fisher’s exact test was used for categorical data (e.g., sex). Independent t-test was used for normally continuous data (e.g., height, weight, and BMI) and Wilcoxon Rank-Sum test was used for not normally distributed continuous data (e.g., age). Values of statistically significant differences (*P*<0.05) are printed bold.

*Number of participants included in the IPD analysis.

^**†**^IPD were not available as authors did not have access/authorisation to provide the IPD set.

^**‡**^Studies that were only included in the descriptive analysis but excluded from quantitative/IPD analysis as these studies referred to an already identified IPD set.

^**§**^No current pain, but pain within last 12 months.

**Overall summary measures for only studies with IPD and included both individuals with and without LBP.

**Table 3:** Participant characteristics for studies with data from only pain-free individuals.

Study	Sample Size [IPD analysis]*			Mean ± Standard Deviation			
	Total	Male	Female	Age (years)	Height (cm)	Weight (kg)	BMI (kg/m^2^)
Cholewicki et al. [21]†	11	9	2	36.9±10.1	176.7±7.7	84.9±21	27±5.8
Silfies et al. [77]	13 [13]	9 [9]	4 [4]	20.8±0.9	175.5±12.5	77.3±12.8	25±2.5
van der Burg et al. [79]‡	8	4	4	63.1±10	177.1±12.6	79±12	-
Reeves et al. [78]	10 [10]	6 [6]	4 [4]	22.3±4.6	173.8±11.8	68.4±11.8	22.6±3.1
Cholewicki et al. [80]	23 [22]	12 [12]	11 [10]	25.3±7.8	173.4±9.8	67.6±12.2	22.4±2.8
Lee and Granata [81]§	12	9	3	♂28±3.1 ♀25.7±2.5	♂178.8±9.5 ♀165.7±4.2	♂80.4±14.8 ♀60.4±3.4	-
Lee et al. [82]‡	12	7	5	♂25.7±6.9 ♀21.4±1.7	♂178.7±6.9 ♀161.3±8.2	♂79.9±9 ♀59.6±8.3	-
Slota et al. [23]	21 [21]	13 [13]	8 [8]	23±4	170.8±12.2	73.9±14	-
Hendershot & Nussbaum [83]	8 [8]	8 [8]	NA	36.9±13.4	174.4±3.9	80.3±11.4	26.5±4.6
Hendershot et al. [84]	12 [12]	6 [6]	6 [6]	23.9±2.5	173.8±12.9	69.3±9.5	22.9±1.4
Barbado et al. [86]†	25	25	NA	23.5±7.2	174±6.6	74.6±11.2	24.5±2.5
Barbado et al. [85]	78 [69]	78 [69]	NA	24.6±5.5	175.1±6.6	74.7±10.4	24.3±2.5
Beaudette et al. [87]	28 [28]	14 [14]	14 [14]	23.8±2.6	175.8±9.4	73.7±14.7	23.6±3
Ruggiero et al. [88]	24 [24]	NA	24 [24]	Range: 20–24	171±6	64.9±10.1	22.1±2.4
Barbado et al. [28]	23 [23]	23 [23]	NA	25.3±5.5	174.5±5.6	73.2±7.4	24±2.3
Barbado et al. [89]†	22	22	NA	24.6±4.6	174±7	73.6±7.5	-
Glofcheskie & Brown [90]	36 [29]	36 [29]	NA	20.6±1.7	176.7±6.3	70.3±8.7	22.5±2
Acasio et al. [91]	13 [13]	11 [11]	2 [2]	28.9±7.9	177.5±5.7	75.2±11.8	23.8±2.5
Williams et al. [92]	15 [15]	10 [10]	5 [5]	23.9±2.3	172.5±10	73.3±13.8	24.4±2.8
Barbado et al. [93]‡	19	12	7	27.9±7.1	-	83.6±11.6	-
Roberts & Vette [25]	15	15	NA	25.3±5.2	179.6±6.7	75.1±13.1	23.1±2.8
Roberts et al. [27]†	15 [15]	15 [15]	NA	25.3±5.2	179.6±6.7	75.1±13.1	23.1±2.8
Acasio et al. [94]†	13	11	2	28.9±7.9	177.5±5.7	75.2±11.8	23.8±2.5
Alshehri et al. [26]†	72	29	43	26.8±6.5	169.2±10.5	64.3±13.9	22.3±3.1
de Oliveira et al. [95]§	39	6	33	22±3 / 22±4 / 26±5	164±9 / 167±5 / 164±6	62±13 / 65±10 / 67±6	22±4 / 24±3 / 25±2
[Overall]**	567 [302]	390 [225]	177 [77]	[24.48±5.99]	[175.04±8.18]	[72.63±11.54]	[23.61±2.74]

**Abbreviations/Symbols:** IPD, individual participant data; BMI, body mass index; NA, not available; ♂, male; ♀, female.

**Statistics for IPD analysis:** Overall summary measures for studies that provided IPD sets.

*Number of participants included in the IPD analysis.

^**†**^Studies that were only included in the descriptive analysis but excluded from quantitative/IPD analysis as these studies referred to an already identified IPD set.

^**‡**^IPD were not available as authors did not have access/authorisation to provide the IPD set.

^**§**^IPD were not available as authors did not respond to IPD request.

**Overall summary measures for only studies with IPD.

All studies with data from individuals with LBP (studies including both individuals with and without LBP or individuals with only LBP; *n* = 15/40) included participants considered to have non-specific LBP. Six studies included participants with chronic LBP [22, 24, 30, 34, 73, 75], one studied acute LBP [35] and another studied subacute LBP [72]. Five studies included participants with mixed LBP presentation/stages [19, 29, 31, 32, 76], such as acute to sub-acute [19], sub-acute to chronic [31], or acute to chronic [29, 32, 76]. Two studies provided insufficient detail [33, 74].

Of the 24 studies included in the IPD analysis, nine provided IPD sets [19, 22, 29–35] for both individuals with (361 participants) and without LBP (369 participants) for 378 female (individuals with LBP = 189; individuals without LBP = 189) and 352 male (individuals with LBP = 172; individuals without LBP = 180) participants. Those with LBP were younger (individuals with LBP = 36.1±11.9 years; individuals without LBP = 37.5±10 years; *P*<0.05) and had higher BMI (individuals with LBP = 24.9±3.8 kg/m^2^; individuals without LBP = 24.0±3.6 kg/m^2^; *P*<0.05) than those without LBP (Table 2). Other studies (*n* = 14/24) included in the IPD analysis [23, 25, 28, 77, 78, 80, 83–85, 87, 88, 90–92] had data for only pain-free individuals (302 participants: 77 female and 225 male participants). For those individuals, mean age and BMI were 24.5±6.0 years and 23.6±2.7 kg/m^2^ (Table 3), respectively.

Most studies with LBP data provided information about LBP intensity (*n* = 12/15) [19, 22, 24, 29–32, 34, 35, 72, 73, 76] and disability (*n* = 10/15) [19, 22, 24, 29, 30, 32, 34, 35, 73, 76] either as IPD or aggregate data, but less than half (*n* = 7/15) [19, 24, 29, 30, 32, 34, 35] provided information about psychological features. For the IPD, the mean scores of pain intensity (visual analogue or numeric pain rating scale), disability (Roland-Morris questionnaire) and pain catastrophizing were 4.3±2, 7.1±4.8 and 16.1±11.2, respectively. The mean scores of physical activity subscale, work subscale and total scale of fear-avoidance beliefs questionnaire were 14.5±6, 13.1±10.8 and 27.3±13.3, respectively. LBP clinical features are described in Table 4.

**Table 4:** Clinical features of individuals with LBP.

Study	Mean ± Standard Deviation							
	Pain Intensity		Disability		Pain Catastrophizing	Fear-Avoidance		
	VAS (/10)	NPRS (/10)	RMDQ (/10, /24, /28)	ODI (/100)	PCS (/52)	FABQ-PA (/30)	FABQ-W (/66)	FABQ (/96)
Radebold et al. [22]	2.7±2	-	/24: 5.1±4.2	-	-	-	-	-
Reeves et al. [73]	4.4±2.2	-	/10: 2.3±1.6§	-	-	-	-	-
Navalgund [72]*	-	4±1.2	-	-	-	-	-	-
van Dieen et al. [33]	-	-	-	-	-	-	-	-
van Dieen et al. [74]†	-	-	-	-	-	-	-	-
Willigenburg et al. [31]	2.7±1.7	-	-	-	-	-	-	-
Larivière et al. [24]†	2.5±1.9	-	/24: 4.2±3.2	-	17.2±10.2	-	-	-
Larivière et al. [34]	2.5±1.9	-	/24: 4.2±3.2	-	17.2±10.2	-	-	-
Sung et al. [19]	-	4±1.8	-	20.8±11.2	12.2±11.1	13.4±8.9	14.3±13.5	28.9±17.8
Shahvarpour et al. [75]†	-	-	-	-	-	-	-	-
Shahvarpour et al. [29]	-	4.9±1.3	/24: 12.1±3.9	29.3±9.7	23.9±12	16.5±5.8	-	-
Shahvarpour et al. [32]	-	2.6±1.6	/24: 5±3.4	-	20.1±9.9	13.9±5.1	16.6±12	30.5±14.6
Cyr et al. [30]	-	3.7±1.9	/24: 6±2.9	22.4±10.1	-	11.9±2.8	11.8±6.8	22.7±8.9
Larivière et al. [76]†	-	♂4.7±1.4 ♀4.6±1	-	♂28±9 ♀28±9	-	-	-	-
van den Hoorn et al. [35]	-	5±1.9	/28: 6.9±4.7	-	13.7±10.3	14.7±5.5	11.8±9.7	26.3±11.7
IPD Sample Size [Overall]‡	276 [4.34±2.01]		232 [7.13±4.77]	79 [24.77±11.08]	254 [16.13±11.22]	248 [14.53±6.04]	213 [13.10±10.78]	214 [27.33±13.34]

**Abbreviations/Symbols:** LBP, low back pain; VAS, Visual Analogue Scale; NPRS, Numeric Pain Rating Scale; RMDQ, Rolland-Morris Disability Questionnaire; ODI, Oswestry Disability Index; PCS, Pain Catastrophizing Scale; FABQ-PA, Fear-Avoidance Beliefs Questionnaire—Physical Activity; FABQ-W, Fear-Avoidance Beliefs Questionnaire—Work; FABQ, fear-avoidance beliefs questionnaire; IPD, individual participant data; ♂, male; ♀, female.

**Statistics for IPD analysis:** Overall summary measures for studies that provided IPD sets.

*IPD were not available as authors did not have access/authorisation to provide the IPD set.

^**†**^Studies that were only included in the descriptive analysis but excluded from quantitative/IPD analysis as these studies referred to an already identified IPD set.

^**‡**^Overall summary measures for only studies with IPD.

^**§**^This was not included in the overall summary measure of RMDQ.

#### 3.2.2. Experimental setup and protocol

S13 and S14 Tables provide detailed information about the experimental setup and protocol used in all included studies. Most studies (*n* = 25/40) used an unstable seat that attached to part of a hemisphere (LBP data: *n* = 8 [19, 22, 30, 31, 33, 35, 73, 74]; only pain-free data: *n* = 17 [21, 25–28, 77–80, 85–90, 92, 93]). Other studies (*n* = 15/40) used an unstable chair that attached to four springs moving about a pivot (LBP data: *n* = 7 [24, 29, 32, 34, 72, 75, 76]; only pain-free data: *n* = 8 [23, 81–84, 91, 94, 95]). Seat characteristics differed between studies. For the hemisphere-base seat, the radius range was 10–25 cm and the seat height from the support surface range was 6.25–19 cm. For the springs-base seat, the distance of springs from the pivot in percentage ranged from 43.5–100%. Some studies (*n* = 11/40) used multiple levels of seat instability [21, 22, 25, 27, 72, 73, 77, 81, 91, 92, 94]. Most studies (*n* = 34/40) attached a foot plate to the seat to maintain knee flexion at 90° [19, 21–24, 26, 28–35, 72–86, 89, 91, 93–95]. Outcome measures of trunk postural control were calculated from CoP data (force platform) in 27/40 studies [19, 21, 22, 28, 30, 31, 33, 35, 72–74, 77–80, 83–91, 93–95], seat angle data (motion capture systems/sensors) in 8/40 studies [25–27, 29, 32, 76, 82, 92], or both methods in 5/40 studies [23, 24, 34, 75, 81].

Trunk postural control was assessed with both eyes open and closed in 12/40 studies [19, 21, 22, 25–27, 30, 31, 35, 77, 78, 92], eyes closed only in 7/40 studies [24, 29, 32, 34, 73, 75, 76], and eyes open only in 21/40 studies [23, 28, 33, 72, 74, 79–91, 93–95]. Trial duration ranged between 7 and 70 s, but most used 60 s or more (*n* = 23/40) [19, 23, 24, 28, 29, 32, 34, 72, 75–77, 81–87, 89–91, 93, 94]. One to six repetitions were recorded. Though many studies (*n* = 25/40) included clear (specific) instruction to participants (e.g., ‘sit as quietly as possible’ or ‘maintain an upright posture’), not all did so [21, 24, 25, 29, 30, 32, 34, 73, 76–78, 80–82, 95].

#### 3.2.3. Comprehensiveness of reporting and methodological quality

The comprehensiveness of reporting and quality of methods were assessed twice (see S15–S20 Tables for details): first using the study-level data as reported in the published version of all included papers (*n* = 40), and second using the IPD obtained from authors (*n* = 24/40).

Using published versions (*n* = 40), the overall reporting/quality score of all domains was 66.3% for studies with LBP data (*n* = 15/40) and 63.4% for studies with only pain-free data (*n* = 25/40). The lowest and highest reporting/quality domain score for studies with LBP data were obtained for the confounding effects control domain (39.4%) and the experimental setup/protocol domain (76.7%), respectively. For studies with only pain-free data, the lowest and highest reporting/quality domain score were obtained for the confounding effects control domain (18.7%) and participant characteristics (80%), respectively.

When reporting/quality was assessed using the IPD set (*n* = 24), there was a marked improvement in reporting/quality scores. The overall reporting/quality score of all domains increased from 67.5% to 90% for studies with LBP data (*n* = 10/24) and from 63.3% to 88.2% for studies with only pain-free data (*n* = 14/24).

#### 3.2.4. Outcome measures and main findings

Primary outcome measures were calculated in most studies: RMS_displ_ (*n* = 26/40) and M_vel_ (*n* = 26/40). Secondary outcome measures were less frequently calculated/reported: range (*n* = 8/40), MPF (*n* = 10/40), D_short_ (*n* = 11/40), D_long_ (*n* = 7/40), CP_time_ (*n* = 8/40), and CP_dist_ (*n* = 6/40). There was an increase in the number of calculated measures when IPD were obtained. Many outcome measures were calculated in both the anteroposterior and mediolateral directions, but some measures were only calculated in the resultant direction. Outcomes and main findings of each study are presented in Tables 5 and 6. The following sections describe the outcomes and results of the IPD analysis and then compare them with the findings of studies that could not be included in the IPD analysis.

**Table 5:** Outcome measures and main findings for studies with data from individuals with LBP.

Study	Primary measures		Secondary measures						Main findings	IPD
	RMS_displ_	M_vel_	Range	MPF	D_short_	D_long_	CP_time_	CP_dist_		
Radebold et al. [22]	AP, ML	R	AP, ML	-	R	R	R	R	RMS_displ_ (AP-SIL 1–2; ML-SIL 2), M_vel_ (R-SIL 1), range (AP-SIL 1–2; ML-SIL 2), D_short_ (R-SIL 2), D_long_ (R-SIL 2), & CP_dist_ (R-SIL 2) were higher in LBP than control. RMS_displ_, M_vel_, range, D_short_, & D_long_ increased with higher seat instability level (task difficulty) & EC.	✓
Reeves et al. [73]	-	AP, ML	-	-	-	-	-	-	M_vel_ (ML) was lower in the 25–50% stochastic resonance stimulation of paraspinal muscles than the 0% stochastic resonance stimulation.	✓
Navalgund [72]*	R	-	-	-	R	R	-	-	RMS_displ_ (R) & D_short_ (R) were higher in LBP than control during the highest seat instability level. All measures were increased with higher seat instability level.	☓
van Dieen et al. [33]	AP, ML	-	-	AP, ML	AP, ML	-	AP, ML	AP, ML	No differences between current-LBP & no-LBP groups in RMS_displ_ (AP, ML), CP_time_ (AP, ML), & CP_dist_ (AP, ML). MPF (AP) & D_short_ (AP) were lower in current-LBP than no-LBP. RMS_displ_ (AP, ML), D_short_ (AP), & CP_dist_ (AP, ML) were lower in recent-LBP than no-LBP.	✓
van Dieen et al. [74]†	AP, ML	R	AP, ML	AP, ML R	R	R	R	-	CoP parameters had low-moderate test-retest reliability: RMS_displ_ (0.49–0.51), M_vel_ (0.68), range (0.46–0.49), MPF (0.41–0.49), D_short_ (0.52), D_long_ (0.03), & CP_time_ (0.32).	✓
Willigenburg et al. [31]	AP, ML	R	-	AP, ML	AP, ML	-	-	-	D_short_ (ML) was higher in LBP than control. LBP grabbed the safety rail more often than control. RMS_displ_, M_vel_, & D_short_ increased with EC.	✓
Larivière et al. [24]†	AP, ML R	AP, ML R	-	AP, ML R	R	R	R	-	High correlations (0.86–0.97) between outcomes measured by inertial sensors & optoelectronic system, inertial sensors & CoP, optoelectronic system & CoP. Many CoP measures had high test-retest reliability: RMS_displ_ (0.80–0.84), M_vel_ (0.76–0.86), MPF (0.80–0.82), D_short_ (0.81), D_long_ (0.74), & CP_time_ (0.64).	✓
Larivière et al. [34]	AP, ML R	AP, ML R	-	AP, ML R	-	-	-	-	No differences between LBP & control groups. RMS_displ_ (AP) was higher & MPF (AP, R) was lower in females than males.	✓
Sung et al. [19]	-	-	-	-	-	-	-	-	CoP area was higher in LBP than control. CoP area increased with EC (both groups) & greatest CoP deviation was seen in LBP during EC. No correlations between CoP area & LBP intensity, number of previous LBP episodes, duration of pain, & fear of movement.	✓
Shahvarpour et al. [75]*	-	-	-	-	-	-	-	-	No differences between LBP & control groups in seat kinematics (e.g., angular velocity/acceleration).	✓
Shahvarpour et al. [29]	AP, ML R	AP, ML R	-	AP, ML R	-	-	-	-	No differences between LBP & control groups at baseline. Moderate-high test-retest reliability for RMS_displ_ (0.77–0.84), M_vel_ (0.68–0.73), & MPF (0.75–0.82).	✓
Shahvarpour et al. [32]	AP, ML R	AP, ML R	-	AP, ML R	-	-	-	-	No differences between LBP & control groups. Lumbar belts increased RMS_displ_ (R) & decreased MPF (R).	✓
Cyr et al. [30]	AP, ML	R	-	-	-	-	-	-	M_vel_ (R) was higher in LBP than control (in EO, FB, & EC). RMS_displ_ (AP, ML) & M_vel_ (R) were higher in EC than EO & FB. RMS_displ_ (ML-FB) positively correlated with pain intensity.	✓
Larivière et al. [76]*	-	AP, ML	-	AP, ML	-	-	-	-	Postural control during unstable sitting can be used as a single proxy measure for determinants (mechanisms) associated with lumbar stability.	✓
van den Hoorn et al. [35]	AP, ML	AP, ML	-	AP, ML	AP, ML	AP, ML	AP, ML	AP, ML	RMS_displ_ (main effect), D_long_ (AP-EC), CP_time_ (main effect), & CP_dist_ (main effect) were higher in LBP than control. CoP velocity measures (RMS_vel_, D_short_) were not different between groups. No linear relations between CoP measures & LBP intensity, disability, & psychological features. Higher pain catastrophizing was associated with more safety bar touches. Higher CoP values were seen in males (RMS_displ_, M_vel_, D_short_, D_long_, CP_dist_), & associated with higher BMI (RMS_displ_, M_vel_, D_short_, CP_dist_), & more bar touches (RMS_displ_, M_vel_, MPF, D_short_, CP_time_, CP_dist_).	✓

**Abbreviations:** LBP, low back pain; RMS_displ_, root mean square displacement; M_vel_, mean velocity; MPF, mean power frequency; D_short_, short-term diffusion coefficient; D_long_, long-term diffusion coefficient; CP_time_, mean time coordinate of the critical point; CP_dist_, mean squared distance coordinate of the critical point; IPD, individual participant data; AP, anteroposterior; ML, mediolateral; R, resultant; SIL, seat instability level (higher level = higher difficulty); EC, eyes closed; CoP, center of pressure.

*IPD were not available as authors did not have access/authorisation to provide the IPD set.

^**†**^Studies that were only included in the descriptive analysis but excluded from quantitative/IPD analysis as these studies referred to an already identified IPD set

**Table 6:** Outcome measures and main findings for studies with data from only pain-free individuals.

Study	Primary measures		Secondary measures						Main findings	IPD
	RMS_displ_	M_vel_	Range	MPF	D_short_	D_long_	CP_time_	CP_dist_		
Cholewicki et al. [21]*	AP, ML	R	AP, ML	-	AP, ML R	AP, ML R	R	AP, ML R	All measures (all directions) increased when seat instability (task difficulty) increased. All measures correlated positively with age & weight. Moderate-high test-retest repeatability for RMS_displ_ (0.79–0.90), M_vel_ (0.91), range (0.77–0.91), D_short_ (0.91–0.96), D_long_ (0.56–0.57), CP_time_ (0.93), & CP_dist_ (0.89–0.96).	✓
Silfies et al. [77]	AP, ML	R	AP, ML	-	AP, ML R	AP, ML R	R	AP, ML R	RMS_displ_ (AP, ML), M_vel_ (R), range (AP, ML), D_short_ (AP, ML, R), D_long_ (AP, ML, R), & CP_dist_ (AP, ML, R) increased when seat instability increased & during EC (all except D_long_).	✓
van der Burg et al. [79]†	AP, ML	R	AP, ML	AP, ML	-	-	-	-	Non-relevant findings (Parkinson’s disease vs control).	☓
Reeves et al. [78]	-	AP, ML	-	-	-	-	-	-	M_vel_ (main effect) was higher with EC than EO. M_vel_ (main effect) was higher in trunk co-activation condition than control & arm co-activation conditions. M_vel_ (main effect) was lower in belt condition than trunk co-activation condition.	✓
Cholewicki et al. [80]	-	AP, ML R	-	-	-	-	-	-	No difference between groups with & without lumbosacral orthosis in M_vel_ (AP, ML, R).	✓
Lee and Granata [81]‡	AP, ML R	-	-	-	-	-	-	-	RMS_displ_ (AP, ML, R) had moderate-high (0.38–0.80) intra-session test-retest reliability. Intra-session test-retest reliability improved in more difficult seat instability conditions. Trial duration to achieve process stationarity was ≈30 seconds.	☓
Lee et al. [82]†	R	-	-	-	-	-	-	-	RMS_displ_ (R) increased as exertion force increased. Flexion exertions exhibited higher RMS_displ_ than extension exertions.	☓
Slota et al. [23]	AP, ML R	-	-	-	-	-	-	-	RMS_displ_ (AP, ML, R) increased after whole-body vibration.	✓
Hendershot & Nussbaum [83]	AP, ML	AP, ML	-	-	-	-	AP, ML	AP, ML	Non-relevant findings (lower-limb amputation vs control).	✓
Hendershot et al. [84]	AP, ML	AP, ML	-	-	AP, ML	-	-	-	All measures (AP, ML) increased following flexion exposure & increased further with increasing flexion exposures (e.g., longer flexion duration & presence of external load).	✓
Barbado et al. [86]*	-	-	-	-	-	-	-	-	Lack of significant correlations between trunk postural control & trunk strength/endurance.	✓
Barbado et al. [85]	-	-	-	-	-	-	-	-	Specialization in sports with large balance demands had a significant effect on trunk stability (e.g., competitive kayakers had better trunk postural control than recreational athletes).	✓
Beaudette et al. [87]	AP, ML	AP, ML	AP, ML	-	-	-	-	-	Non-relevant findings (treatment vs placebo).	✓
Ruggiero et al. [88]	AP, ML	AP, ML	-	-	-	-	-	-	RMS_displ_ (ML) & M_vel_ (AP) were lower when wearing the Kinesio tape compared to pre-tape time point. RMS_displ_ (AP, ML) & M_vel_ (ML, ML) were lower after the tape was removed compared to the pre-tape time point.	✓
Barbado et al. [28]	-	AP, ML	-	-	-	-	-	-	M_vel_ (AP, ML) showed a high (0.72–0.85) test-retest reliability. CoP analysis alone cannot adequately discern different postural strategies, as changes in balance performance (e.g., standard deviation of CoP) may not be accompanied by similar spine kinematic changes.	✓
Barbado et al. [89]*	-	-	-	-	-	-	-	-	Primary & secondary outcome measures were not reported.	✓
Glofcheskie & Brown [90]	AP, ML	-	AP, ML	-	-	-	-	-	RMS_displ_ (AP, ML) & range (AP, ML) were lower in golfers & runners than controls. Both athlete groups demonstrated greater trunk neuromuscular control (e.g., faster trunk muscle activation onsets) & better proprioceptive performance than control group.	✓
Acasio et al. [91]	AP, ML	R	-	-	-	-	-	-	RMS_displ_ (AP, ML) & M_vel_ (R) increased with higher seat instability level.	✓
Williams et al. [92]	AP, ML	AP, ML	-	-	AP, ML	-	-	-	Residuals between measurements of motion capture & inertial measurement unit were small (AP, ML). Higher RMS_displ_, M_vel_, & D_short_ were correlated (AP, ML) with greater height & weight. RMS_displ_, M_vel_, & D_short_ were higher with less stable seat (AP, ML) & EC (AP, ML). Vibrotactile feedback (EO) decreased RMS_displ_ (AP), M_vel_ (AP), & D_short_ (ML).	✓
Barbado et al. [93]†	-	-	-	-	-	-	-	-	Primary & secondary outcome measures were not reported.	☓
Roberts & Vette [25]	AP, ML	AP, ML	AP, ML	-	-	-	-	-	For all measures (AP, ML), seat movements were larger than pelvis & trunk movements. Trunk remained relatively stationary & stabilized by regulating seat (wobble board) movements.	✓
Roberts et al. [27]*	-	-	-	-	-	-	-	-	Wobble board-human system was stabilized through direction-specific activation of trunk & upper leg muscles that preceded seat (wobble board) displacement.	✓
Acasio et al. [94]*	AP, ML	R	-	-	-	-	-	-	Trunk-pelvic anti-phase movement increased (AP, ML) & trunk-pelvic in-phase movement decreased (AP, ML) when seat instability increased. Low (AP, ML) to moderate (AP) correlations between: CoP & trunk-pelvic anti-phase movement (+ correlation); CoP & trunk-pelvic in-phase movement (− correlation). Trunk coordination measures during unstable sitting directly quantified underlying movement strategies more than CoP measures.	✓
Alshehri et al. [26]*	-	-	-	-	-	-	-	-	Seat amplitude spectrum (movement) was higher (AP, ML) than hip/spine amplitude spectrums. In AP, amplitude spectrums of hip & lumbar segments were higher than other segments (lower lumbar, upper lumbar, thoracic), their coherence with the seat was high, & their motion was generally opposite in direction to the seat. In ML, amplitude spectrums of lower lumbar & lumbar segments, but not the hip, were higher than other segments (hip, upper lumbar, thoracic), their coherence with the seat was high, & their motion was generally opposite in direction to the seat. Although EC & higher BMI increased seat movements, this was associated with enhanced coordination (higher coherence) between hip/spine segments & the seat.	✓
de Oliveira et al. [95]‡	-	AP, ML	-	-	-	-	-	-	Non-relevant findings (exercise vs control).	☓

**Abbreviations:** RMS_displ_, root mean square displacement; M_vel_, mean velocity; MPF, mean power frequency; D_short_, short-term diffusion coefficient; D_long_, long-term diffusion coefficient; CP_time_, mean time coordinate of the critical point; CP_dist_, mean squared distance coordinate of the critical point; IPD, individual participant data; AP, anteroposterior; ML, mediolateral; R, resultant; EC, eyes closed; EO, eyes open.

*Studies that were only included in the descriptive analysis but excluded from quantitative/IPD analysis as these studies referred to an already identified IPD set.

^**†**^IPD were not available as authors did not have access/authorisation to provide the IPD set.

^**‡**^IPD were not available as authors did not respond to IPD request.

### 3.3. Quantitative analysis

#### 3.3.1. Included studies and participants

Twenty-six studies (1,097 participants) were included for the quantitative analysis. Twenty-four studies (1,050 participants) provided IPD sets [19, 22, 23, 25, 28–35, 73, 77, 78, 80, 83–85, 87, 88, 90–92] and were included for the IPD analysis. Nine studies (730 participants) were used for the IPD meta-analysis [19, 22, 29–35] to compare between individuals with (361 participants) and without (369 participants) LBP, and to test the effects of participant characteristics and vision on trunk postural control in relation to the difference between groups. Ten studies (379 participants) were used for the IPD meta-regression [19, 22, 29–35, 73] to test the effects of LBP clinical features on trunk postural control. The results of 17 studies that tested either only LBP (one study with IPD; 18 participants) or pain-free data (14 studies with IPD [23, 25, 28, 77, 78, 80, 83–85, 87, 88, 90–92]; 302 participants–two studies with aggregate data [79, 95]; 47 participants) were compared (visually using the mean plots) with the results of the IPD meta-analysis (individuals with and without LBP).

IPD for some participants (102 participants) were excluded from the analysis because of missing demographic information (23 participants) [19, 33, 80, 85, 90], or because they included participants who had no current pain (79 participants) but had a LBP history within the last 12 months [33] (S21 Table provides details of the excluded participants). For studies with multiple levels of seat difficulty, the level of seat difficulty was selected that matched closest with the seat build characteristics of other studies that included a single seat difficulty level (S22 Table provides details of the excluded seat difficulty levels).

Studies (*n* = 10/40) that referred to an already identified IPD set were excluded [21, 24, 26, 27, 74–76, 86, 89, 94]. Data for four studies that were considered for the quantitative analysis (either as IPD or aggregate data) were excluded from quantitative analysis because they did not report the required values (e.g., means) of any of the identified primary and secondary outcome measures [81, 93], or they only reported analysis of measures in the resultant direction [72, 82].

#### 3.3.2. IPD meta-analysis: Between-group differences

**Fig 3:**
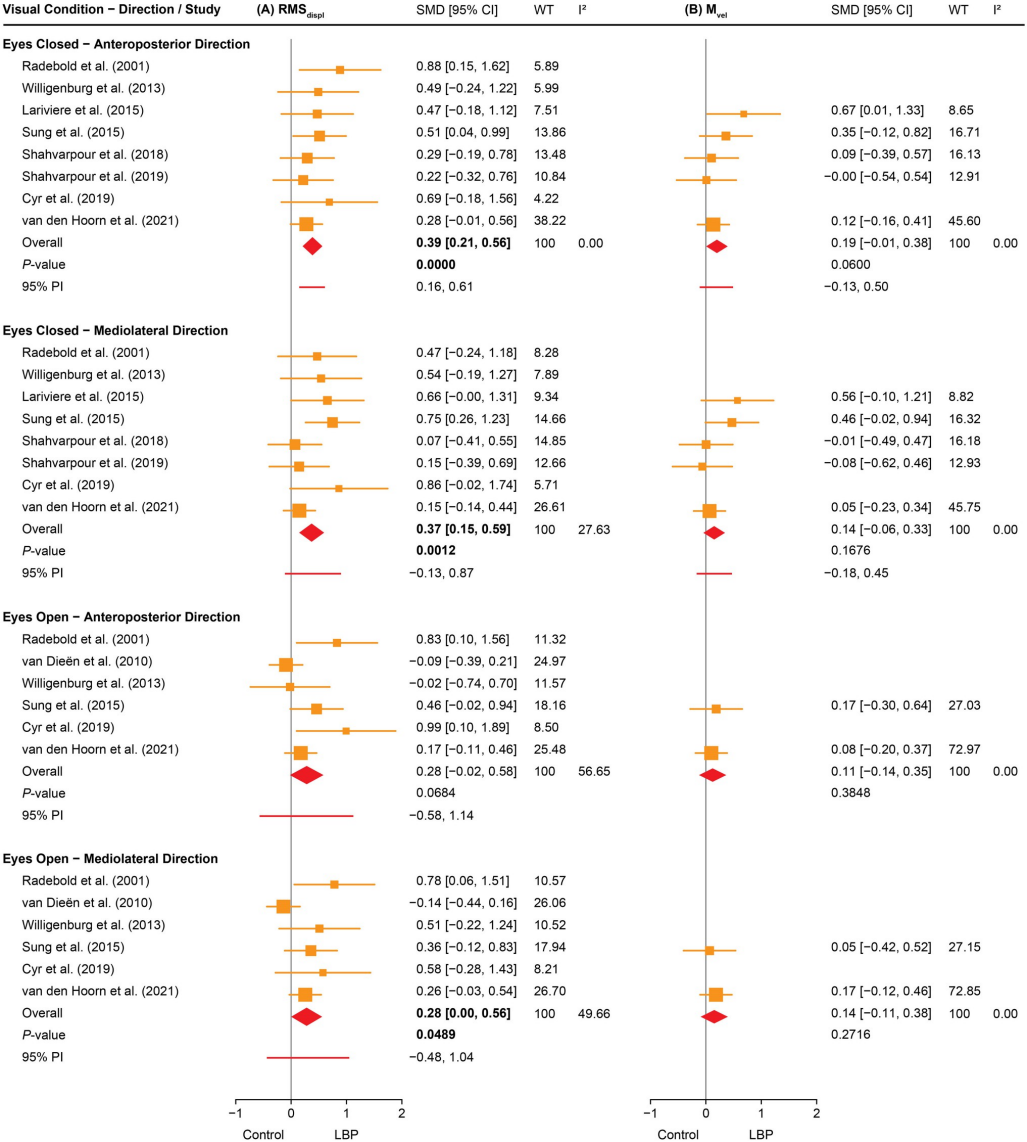
A two-stage individual participant data (IPD) meta-analysis, comparing individuals with and without low back pain (LBP) on primary outcome measures of trunk postural control. (A) root mean square displacement (RMS_displ_) and (B) mean velocity (M_vel_). The results are presented as standardised mean differences (SMDs) with 95% confidence intervals (95% CIs) using forest plots. Significant overall effect sizes with their respective P-values are highlighted in bold font. Sizing of squares reflects the weight (WT) of the contribution of a study on the pooled meta-analysis (weighted average) in percentage. I^2^ reflects the percentage of total variability due to heterogeneity between studies. 95% prediction interval (95% PI) reflects how much the effect size varies across studies.

IPD meta-analyses revealed significant differences between groups (all *P<*0.05). For primary outcome measures (Fig 3), individuals with LBP exhibited a higher RMS_displ_ than those without LBP during eyes closed in both the anteroposterior (SMD = 0.39, *P<*0.001, I^2^ = 0.00) and mediolateral (SMD = 0.37, *P<*0.01, I^2^ = 27.63) directions, and also during eyes open in the mediolateral direction (SMD = 0.28, *P<*0.05, I^2^ = 49.66). M_vel_ did not differ between individuals with and without LBP in any condition. Funnel plot asymmetry was significant for RMS_displ_ during eyes open in both directions (S4 Fig, all *P<*0.05).

For secondary outcome measures (see S1–S3 Figs for forest plots and S5–S7 Figs for funnel plots), individuals with LBP exhibited a higher range than those without LBP during both visual conditions and in both directions (SMD = 0.28–0.38, all *P<*0.05, I^2^ = 0.00–47.84; S1 Fig), lower MPF during eyes open in the anteroposterior direction (SMD = −0.23, *P<*0.05, I^2^ = 0.00; S1 Fig), and higher D_long_ during eyes closed in the anteroposterior direction (SMD = 0.44, *P<*0.01, I^2^ = 0.00; S2 Fig). Funnel plot asymmetry was significant for MPF in the mediolateral direction (eyes open; *P<*0.05; S5 Fig) and D_short_ in both the anteroposterior (eyes closed; *P<*0.05; S6 Fig) and mediolateral directions (eyes open; *P<*0.05; S6 Fig).

#### 3.3.3. Comparison between the outcomes of single-group studies with the outcome of two-group studies using IPD or aggregate data

**Fig 4:**
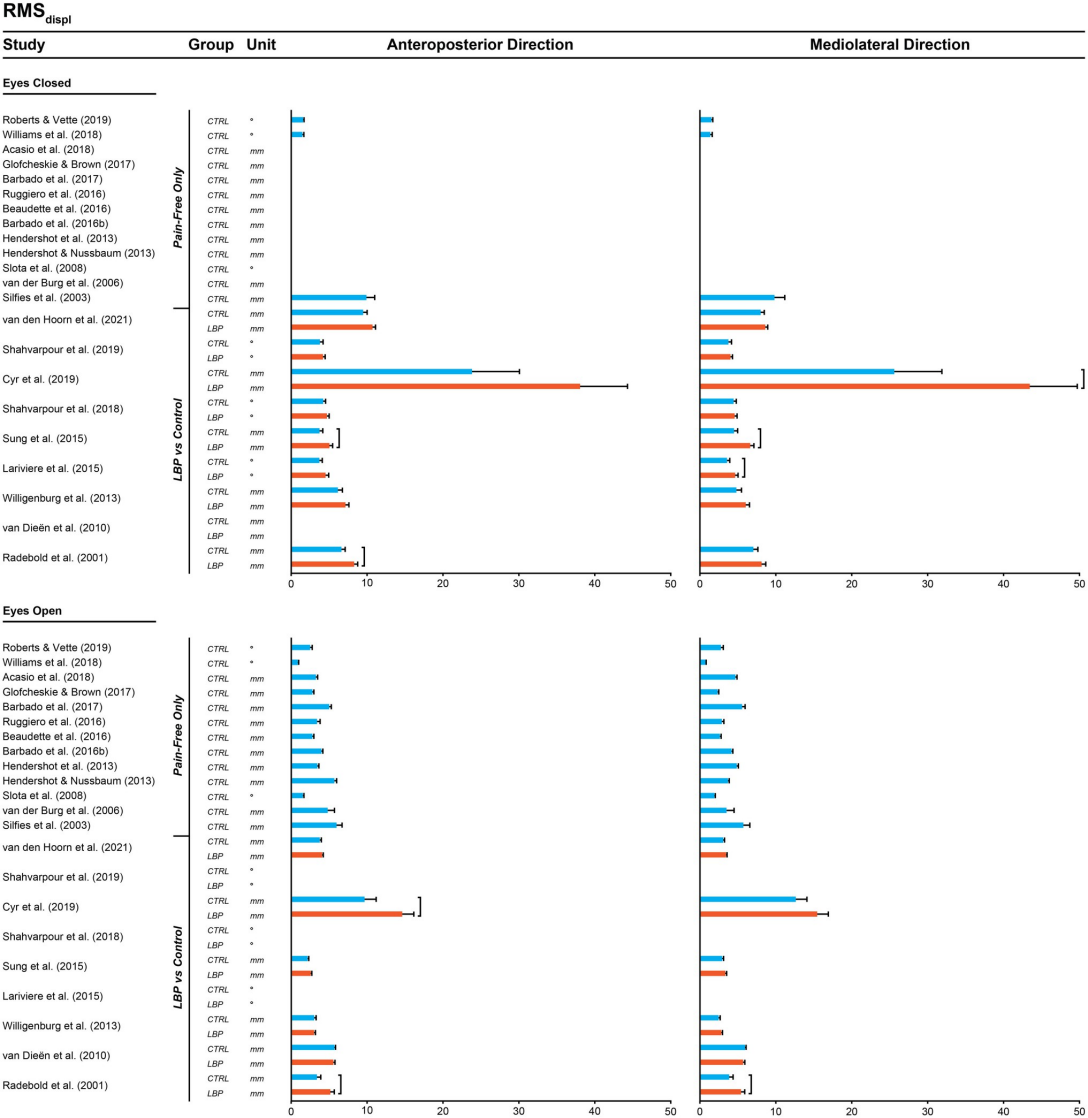
A quantitative (non-pooled) analysis of all studies on the root mean square displacement (RMS_displ_) of CoP/seat angle using IPD or aggregate data (if IPD were not available). Mean plots of the results from studies that were included in the individual participant data (IPD) meta-analysis (individuals with versus without low back pain [LBP]) are presented with mean plots of the results from studies that tested only pain-free individuals [CTRL]. The results are presented as means with standard errors. For studies with two groups, significant differences between individuals with and without LBP are shown with square bracket. No bars in some studies = no data available.

**Fig 5:**
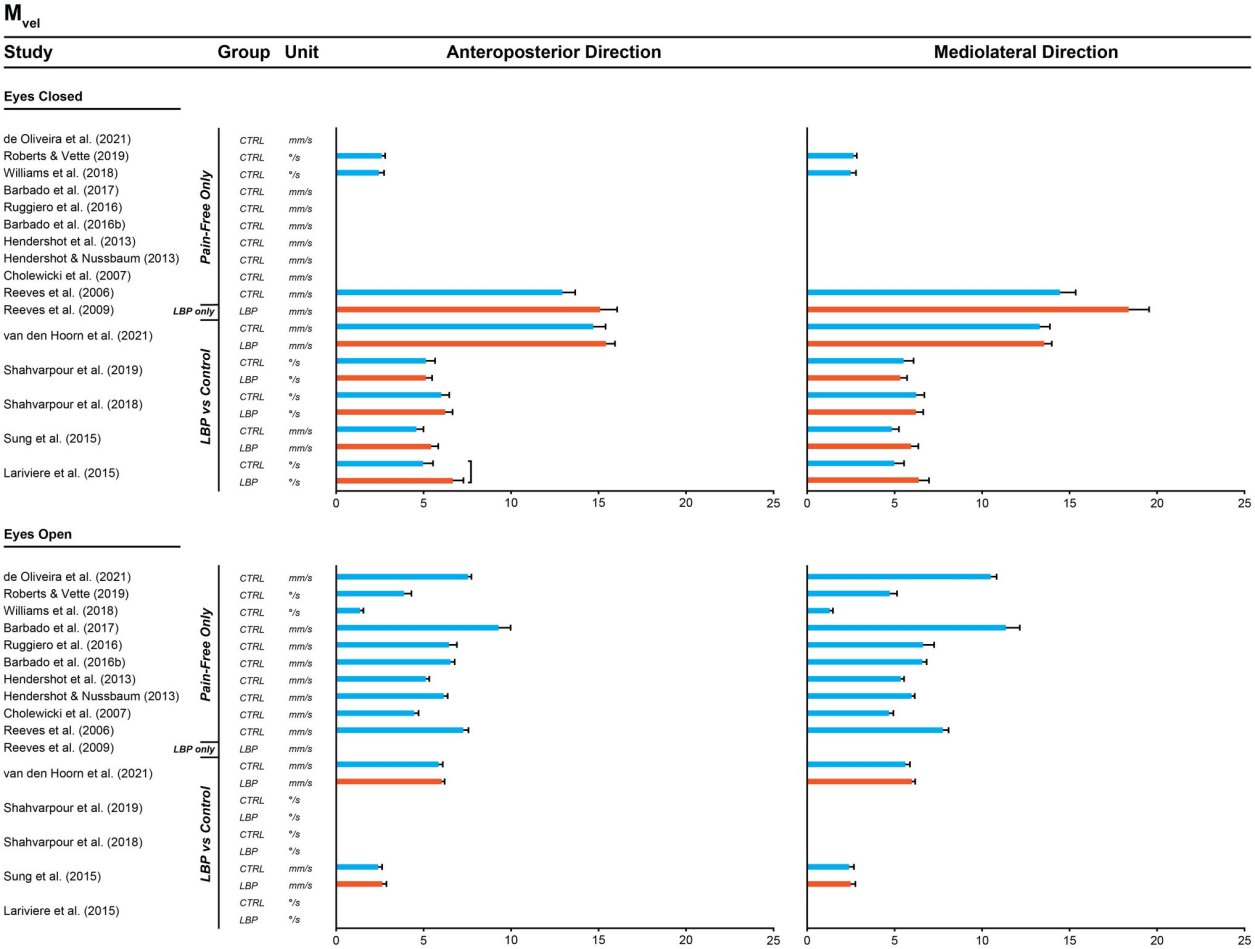
A quantitative (non-pooled) analysis of all studies on the mean velocity (M_vel_) of CoP/seat angle using IPD or aggregate data (if IPD were not available). Mean plots of the results from studies that were included in the individual participant data (IPD) meta-analysis (individuals with versus without low back pain [LBP]) are presented with mean plots of the results from studies that tested either only LBP or pain-free individuals [CTRL]. The results are presented as means with standard errors. For studies with two groups, significant differences between individuals with and without LBP are shown with square bracket. No bars in some studies = no data available.

Mean plots of the outcomes of studies with data from only LBP or pain-free individuals (using either IPD or aggregate data) are presented with mean plots of the outcomes of studies included in the IPD meta-analysis (individuals with and without LBP). For primary outcome measures, the visual inspection of mean plots of RMS_displ_ (Fig 4) and M_vel_ (Fig 5) revealed substantial variation in outcomes between studies renders comparisons between groups unclear for the eyes open condition in both directions. Few studies with data from only pain-free individuals were available for RMS_displ_ (*n* = 3) [25, 77, 92] and M_vel_ (*n* = 4) [25, 73, 78, 92] during eyes closed, and the results of outcomes differed considerably from studies with data including both individuals with and without LBP. One study with data from individual with only LBP [73] showed that M_vel_ was higher during eyes closed compared to most studies with data either from only pain-free individuals or individuals with versus without LBP in both directions (Fig 5). Similar observations can be made for secondary outcome measures (range, MPF, D_short_, D_long_, CP_time_, and CP_dist_), for which there were differences in outcomes when comparing studies with data from only pain-free individuals and studies with data from individuals with versus without LBP (see S8–S13 Figs for mean plots).

There was only one study [72] that included individuals with and without LBP for which IPD could not be obtained. That study reported that RMS_displ_ and D_short_ were higher among individuals with than without LBP in the resultant direction during the most difficult level of seat instability (50% R_spring_), which is similar to the results of the IPD meta-analysis. There were five studies [79, 81, 82, 93, 95] that included data from only pain-free individuals where IPD could not be obtained, but aggregate data were obtained for RMS_displ_ [79], M_vel_ [95], and range [79] from two studies [79, 95] (Figs 4 and 5 and S8 Fig). Neither IPD nor aggregate data could be obtained for the other studies [81, 82, 93], as two of these studies [81, 93] investigated different outcome measures, and one study [82] reported outcome measures only in the resultant direction. As such, it was it challenging to compare the results of these studies with studies included in the IPD meta-analysis.

After standardising the statistical analysis and including covariates in the models for all studies with available IPD, there were significant between-group differences for some outcome measures (RMS_displ_, range, and D_long_ were higher among individuals with than without LBP) in two studies [31, 34] that reported no differences between groups for the same outcome measures in the original published versions.

#### 3.3.4. IPD meta-analysis: Effects of participant characteristics

IPD meta-analyses revealed significant interaction effects with participant characteristics on some outcome measures (see S14–S21 Figs for forest plots). For the effects of age, the difference between groups was greater (worse effect for LBP group versus control group) in those with older age than those with younger age for D_short_ (eyes open; both directions; all *P<*0.05; S18 Fig) and CP_dist_ (eyes open; both directions; all *P<*0.05; S20 Fig). For the effects of BMI, the difference between groups was greater in those with higher BMI than those with lower BMI for CP_dist_ (eyes closed; mediolateral direction; *P<*0.01; S20 Fig) and CP_time_ (eyes open; mediolateral direction; *P<*0.05; S21 Fig). In contrast, for M_vel_, the difference between groups was greater in those with lower BMI than those with higher BMI (eyes closed; both directions; all *P<*0.05; S15 Fig).

#### 3.3.5. IPD meta-analysis: Effects of vision

**Fig 6:**
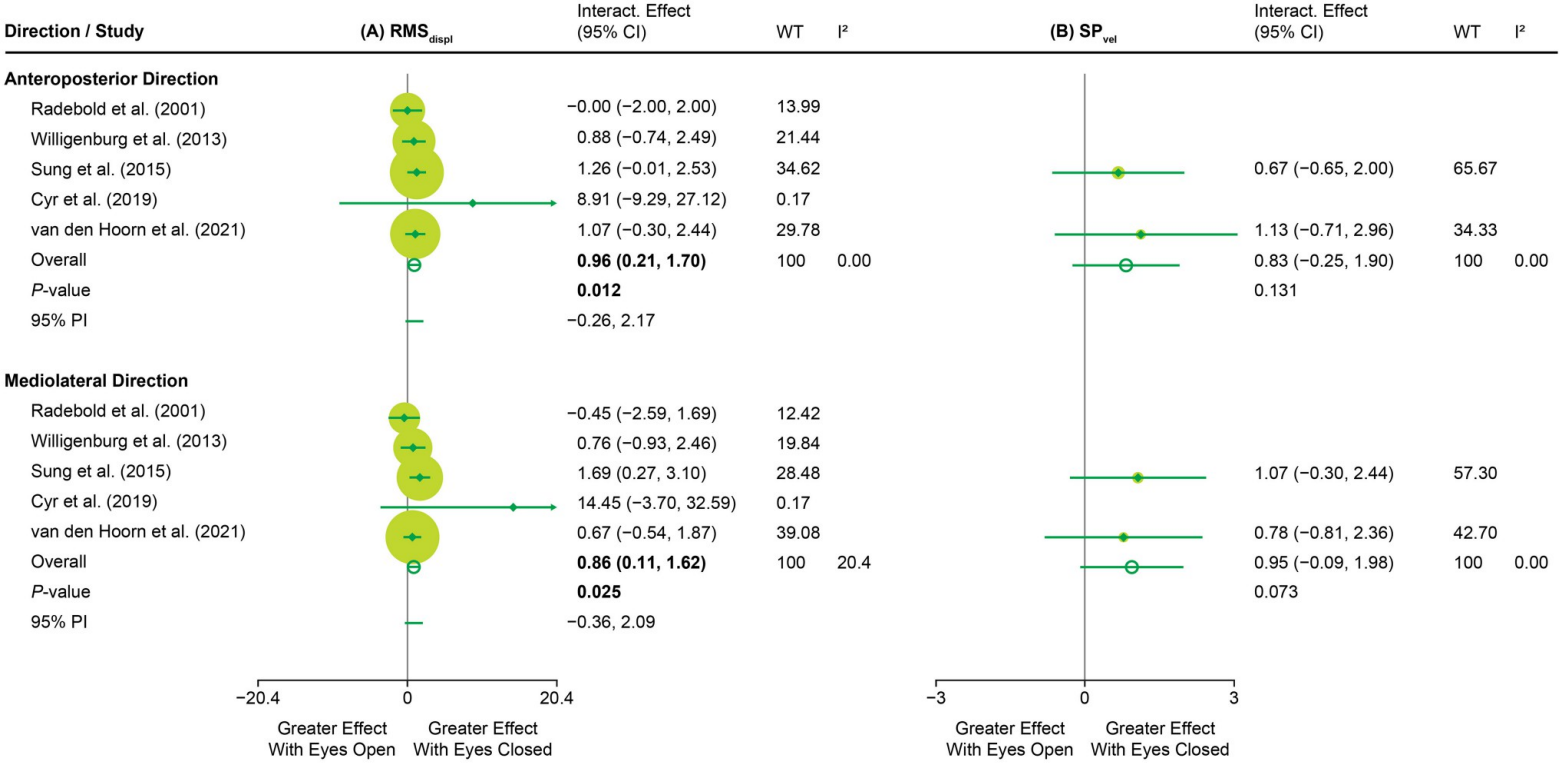
A two-stage individual participant data (IPD) meta-analysis of interactions between visual condition and the difference between groups on primary outcome measures of trunk postural control. (A) root mean square displacement (RMS_displ_) and (B) mean velocity (M_vel_). The results are presented as interaction (interact.) effect coefficients with 95% confidence intervals (95% CIs) using forest plots. Significant overall interact. effects with their respective P-values are highlighted in bold font. Greater effect in either directions indicates worse effect on trunk postural control for individuals with versus without low back pain. Sizing of circles reflects the weight (WT) of the contribution of a study on the pooled meta-analysis (weighted average) in percentage. I^2^ reflects the percentage of total variability due to heterogeneity between studies. 95% prediction interval (95% PI) reflects how much the effect size varies across studies.

IPD meta-analyses also revealed significant interaction effects with vision on some outcome measures. For primary outcome measures (Fig 6), the difference between groups was greater with eyes closed than eyes open for RMS_displ_ in both the anteroposterior and mediolateral directions (all *P<*0.05). For secondary outcome measures (see S22–S24 Figs for forest plots), the difference between groups was also greater with eyes closed than eyes open for D_short_ and D_long_ in the anteroposterior direction (all *P<*0.05; S23 Fig).

#### 3.3.6. IPD meta-regression: Effects of LBP clinical features

IPD meta-regressions revealed no significant associations (see S23 and S24 Tables) between LBP clinical features (VAS/NPRS, RMDQ, PCS, FABQ-PA, FABQ-W and FABQ) and either primary (RMS_displ_ and M_vel_) or secondary (range and MPF) outcome measures. Results of individual analysis for each study using IPD are presented in S25–S34 Tables.

## 4. Discussion

This systematic review with IPD meta-analyses had several major findings. First, IPD meta-analyses indicate that trunk postural control differs between individuals with and without LBP. A pooling of results from multiple studies showed that RMS_displ_, range and D_long_ were higher, and MPF was lower, among individuals with LBP, which can be concluded as indicating poorer postural control of the trunk. Second, IPD meta-analyses revealed that trunk postural control deteriorates more from removal of vision among individuals with than without LBP. Third, IPD meta-analyses revealed that older age and higher BMI have greater adverse impacts on trunk postural control among individuals with than without LBP. Fourth, IPD meta-regressions indicated that the limited clinical features that we could evaluate for the LBP group are not associated with poorer trunk postural control. Fifth, the visual inspection of mean plots indicated that the comparison between groups using the data from single-group studies (only LBP or pain-free data) with the data from two-group studies (LBP and pain-free data) was challenging, due to substantial variation in outcomes between studies, which suggests that the comparison between groups is only compelling when both groups are tested identically in the same study.

### 4.1. Experimental methods

Differences in experimental methods between studies comparing individuals with and without LBP that were included in the IPD meta-analysis can explain some of the inconsistency in study outcomes [19, 22, 29–35]. Important variation between studies includes differences in the seat apparatus–greater differences between groups were observed in studies that used the hemisphere-based seat [19, 22, 30, 31, 35] than the springs-based seat [29, 32, 34]. This is likely explained by differences in seat dynamics imposed by hemisphere-based seats (more challenging) versus springs-based seats (less challenging) when balancing. A lesser task difficulty might limit the potential to identify differences between groups [22, 72, 96]. The research group using the unstable springs-based sitting paradigm chose to use a not too difficult task (60% R_spring_) to help participants maintain balance long enough (60 s) to obtain reliable summary measures of trunk postural control [24]. It might be possible to slightly increase the difficulty of the task (e.g., from 60% to 50%), without affecting reliability too much, to increase the discriminative power of this test. Another possibility is to find the threshold of stability by finding the maximum task difficulty (e.g., … 50, 55, 60, 65, 70% R_spring_, …) in which stability can be maintained over a given time [97]. Removal of vision (eyes open versus eyes closed) also increases the challenge for trunk postural control, though this effect was not investigated in many studies [29, 32–34]. One study described the LBP group in limited detail (e.g., no information about pain intensity, disability, or other clinical features) [33]. These limitations reduced the total sample size that could be included in the IPD meta-analysis/meta-regression. As suggested by the analysis of covariates (see below), differences in participant characteristics might also explain some of the conflicting results between studies.

There are also several differences in experimental methods for studies with data from only LBP or pain-free individuals (single-group studies) that might explain the high differences in mean plots of the same outcome, including sample size, participant characteristics (e.g., sex, or anthropometrics), experimental setup (e.g., seat apparatus) and protocol (e.g., trial duration), and the data pre-processing (e.g., filters, data sampling for data reduction, etc.). These differences are greatly impacted on the comparison between groups, when we compared (visually using the mean plots) the outcomes of studies with data from only LBP or pain-free individuals with the outcomes of studies included in the IPD meta-analysis (individuals with and without LBP). This suggests that between-group comparison demands measurement within the same study with the same experimental parameters.

### 4.2. Differences in trunk postural control between individuals with and without LBP

IPD meta-analyses revealed that individuals with LBP had higher RMS_displ_, range, and D_long_ than those without LBP, suggesting greater CoP/seat movements and less corrective long-term behaviour [98–100]. Further, IPD meta-analyses indicated that some individuals with LBP had lower MPF, which could be explained by greater trunk stiffness [33]. Taken together, these findings suggest the presence of compromised trunk postural control among individuals with LBP, and which might be explained by multiple plausible mechanisms. For instance, less proprioceptive sensitivity would likely reduce the ability to coordinate the muscle responses (e.g., delayed trunk muscle responses) required to maintain balance in the unstable sitting task [22, 101], affecting the accuracy and precision of trunk movement [102], and also affecting the detection of movement errors to execute required postural adjustments [19, 103]. Together, these features would tend to destabilize trunk postural control as reflected by higher RMS_displ_, range, and D_long_. Alternatively, some individuals with LBP might adopt a distinct muscle activation strategy to control balance, such as increased muscle co-activation [78, 104, 105], either to reduce reliance on proprioception or to protect the spine from threat. Trunk stiffening would result in moving the trunk more as a whole and increase the moments of inertia on an unstable seat [79], resulting in lower sway frequency, and thus greater movements and stochastic activity of the CoP/seat. Decreased sense of lumbar proprioception [106–109], increased trunk stiffness [105, 110–112], and delayed onset or offset of trunk muscles activity [22, 42, 105, 113–115] have been widely documented in the LBP population. Regardless of the origin, impaired trunk postural control could be problematic for individuals with LBP and might expose them to the risk of sustaining or aggravating a back injury if maintained. For instance, A delay in response time would increase the vulnerability of spine to injury under sudden loading conditions [116], such as increasing tissue strain (further displacement) and stress (greater muscle force) of the spine [101]. Accordingly, an altered muscle recruitment pattern would be used as a compensation strategy [105]. However, adopting a co-contraction strategy also would increase spinal loads [117, 118].

### 4.3. Effects of vision

Corrective adjustments for postural control require contributions of the visual, vestibular, and proprioceptive (sense of positioning and movements) senses [119–121]. The demand for proprioceptive feedback (e.g., sensory receptors in spinal muscles, joints, and other tissues [73, 122]) is even greater in the absence of visual feedback. In line with this, IPD meta-analyses revealed that RMS_displ_, D_short_, and D_long_ were each increased by removal of vision, but this effect was greater for individuals with than without LBP, indicating greater CoP/seat movements and less tightly regulated behaviour [98–100]. This implies that, without visual feedback, the quality of trunk postural control is reduced to a greater extent with LBP, as it potentially cannot be adequately compensated for by other alternative sensory sources. Control of CoP/seat diffusion rate relies on adequate and timely sensory information [123]. Moving the CoP/seat further away from a relative equilibrium point over shorter periods of time in the LBP group might be a strategy to overcome higher sensory thresholds caused by LBP injury, and thus would enhance feedback [22, 98], or a strategy to increase muscle stiffness to promote rapid postural corrections [77, 78, 124]. Further, fewer corrective adjustments for CoP/seat movements over long-term intervals of time observed in the LBP group may due to lower proprioceptive sensitivity [22, 35, 101] or deficits in sensory reweighting [35, 77]. This conjecture is consistent with previous data that show individuals with LBP could not modulate and reweight their sensory information to other alternative sources (most likely proprioceptive feedback) when visual feedback was unavailable [22, 35].

### 4.4. Effects of participant characteristics

IPD meta-analyses revealed that older age and higher BMI more negatively impacted trunk postural control for those with than without LBP. The findings of higher D_short_ and longer CP_dist_ and CP_time_ indicate that the CoP/seat moved further within the short-term region, with a later critical point (the point at which corrective responses take place to slow CoP/seat diffusion rate and limit further displacement of CoP/seat [98, 99]) among individuals with LBP who were older or had higher BMI. These findings might be explained by age-related changes in muscle mass [125, 126], proprioception [127], muscle response [103], or sensory thresholds [103]. Higher body mass that is placed above the radius of a hemisphere creates a mechanically more challenging system to balance [21]. Higher body mass is also likely to impose biomechanical constraints that require greater muscular activity and torque [128] and interfere with the coordination of joints and muscles due to changes in anthropometrics [129]. The impacts of these factors might be accentuated for the LBP group, who already have impaired balance. Although anthropometric characteristics (height and weight) may influence measures of trunk postural control during unstable sitting [21, 24], IPD meta-analyses confirmed differences between groups while adjusting for these confounding variables.

### 4.5. Effects of LBP clinical features

Although we had limited data available, IPD meta-regressions indicated that LBP intensity, disability, pain catastrophizing, and fear of movement were not associated with poorer trunk postural control among individuals with LBP. It should be noted, though, that individuals with LBP in most studies had low scores on disability, catastrophizing, and fear avoidance. It is unlikely that our findings are explained by insufficient time for these features to develop [35, 130], since several studies included participants who suffered from LBP for long periods [22, 29–32, 34, 73]. Greater detail and consistent collection of clinical features would have made findings more robust.

### 4.6. Implications

Our IPD meta-analyses confirm that trunk postural control is different between individuals with and without LBP. This justified future evaluation whether interventions that address trunk postural control might be helpful in the management of LBP. The current review identified some weaknesses in the available IPD sets that should be addressed in future research. For example, the evaluation of clinical and related features of LBP such as pain intensity, disability level, and psychological features should be included in studies of postural control—many studies included in this review did not asses these features and this limited the capacity to evaluate the impact of these features on trunk postural control. Longitudinal studies including investigation of different stages of LBP (time) are required to determine whether the differences in trunk postural control are adaptive or maladaptive. This review also identified that the outcome measures used to evaluate trunk postural control provide limited insight into the interpretation of differences in trunk postural control and new research could focus on how the trunk is controlled (e.g., coordination of different spine regions/segments), not just a general output that is included in this IPD meta-analysis (e.g., CoP/seat motion). This could provide more information on potential modifiable factors for treatment.

### 4.7. Limitations

This review has several limitations that require consideration. First, IPD could not be obtained for six studies, although the impact of this will be limited as only one of those studies included data from individuals with versus without LBP. Second, overall SMD effect sizes for between-group differences were small or close to medium (maybe due to the small number of included studies), and 95% PIs were wider in some IPD meta-analyses (likely due to lack of precision). Third, there was evidence of small-study effects (funnel plots asymmetry) which suggests some publication bias. However, the results should be interpreted with caution as the number of included studies in all IPD meta-analyses was <10 and in many cases with secondary outcome measures there were <5 studies. This is just below the threshold of 10 studies that is considered necessary to detect funnel plots asymmetry [131]. The funnel plots asymmetry detected for several measures might be explained by the small number of included studies and between-study heterogeneity [131] rather than publication bias. Fourth, the effect of LBP duration (e.g., acute, sub-acute, chronic) on trunk postural control could not be evaluated in the IPD meta-analysis because of between-study variation in how these features were defined (or not at all), and an overall very few studies included individuals with acute LBP. Fifth, the effect of task difficulty (based on seat apparatus and its build characteristics) on trunk postural control between groups could not be examined because of the limited number of studies that used springs-base seats. Sixth, although some data from our IPD meta-analysis might have violated the normality assumptions, we used multilevel mixed-effects models in the first stage of our two-stage IPD meta-analysis which are robust to violations of normality assumptions [132]. Seventh, we limited our review to include only those studies that were published in English.

## 5. Conclusions

This IPD analysis provides robust evidence that trunk postural control is compromised among individuals with LBP. The findings are likely explained by delayed or less accurate corrective responses, for which there are several plausible mechanisms–impaired proprioception, altered sensory processing/reweighting, or increased muscle co-activation. We cannot discriminate whether changes in trunk postural control are a cause or consequence of LBP. Our investigation highlights the value of IPD analysis to draw robust conclusions from biomechanical data, but also brings to light the affect variation in available (limited) data has on addressing important questions related to the association with LBP clinical and related features.

## Supporting information

S1 Checklist PRISMA-IPD checklist. (DOCX)

S1 Table Search strategy used in MEDLINE and CINAHL databases. (DOCX)

S2 Table Search strategy used in Embase database. (DOCX)

S3 Table Search strategy used in Scopus database. (DOCX)

S4 Table Search strategy used in Web of Science Core Collection database. (DOCX)

S5 Table Updated search strategy used in MEDLINE and CINAHL databases. (DOCX)

S6 Table Updated search strategy used in Embase database. (DOCX)

S7 Table Updated search strategy used in Scopus database. (DOCX)

S8 Table Updated search strategy used in Web of Science Core Collection database. (DOCX)

S9 Table A checklist for comprehensiveness of reporting and methodological quality. (DOCX)

S10 Table List of data used for the descriptive analysis. (DOCX)

S11 Table Inclusion and exclusion criteria for studies with data from individuals with LBP. (DOCX)

S12 Table Inclusion and exclusion criteria for studies with data from only pain-free individuals. (DOCX)

S13 Table Experimental setup and protocol used in studies with data from individuals with LBP. (DOCX)

S14 Table Experimental setup and protocol used in studies with data from only pain-free individuals. (DOCX)

S15 Table Reporting/Quality scores for studies with data from individuals with LBP: Available from published papers. (DOCX)

S16 Table Reporting/Quality scores for studies with data from individuals with LBP: Available from IPD. (DOCX)

S17 Table Reporting/Quality scores for studies with data from only pain-free individuals: Available from published papers. (DOCX)

S18 Table Reporting/Quality scores for studies with data from only pain-free individuals: Available from IPD. (DOCX)

S19 Table Reporting/Quality total scores for studies with data from individuals with LBP: Published papers versus IPD. (DOCX)

S20 Table Reporting/Quality total scores for studies with data from only pain-free individuals: Published papers versus IPD. (DOCX)

S21 Table Excluded participants from the IPD analysis. (DOCX)

S22 Table Included and excluded levels of the seat difficulty in the IPD analysis. (DOCX)

S23 Table A two-stage IPD meta-regression of associations between LBP intensity or disability and trunk postural control. (DOCX)

S24 Table A two-stage IPD meta-regression of associations between pain catastrophizing or fear-avoidance beliefs and trunk postural control. (DOCX)

S25 Table Individual IPD analysis of associations between LBP intensity or disability and RMS_displ_ for each study. (DOCX)

S26 Table Individual IPD analysis of associations between pain catastrophizing or fear-avoidance beliefs and RMS_displ_ for each study. (DOCX)

S27 Table Individual IPD analysis of associations between LBP intensity or disability and M_vel_ for each study. (DOCX)

S28 Table Individual IPD analysis of associations between pain catastrophizing or fear-avoidance beliefs and M_vel_ for each study. (DOCX)

S29 Table Individual IPD analysis of associations between LBP intensity or disability and range for each study. (DOCX)

S30 Table Individual IPD analysis of associations between pain catastrophizing or fear-avoidance beliefs and range for each study. (DOCX)

S31 Table Individual IPD analysis of associations between LBP intensity or disability and MPF for each study. (DOCX)

S32 Table Individual IPD analysis of associations between pain catastrophizing or fear-avoidance beliefs and MPF for each study. (DOCX)

S33 Table Individual IPD analysis of associations between LBP intensity or disability and stabilogram diffusion measures* for each study. (DOCX)

S34 Table Individual IPD analysis of associations between pain catastrophizing or fear-avoidance beliefs and stabilogram diffusion measures* for each study. (DOCX)

S1 Fig A two-stage individual participant data (IPD) meta-analysis, comparing individuals with and without low back pain (LBP) on secondary outcome measures of trunk postural control. (A) range and (B) mean power frequency (MPF). The results are presented as standardised mean differences (SMDs) with 95% confidence intervals (95% CIs) using forest plots. Significant overall effect sizes with their respective P-values are highlighted in bold font. Sizing of squares reflects the weight (WT) of the contribution of a study on the pooled meta-analysis (weighted average) in percentage. I^2^ reflects the percentage of total variability due to heterogeneity between studies. 95% prediction interval (95% PI) reflects how much the effect size varies across studies.
(JPG)

S2 Fig A two-stage individual participant data (IPD) meta-analysis, comparing individuals with and without low back pain (LBP) on secondary outcome measures of trunk postural control. (A) short-term diffusion (D_short_) and (B) long-term diffusion (D_long_). The results are presented as standardised mean differences (SMDs) with 95% confidence intervals (95% CIs) using forest plots. Significant overall effect sizes with their respective P-values are highlighted in bold font. Sizing of squares reflects the weight (WT) of the contribution of a study on the pooled meta-analysis (weighted average) in percentage. I^2^ reflects the percentage of total variability due to heterogeneity between studies. 95% prediction interval (95% PI) reflects how much the effect size varies across studies.
(JPG)

S3 Fig A two-stage individual participant data (IPD) meta-analysis, comparing individuals with and without low back pain (LBP) on secondary outcome measures of trunk postural control. (A) mean squared distance coordinate of the critical point (CP_dist_) and (B) mean time coordinate of the critical point (CP_time_). The results are presented as standardised mean differences (SMDs) with 95% confidence intervals (95% CIs) using forest plots. Significant overall effect sizes with their respective P-values are highlighted in bold font. Sizing of squares reflects the weight (WT) of the contribution of a study on the pooled meta-analysis (weighted average) in percentage. I^2^ reflects the percentage of total variability due to heterogeneity between studies. 95% prediction interval (95% PI) reflects how much the effect size varies across studies.
(JPG)

S4 Fig Funnel plots to test the potential presence of small-study effects (funnel plot asymmetry) in the two-stage individual participant data (IPD) meta-analysis of primary outcome measures. (A) root mean square displacement (RMS_displ_) and (B) mean velocity (M_vel_). The vertical solid red lines represent the overall standardized mean differences (SMDs) from the IPD meta-analysis. The two diagonal dashed lines in both sides represent the pseudo 95% confidence intervals (95% CIs) around the overall SMDs for each standard error (precision). Each orange dot represents a SMD for an individual study. Small studies are scattered more widely at the bottom of the funnel plot (lower precision) and larger studies are scattered more at the top of the funnel plot (greater precision). P-values for the potential presence of significant small-study effects (funnel plot asymmetry) are highlighted in bold font.
(JPG)

S5 Fig Funnel plots to test the potential presence of small-study effects (funnel plot asymmetry) in the two-stage individual participant data (IPD) meta-analysis of secondary outcome measures. (A) range and (B) mean power frequency (MPF). The vertical solid red lines represent the overall standardized mean differences (SMDs) from the IPD meta-analysis. The two diagonal dashed lines in both sides represent the pseudo 95% confidence intervals (95% CIs) around the overall SMDs for each standard error (precision). Each orange dot represents a SMD for an individual study. Small studies are scattered more widely at the bottom of the funnel plot (lower precision) and larger studies are scattered more at the top of the funnel plot (greater precision). P-values for the potential presence of significant small-study effects (funnel plot asymmetry) are highlighted in bold font.
(JPG)

S6 Fig Funnel plots to test the potential presence of small-study effects (funnel plot asymmetry) in the two-stage individual participant data (IPD) meta-analysis of secondary outcome measures. (A) short-term diffusion (D_short_) and (B) long-term diffusion (D_long_). The vertical solid red lines represent the overall standardized mean differences (SMDs) from the IPD meta-analysis. The two diagonal dashed lines in both sides represent the pseudo 95% confidence intervals (95% CIs) around the overall SMDs for each standard error (precision). Each orange dot represents a SMD for an individual study. Small studies are scattered more widely at the bottom of the funnel plot (lower precision) and larger studies are scattered more at the top of the funnel plot (greater precision). P-values for the potential presence of significant small-study effects (funnel plot asymmetry) are highlighted in bold font.
(JPG)

S7 Fig Funnel plots to test the potential presence of small-study effects (funnel plot asymmetry) in the two-stage individual participant data (IPD) meta-analysis of secondary outcome measures. (A) mean squared distance coordinate of the critical point (CP_dist_) and (B) mean time coordinate of the critical point (CP_time_). The vertical solid red lines represent the overall standardized mean differences (SMDs) from the IPD meta-analysis. The two diagonal dashed lines in both sides represent the pseudo 95% confidence intervals (95% CIs) around the overall SMDs for each standard error (precision). Each orange dot represents a SMD for an individual study. Small studies are scattered more widely at the bottom of the funnel plot (lower precision) and larger studies are scattered more at the top of the funnel plot (greater precision). P-values for the potential presence of significant small-study effects (funnel plot asymmetry) are highlighted in bold font.
(JPG)

S8 Fig A quantitative (non-pooled) analysis of all studies on the range of CoP/seat angle using IPD or aggregate data (if IPD were not available). Mean plots of the results from studies that were included in the individual participant data (IPD) meta-analysis (individuals with versus without low back pain [LBP]) are presented with mean plots of the results from studies that tested only pain-free individuals [CTRL]. The results are presented as means with standard errors. For studies with two groups, significant differences between individuals with and without LBP are shown with square bracket. No bars in some studies = no data available.
(JPG)

S9 Fig A quantitative (non-pooled) analysis of all studies on the mean power frequency (MPF) of CoP/seat angle using IPD or aggregate data (if IPD were not available). Mean plots of the results from studies that were included in the individual participant data (IPD) meta-analysis (individuals with versus without low back pain [LBP]) are presented with mean plots of the results from studies that tested only pain-free individuals [CTRL]. The results are presented as means with standard errors. For studies with two groups, significant differences between individuals with and without LBP are shown with square bracket. No bars in some studies = no data available.
(JPG)

S10 Fig A quantitative (non-pooled) analysis of all studies on the short-term diffusion (D_short_) of CoP/seat angle using IPD or aggregate data (if IPD were not available). Mean plots of the results from studies that were included in the individual participant data (IPD) meta-analysis (individuals with versus without low back pain [LBP]) are presented with mean plots of the results from studies that tested only pain-free individuals [CTRL]. The results are presented as means with standard errors. For studies with two groups, significant differences between individuals with and without LBP are shown with square bracket. No bars in some studies = no data available.
(JPG)

S11 Fig A quantitative (non-pooled) analysis of all studies on the long-term diffusion (D_long_) of CoP/seat angle using IPD or aggregate data (if IPD were not available). Mean plots of the results from studies that were included in the individual participant data (IPD) meta-analysis (individuals with versus without low back pain [LBP]) are presented with mean plots of the results from studies that tested only pain-free individuals [CTRL]. The results are presented as means with standard errors. For studies with two groups, significant differences between individuals with and without LBP are shown with square bracket. No bars in some studies = no data available.
(JPG)

S12 Fig A quantitative (non-pooled) analysis of all studies on the mean squared distance coordinate of the critical point (CP_dist_) of CoP/seat angle using IPD or aggregate data (if IPD were not available). Mean plots of the results from studies that were included in the individual participant data (IPD) meta-analysis (individuals with versus without low back pain [LBP]) are presented with mean plots of the results from studies that tested only pain-free individuals [CTRL]. The results are presented as means with standard errors. For studies with two groups, significant differences between individuals with and without LBP are shown with square bracket. No bars in some studies = no data available.
(JPG)

S13 Fig A quantitative (non-pooled) analysis of all studies on the mean time coordinate of the critical point (CP_time_) of CoP/seat angle using IPD or aggregate data (if IPD were not available). Mean plots of the results from studies that were included in the individual participant data (IPD) meta-analysis (individuals with versus without low back pain [LBP]) are presented with mean plots of the results from studies that tested only pain-free individuals [CTRL]. The results are presented as means with standard errors. For studies with two groups, significant differences between individuals with and without LBP are shown with square bracket. No bars in some studies = no data available.
(JPG)

S14 Fig A two-stage individual participant data (IPD) meta-analysis of interactions between participant characteristics and the difference between groups on the root mean square displacement (RMS_displ_) of CoP/seat angle. (A) age, (B) sex, and (C) body mass index (BMI). The results are presented as interaction (interact.) effect coefficients with 95% confidence intervals (95% CIs) using forest plots. Significant overall interact. effects with their respective P-values are highlighted in bold font. Greater effect in either directions indicates worse effect on trunk postural control for individuals with versus without low back pain. Sizing of circles reflects the weight (WT) of the contribution of a study on the pooled meta-analysis (weighted average) in percentage. I^2^ reflects the percentage of total variability due to heterogeneity between studies. 95% prediction interval (95% PI) reflects how much the effect size varies across studies.
(JPG)

S15 Fig A two-stage individual participant data (IPD) meta-analysis of interactions between participant characteristics and the difference between groups on the mean velocity (M_vel_) of CoP/seat angle. (A) age, (B) sex, and (C) body mass index (BMI). The results are presented as interaction (interact.) effect coefficients with 95% confidence intervals (95% CIs) using forest plots. Significant overall interact. effects with their respective P-values are highlighted in bold font. Greater effect in either directions indicates worse effect on trunk postural control for individuals with versus without low back pain. Sizing of circles reflects the weight (WT) of the contribution of a study on the pooled meta-analysis (weighted average) in percentage. I^2^ reflects the percentage of total variability due to heterogeneity between studies. 95% prediction interval (95% PI) reflects how much the effect size varies across studies.
(JPG)

S16 Fig A two-stage individual participant data (IPD) meta-analysis of interactions between participant characteristics and the difference between groups on the range of CoP/seat angle. (A) age, (B) sex, and (C) body mass index (BMI). The results are presented as interaction (interact.) effect coefficients with 95% confidence intervals (95% CIs) using forest plots. Significant overall interact. effects with their respective P-values are highlighted in bold font. Greater effect in either directions indicates worse effect on trunk postural control for individuals with versus without low back pain. Sizing of circles reflects the weight (WT) of the contribution of a study on the pooled meta-analysis (weighted average) in percentage. I^2^ reflects the percentage of total variability due to heterogeneity between studies. 95% prediction interval (95% PI) reflects how much the effect size varies across studies.
(JPG)

S17 Fig A two-stage individual participant data (IPD) meta-analysis of interactions between participant characteristics and the difference between groups on the mean power frequency (MPF) of CoP/seat angle. (A) age, (B) sex, and (C) body mass index (BMI). The results are presented as interaction (interact.) effect coefficients with 95% confidence intervals (95% CIs) using forest plots. Significant overall interact. effects with their respective P-values are highlighted in bold font. Greater effect in either directions indicates worse effect on trunk postural control for individuals with versus without low back pain. Sizing of circles reflects the weight (WT) of the contribution of a study on the pooled meta-analysis (weighted average) in percentage. I^2^ reflects the percentage of total variability due to heterogeneity between studies. 95% prediction interval (95% PI) reflects how much the effect size varies across studies.
(JPG)

S18 Fig A two-stage individual participant data (IPD) meta-analysis of interactions between participant characteristics and the difference between groups on the short-term diffusion (D_short_) of CoP/seat angle. (A) age, (B) sex, and (C) body mass index (BMI). The results are presented as interaction (interact.) effect coefficients with 95% confidence intervals (95% CIs) using forest plots. Significant overall interact. effects with their respective P-values are highlighted in bold font. Greater effect in either directions indicates worse effect on trunk postural control for individuals with versus without low back pain. Sizing of circles reflects the weight (WT) of the contribution of a study on the pooled meta-analysis (weighted average) in percentage. I^2^ reflects the percentage of total variability due to heterogeneity between studies. 95% prediction interval (95% PI) reflects how much the effect size varies across studies.
(JPG)

S19 Fig A two-stage individual participant data (IPD) meta-analysis of interactions between participant characteristics and the difference between groups on the long-term diffusion (D_long_) of CoP/seat angle. (A) age, (B) sex, and (C) body mass index (BMI). The results are presented as interaction (interact.) effect coefficients with 95% confidence intervals (95% CIs) using forest plots. Significant overall interact. effects with their respective P-values are highlighted in bold font. Greater effect in either directions indicates worse effect on trunk postural control for individuals with versus without low back pain. Sizing of circles reflects the weight (WT) of the contribution of a study on the pooled meta-analysis (weighted average) in percentage. I^2^ reflects the percentage of total variability due to heterogeneity between studies. 95% prediction interval (95% PI) reflects how much the effect size varies across studies.
(JPG)

S20 Fig A two-stage individual participant data (IPD) meta-analysis of interactions between participant characteristics and the difference between groups on the mean squared distance coordinate of the critical point (CP_dist_) of CoP/seat angle. (A) age, (B) sex, and (C) body mass index (BMI). The results are presented as interaction (interact.) effect coefficients with 95% confidence intervals (95% CIs) using forest plots. Significant overall interact. effects with their respective P-values are highlighted in bold font. Greater effect in either directions indicates worse effect on trunk postural control for individuals with versus without low back pain. Sizing of circles reflects the weight (WT) of the contribution of a study on the pooled meta-analysis (weighted average) in percentage. I^2^ reflects the percentage of total variability due to heterogeneity between studies. 95% prediction interval (95% PI) reflects how much the effect size varies across studies.
(JPG)

S21 Fig A two-stage individual participant data (IPD) meta-analysis of interactions between participant characteristics and the difference between groups on the mean time coordinate of the critical point (CP_time_) of CoP/seat angle. (A) age, (B) sex, and (C) body mass index (BMI). The results are presented as interaction (interact.) effect coefficients with 95% confidence intervals (95% CIs) using forest plots. Significant overall interact. effects with their respective P-values are highlighted in bold font. Greater effect in either directions indicates worse effect on trunk postural control for individuals with versus without low back pain. Sizing of circles reflects the weight (WT) of the contribution of a study on the pooled meta-analysis (weighted average) in percentage. I^2^ reflects the percentage of total variability due to heterogeneity between studies. 95% prediction interval (95% PI) reflects how much the effect size varies across studies.
(JPG)

S22 Fig A two-stage individual participant data (IPD) meta-analysis of interactions between visual condition and the difference between groups on secondary outcome measures of trunk postural control. (A) range and (B) mean power frequency (MPF). The results are presented as interaction (interact.) effect coefficients with 95% confidence intervals (95% CIs) using forest plots. Significant overall interact. effects with their respective P-values are highlighted in bold font. Greater effect in either directions indicates worse effect on trunk postural control for individuals with versus without low back pain. Sizing of circles reflects the weight (WT) of the contribution of a study on the pooled meta-analysis (weighted average) in percentage. I^2^ reflects the percentage of total variability due to heterogeneity between studies. 95% prediction interval (95% PI) reflects how much the effect size varies across studies.
(JPG)

S23 Fig A two-stage individual participant data (IPD) meta-analysis of interactions between visual condition and the difference between groups on secondary outcome measures of trunk postural control. (A) short-term diffusion (D_short_) and (B) long-term diffusion (D_long_). The results are presented as interaction (interact.) effect coefficients with 95% confidence intervals (95% CIs) using forest plots. Significant overall interact. effects with their respective P-values are highlighted in bold font. Greater effect in either directions indicates worse effect on trunk postural control for individuals with versus without low back pain. Sizing of circles reflects the weight (WT) of the contribution of a study on the pooled meta-analysis (weighted average) in percentage. I^2^ reflects the percentage of total variability due to heterogeneity between studies. 95% prediction interval (95% PI) reflects how much the effect size varies across studies.
(JPG)

S24 Fig A two-stage individual participant data (IPD) meta-analysis of interactions between visual condition and the difference between groups on secondary outcome measures of trunk postural control. (A) mean squared distance coordinate of the critical point (CP_dist_) and (B) mean time coordinate of the critical point (CP_time_). The results are presented as interaction (interact.) effect coefficients with 95% confidence intervals (95% CIs) using forest plots. Significant overall interact. effects with their respective P-values are highlighted in bold font. Greater effect in either directions indicates worse effect on trunk postural control for individuals with versus without low back pain. Sizing of circles reflects the weight (WT) of the contribution of a study on the pooled meta-analysis (weighted average) in percentage. I^2^ reflects the percentage of total variability due to heterogeneity between studies. 95% prediction interval (95% PI) reflects how much the effect size varies across studies.
(JPG)

## References

[1] Hartvigsen J, Hancock MJ, Kongsted A, Louw Q, Ferreira ML, Genevay S, et al. What low back pain is and why we need to pay attention. Lancet. 2018; 391(10137): 2356–2367. doi: 10.1016/S0140-6736(18)30480-X 29573870

[2] Vos T, Abajobir AA, Abate KH, Abbafati C, Abbas KM, Abd-Allah F, et al. Global, regional, and national incidence, prevalence, and years lived with disability for 328 diseases and injuries for 195 countries, 1990–2016: a systematic analysis for the Global Burden of Disease Study 2016. Lancet. 2017; 390(10100): 1211–1259. doi: 10.1016/S0140-6736(17)32154-2 28919117 PMC5605509

[3] James SL, Abate D, Abate KH, Abay SM, Abbafati C, Abbasi N, et al. Global, regional, and national incidence, prevalence, and years lived with disability for 354 diseases and injuries for 195 countries and territories, 1990–2017: a systematic analysis for the Global Burden of Disease Study 2017. Lancet. 2018; 392(10159): 1789–1858. doi: 10.1016/S0140-6736(18)32279-7 30496104 PMC6227754

[4] Ferreira ML, de Luca K, Haile LM, Steinmetz JD, Culbreth GT, Cross M, et al. Global, regional, and national burden of low back pain, 1990–2020, its attributable risk factors, and projections to 2050: a systematic analysis of the Global Burden of Disease Study 2021. Lancet Rheumatol. 2023; 5(6): 316–329. doi: 10.1016/S2665-9913(23)00098-X 37273833 PMC10234592

[5] Hestbaek L, Leboeuf-Yde C, Manniche C. Low back pain: what is the long-term course? A review of studies of general patient populations. Eur Spine J. 2003; 12(2): 149–165. doi: 10.1007/s00586-002-0508-5 12709853 PMC3784852

[6] Leboeuf-Yde C, Grønstvedt A, Borge JA, Lothe J, Magnesen E, Nilsson Ø, et al. The Nordic back pain subpopulation program: a 1-year prospective multicenter study of outcomes of persistent low-back pain in chiropractic patients. J Manipulative Physiol Ther. 2005; 28(2): 90–96. doi: 10.1016/j.jmpt.2005.01.010 15800507

[7] Klenerman L, Slade P, Stanley I, Pennie B, Reilly J, Atchison L, et al. The prediction of chronicity in patients with an acute attack of low back pain in a general practice setting. Spine. 1995; 20(4): 478–484. doi: 10.1097/00007632-199502001-00012 7747233

[8] Patrick N, Emanski E, Knaub MA. Acute and chronic low back pain. Med Clin North Am. 2014; 98(4): 777–789. doi: 10.1016/j.mcna.2014.03.005 24994051

[9] Ebenbichler GR, Oddsson LI, Kollmitzer J, Erim Z. Sensory-motor control of the lower back: implications for rehabilitation. Med Sci Sports Exerc. 2001; 33(11): 1889–1898. doi: 10.1097/00005768-200111000-00014 11689740

[10] Hodges PW, Tucker K. Moving differently in pain: a new theory to explain the adaptation to pain. Pain. 2011; 152(3): 90–98. 10.1016/j.pain.2010.10.020 21087823

[11] Hodges PW, Danneels L. Changes in structure and function of the back muscles in low back pain: different time points, observations, and mechanisms. J Orthop Sports Phys Ther. 2019; 49(6): 464–476. doi: 10.2519/jospt.2019.8827 31151377

[12] Winter DA, Patla AE, Frank JS. Assessment of balance control in humans. Med Prog Technol. 1990; 16(1–2): 31–51. 2138696

[13] Horak FB. Postural orientation and equilibrium: what do we need to know about neural control of balance to prevent falls? Age Ageing. 2006; 35(suppl_2): ii7–ii11. doi: 10.1093/ageing/afl077 16926210

[14] Horak FB. Clinical measurement of postural control in adults. Phys Ther. 1987; 67(12): 1881–1885. doi: 10.1093/ptj/67.12.1881 3685116

[15] Massion J. Postural control system. Curr Opin Neurobiol. 1994; 4(6): 877–887. doi: 10.1016/0959-4388(94)90137-6 7888772

[16] Nielsen JB. Human spinal motor control. Annu Rev Neurosci. 2016; 39: 81–101. doi: 10.1146/annurev-neuro-070815-013913 27023730

[17] Winter DA. Human balance and posture control during standing and walking. Gait Posture. 1995; 3(4): 193–214.

[18] Allum J, Bloem B, Carpenter M, Hulliger M, Hadders-Algra M. Proprioceptive control of posture: a review of new concepts. Gait Posture. 1998; 8(3): 214–242. doi: 10.1016/s0966-6362(98)00027-7 10200410

[19] Sung W, Abraham M, Plastaras C, Silfies SP. Trunk motor control deficits in acute and subacute low back pain are not associated with pain or fear of movement. Spine J. 2015; 15(8): 1772–1782. doi: 10.1016/j.spinee.2015.04.010 25862508 PMC4516579

[20] Poppele R, Bosco G. Sophisticated spinal contributions to motor control. Trends Neurosci. 2003; 26(5): 269–276. doi: 10.1016/S0166-2236(03)00073-0 12744844

[21] Cholewicki J, Polzhofer GK, Radebold A. Postural control of trunk during unstable sitting. J Biomech. 2000; 33(12): 1733–1737. doi: 10.1016/s0021-9290(00)00126-3 11006402

[22] Radebold A, Cholewicki J, Polzhofer GK, Greene HS. Impaired postural control of the lumbar spine is associated with delayed muscle response times in patients with chronic idiopathic low back pain. Spine. 2001; 26(7): 724–730. doi: 10.1097/00007632-200104010-00004 11295888

[23] Slota GP, Granata KP, Madigan ML. Effects of seated whole-body vibration on postural control of the trunk during unstable seated balance. Clin Biomech. 2008; 23(4): 381–386. doi: 10.1016/j.clinbiomech.2007.11.006 18093708

[24] Larivière C, Mecheri H, Shahvarpour A, Gagnon D, Shirazi-Adl A. Criterion validity and between-day reliability of an inertial-sensor-based trunk postural stability test during unstable sitting. J Electromyogr Kinesiol. 2013; 23(4): 899–907. doi: 10.1016/j.jelekin.2013.03.002 23582401

[25] Roberts BWR, Vette AH. A kinematics recommendation for trunk stability and control assessments during unstable sitting. Med Eng Phys. 2019; 73: 73–76. doi: 10.1016/j.medengphy.2019.08.004 31495723

[26] Alshehri MA, van den Hoorn W, Klyne DM, Hodges PW. Coordination of hip and spine to maintain equilibrium in unstable sitting revealed by spectral analysis. J Neurophysiol. 2021; 125(5): 1814–1824. doi: 10.1152/jn.00555.2020 33826432

[27] Roberts BWR, Gholibeigian F, Lewicke J, Vette AH. Spatial and temporal relation of kinematics and muscle activity during unstable sitting. J Electromyogr Kinesiol. 2020; 52: 102418. doi: 10.1016/j.jelekin.2020.102418 32298966

[28] Barbado D, Moreside J, Vera-Garcia FJ. Reliability and repetition effect of the center of pressure and kinematics parameters that characterize trunk postural control during unstable sitting test. PM&R. 2017; 9(3): 219–230. doi: 10.1016/j.pmrj.2016.08.029 27616542

[29] Shahvarpour A, Gagnon D, Preuss R, Henry SM, Larivière C. Trunk postural balance and low back pain: reliability and relationship with clinical changes following a lumbar stabilization exercise program. Gait Posture. 2018; 61: 375–381. doi: 10.1016/j.gaitpost.2018.02.006 29448220

[30] Cyr KM, Wilson SE, Mehyar F, Sharma NK. Trunk control response to unstable seated posture during various feedback conditions in people with chronic low back pain. J Allied Health. 2019; 48(1): 54–60. 30826831

[31] Willigenburg NW, Kingma I, van Dieën JH. Center of pressure trajectories, trunk kinematics and trunk muscle activation during unstable sitting in low back pain patients. Gait Posture. 2013; 38(4): 625–630. doi: 10.1016/j.gaitpost.2013.02.010 23473809

[32] Shahvarpour A, Preuss R, Larivière C. The effect of extensible and non-extensible lumbar belts on trunk postural balance in subjects with low back pain and healthy controls. Gait Posture. 2019; 72: 211–216. doi: 10.1016/j.gaitpost.2019.06.013 31255888

[33] Van Dieën JH, Koppes LLJ, Twisk JWR. Low back pain history and postural sway in unstable sitting. Spine. 2010; 35(7): 812–817. doi: 10.1097/BRS.0b013e3181bb81a8 20195213

[34] Larivière C, Gagnon DH, Mecheri H. Trunk postural control in unstable sitting: effect of sex and low back pain status. Clin Biomech. 2015; 30(9): 933–939. doi: 10.1016/j.clinbiomech.2015.07.006 26253690

[35] van den Hoorn W, Meroni R, Klyne D, Alshehri MA, Hodges PW. Balance control in unstable sitting in individuals with an acute episode of low back pain. Gait Posture. 2022; 95: 15–21. doi: 10.1016/j.gaitpost.2022.03.014 35398705

[36] Simmonds M, Higgins J. Covariate heterogeneity in meta‐analysis: criteria for deciding between meta‐regression and individual patient data. Stat Med. 2007; 26(15): 2982–2999. doi: 10.1002/sim.2768 17195960

[37] Kontopantelis E. A comparison of one‐stage vs two‐stage individual patient data meta‐analysis methods: a simulation study. Res Synth Methods. 2018; 9(3): 417–430. doi: 10.1002/jrsm.1303 29786975 PMC6175226

[38] Burke DL, Ensor J, Riley RD. Meta‐analysis using individual participant data: one‐stage and two‐stage approaches, and why they may differ. Stat Med. 2017; 36(5): 855–875. doi: 10.1002/sim.7141 27747915 PMC5297998

[39] Riley RD, Lambert PC, Abo-Zaid G. Meta-analysis of individual participant data: rationale, conduct, and reporting. BMJ. 2010; 340: c221. doi: 10.1136/bmj.c221 20139215

[40] Debray TP, Moons KG, van Valkenhoef G, Efthimiou O, Hummel N, Groenwold RH, et al. Get real in individual participant data (IPD) meta‐analysis: a review of the methodology. Res Synth Methods. 2015; 6(4): 293–309. doi: 10.1002/jrsm.1160 26287812 PMC5042043

[41] Knox MF, Chipchase LS, Schabrun SM, Romero RJ, Marshall PW. Anticipatory and compensatory postural adjustments in people with low back pain: a systematic review and meta-analysis. Spine J. 2018; 18(10): 1934–1949. doi: 10.1016/j.spinee.2018.06.008 29906616

[42] Prins MR, Griffioen M, Veeger TT, Kiers H, Meijer OG, van der Wurff P, et al. Evidence of splinting in low back pain? A systematic review of perturbation studies. Eur Spine J. 2018; 27(1): 40–59. doi: 10.1007/s00586-017-5287-0 28900711

[43] Maaswinkel E, Griffioen M, Perez R, van Dieën JH. Methods for assessment of trunk stabilization, a systematic review. J Electromyogr Kinesiol. 2016; 26: 18–35. doi: 10.1016/j.jelekin.2015.12.010 26803526

[44] Azadinia F, Kingma I, Mazaheri M. Effect of external lumbar supports on joint position sense, postural control, and postural adjustment: a systematic review. Disabil Rehabil. 2023; 45(5): 753–771. doi: 10.1080/09638288.2022.2043464 35259058

[45] Ruhe A, Fejer R, Walker B. The test–retest reliability of centre of pressure measures in bipedal static task conditions–a systematic review of the literature. Gait Posture. 2010; 32(4): 436–445. doi: 10.1016/j.gaitpost.2010.09.012 20947353

[46] Mazaheri M, Coenen P, Parnianpour M, Kiers H, van Dieën JH. Low back pain and postural sway during quiet standing with and without sensory manipulation: a systematic review. Gait Posture. 2013; 37(1): 12–22. doi: 10.1016/j.gaitpost.2012.06.013 22796243

[47] Berenshteyn Y, Gibson K, Hackett GC, Trem AB, Wilhelm M. Is standing balance altered in individuals with chronic low back pain? A systematic review. Disabil Rehabil. 2019; 41(13): 1514–1523. doi: 10.1080/09638288.2018.1433240 29382241

[48] Ruhe A, Fejer R, Walker B. Center of pressure excursion as a measure of balance performance in patients with non-specific low back pain compared to healthy controls: a systematic review of the literature. Eur Spine J. 2011; 20(3): 358–368. doi: 10.1007/s00586-010-1543-2 20721676 PMC3048236

[49] Koch C, Hänsel F. Non-specific low back pain and postural control during quiet standing—a systematic review. Front Psychol. 2019; 10: 586. doi: 10.3389/fpsyg.2019.00586 30967811 PMC6440285

[50] Shanbehzadeh S, ShahAli S, Ebrahimi Takamjani I, Vlaeyen JW, Salehi R, Jafari H. Association of pain-related threat beliefs and disability with postural control and trunk motion in individuals with low back pain: a systematic review and meta-analysis. Eur Spine J. 2022; 31: 1802–1820. doi: 10.1007/s00586-022-07261-4 35583666

[51] Ge L, Wang C, Zhou H, Yu Q, Li X. Effects of low back pain on balance performance in elderly people: a systematic review and meta-analysis. Eur Rev Aging Phys Act. 2021; 18(1): 1–10. 34090345 10.1186/s11556-021-00263-z PMC8180028

[52] Clark RA, Mentiplay BF, Pua Y-H, Bower KJ. Reliability and validity of the Wii Balance Board for assessment of standing balance: a systematic review. Gait Posture. 2018; 61: 40–54. doi: 10.1016/j.gaitpost.2017.12.022 29304510

[53] Park J, Nguyen VQ, Ho RL, Coombes SA. The effect of chronic low back pain on postural control during quiet standing: a meta-analysis. Sci Rep. 2023; 13(1): 7928. doi: 10.1038/s41598-023-34692-w 37193730 PMC10188550

[54] Dal Farra F, Arippa F, Mauro A, Cocco M, Porcu E, Tramontano M, et al. Effects of exercise on balance in patients with non-specific low back pain: a systematic review and meta-analysis. Eur J Phys Rehabil Med. 2022; 58(3): 423. doi: 10.23736/S1973-9087.21.07293-2 34636528 PMC9980551

[55] Mehdizadeh S, Van Ooteghem K, Gulka H, Nabavi H, Faieghi M, Taati B, et al. A systematic review of center of pressure measures to quantify gait changes in older adults. Exp Gerontol. 2021; 143: 111170. doi: 10.1016/j.exger.2020.111170 33238173

[56] Stewart LA, Clarke M, Rovers M, Riley RD, Simmonds M, Stewart G, et al. Preferred reporting items for a systematic review and meta-analysis of individual participant data: the PRISMA-IPD statement. JAMA. 2015; 313(16): 1657–1665. 25919529 10.1001/jama.2015.3656

[57] Alshehri MA, van den Hoorn W, Klyne DM, Hodges PW. Postural control of the trunk in individuals with and without low back pain during unstable sitting: a protocol for a systematic review with an individual participant data meta-analysis. PLoS One. 2022; 17(5): e0268381. doi: 10.1371/journal.pone.0268381 35551559 PMC9098032

[58] Paillard T, Noé F. Techniques and methods for testing the postural function in healthy and pathological subjects. BioMed Res Int. 2015; 2015: 891390. doi: 10.1155/2015/891390 26640800 PMC4659957

[59] Viera AJ, Garrett JM. Understanding interobserver agreement: the kappa statistic. Fam Med. 2005; 37(5): 360–363. 15883903

[60] Partlett C, Riley RD. Random effects meta‐analysis: coverage performance of 95% confidence and prediction intervals following REML estimation. Stat Med. 2017; 36(2): 301–317. doi: 10.1002/sim.7140 27714841 PMC5157768

[61] Abo-Zaid G, Guo B, Deeks JJ, Debray TP, Steyerberg EW, Moons KG, et al. Individual participant data meta-analyses should not ignore clustering. J Clin Epidemiol. 2013; 66(8): 865–873. doi: 10.1016/j.jclinepi.2012.12.017 23651765 PMC3717206

[62] Takeshima N, Sozu T, Tajika A, Ogawa Y, Hayasaka Y, Furukawa TA. Which is more generalizable, powerful and interpretable in meta-analyses, mean difference or standardized mean difference? BMC Med Res Methodol. 2014; 14(1): 1–7. doi: 10.1186/1471-2288-14-30 24559167 PMC3936842

[63] Andrade C. Mean difference, standardized mean difference (SMD), and their use in meta-analysis: as simple as it gets. J Clin Psychiatry. 2020; 81(5): 11349. doi: 10.4088/JCP.20f13681 32965803

[64] Higgins JP, Thompson SG, Deeks JJ, Altman DG. Measuring inconsistency in meta-analyses. BMJ. 2003; 327(7414): 557–560. doi: 10.1136/bmj.327.7414.557 12958120 PMC192859

[65] Higgins JP. Commentary: heterogeneity in meta-analysis should be expected and appropriately quantified. Int J Epidemiol. 2008; 37(5): 1158–1160. doi: 10.1093/ije/dyn204 18832388

[66] Rücker G, Schwarzer G, Carpenter JR, Schumacher M. Undue reliance on I2 in assessing heterogeneity may mislead. BMC Med Res Methodol. 2008; 8: 1–9. 19036172 10.1186/1471-2288-8-79 PMC2648991

[67] Riley RD, Higgins JP, Deeks JJ. Interpretation of random effects meta-analyses. BMJ. 2011; 342: d549. doi: 10.1136/bmj.d549 21310794

[68] Guddat C, Grouven U, Bender R, Skipka G. A note on the graphical presentation of prediction intervals in random-effects meta-analyses. Syst Rev. 2012; 1: 1–5. 22839660 10.1186/2046-4053-1-34 PMC3552946

[69] IntHout J, Ioannidis JP, Rovers MM, Goeman JJ. Plea for routinely presenting prediction intervals in meta-analysis. BMJ Open. 2016; 6(7): e010247. doi: 10.1136/bmjopen-2015-010247 27406637 PMC4947751

[70] Borenstein M. In a meta-analysis, the I-squared statistic does not tell us how much the effect size varies. J Clin Epidemiol. 2022; 152: 281–284. doi: 10.1016/j.jclinepi.2022.10.003 36223816

[71] Borenstein M, Higgins JP, Hedges LV, Rothstein HR. Basics of meta‐analysis: I^2^ is not an absolute measure of heterogeneity. Res Synth Methods. 2017; 8(1): 5–18. 28058794 10.1002/jrsm.1230

[72] Navalgund AR. Evaluating the effect of a 10-week stabilization exercise program on the postural stability and the neuromuscular control of the spine in subjects with subacute recurrent low back pain. Ohio: Ohio State University; 2009.

[73] Reeves NP, Cholewicki J, Lee AS, Mysliwiec LW. The effects of stochastic resonance stimulation on spine proprioception and postural control in chronic low back pain patients. Spine. 2009; 34(4): 316–321. doi: 10.1097/BRS.0b013e3181971e09 19214090

[74] van Dieën JH, Koppes LLJ, Twisk JWR. Postural sway parameters in seated balancing; their reliability and relationship with balancing performance. Gait Posture. 2010; 31(1): 42–46. doi: 10.1016/j.gaitpost.2009.08.242 19783440

[75] Shahvarpour A, Shirazi-Adl A, Larivière C. Active-passive biodynamics of the human trunk when seated on a wobble chair. J Biomech. 2016; 49(6): 939–945. doi: 10.1016/j.jbiomech.2016.01.042 26897647

[76] Larivière C, Preuss R, Ludvig D, Henry SM. Is postural control during unstable sitting a proxy measure for determinants associated with lumbar stability? J Biomech. 2020; 102: 109581. doi: 10.1016/j.jbiomech.2019.109581 31902612

[77] Silfies SP, Cholewicki J, Radebold A. The effects of visual input on postural control of the lumbar spine in unstable sitting. Hum Mov Sci. 2003; 22(3): 237–252. doi: 10.1016/s0167-9457(03)00046-0 12967756

[78] Reeves NP, Everding VQ, Cholewicki J, Morrisette DC. The effects of trunk stiffness on postural control during unstable seated balance. Exp Brain Res. 2006; 174(4): 694–700. doi: 10.1007/s00221-006-0516-5 16724177

[79] van der Burg JCE, van Wegen EEH, Rietberg MB, Kwakkel G, van Dieën JH. Postural control of the trunk during unstable sitting in Parkinson’s disease. Parkinsonism Relat Disord. 2006; 12(8): 492–498. doi: 10.1016/j.parkreldis.2006.06.007 16934518

[80] Cholewicki J, Peter Reeves N, Everding VQ, Morrisette DC. Lumbosacral orthoses reduce trunk muscle activity in a postural control task. J Biomech. 2007; 40(8): 1731–1736. doi: 10.1016/j.jbiomech.2006.08.005 17054963

[81] Lee H, Granata KP. Process stationarity and reliability of trunk postural stability. Clin Biomech. 2008; 23(6): 735–742. doi: 10.1016/j.clinbiomech.2008.01.008 18304711 PMC2832481

[82] Lee H, Granata KP, Madigan ML. Effects of trunk exertion force and direction on postural control of the trunk during unstable sitting. Clin Biomech. 2008; 23(5): 505–509. doi: 10.1016/j.clinbiomech.2008.01.003 18282647

[83] Hendershot BD, Nussbaum MA. Persons with lower-limb amputation have impaired trunk postural control while maintaining seated balance. Gait Posture. 2013; 38(3): 438–442. doi: 10.1016/j.gaitpost.2013.01.008 23391751

[84] Hendershot BD, Toosizadeh N, Muslim K, Madigan ML, Nussbaum MA. Evidence for an exposure-response relationship between trunk flexion and impairments in trunk postural control. J Biomech. 2013; 46(14): 2554–2557. doi: 10.1016/j.jbiomech.2013.07.021 23932325

[85] Barbado D, Barbado LC, Elvira JLL, Dieën JHv, Vera-Garcia FJ. Sports-related testing protocols are required to reveal trunk stability adaptations in high-level athletes. Gait Posture. 2016; 49: 90–96. doi: 10.1016/j.gaitpost.2016.06.027 27395448

[86] Barbado D, Lopez-Valenciano A, Juan-Recio C, Montero-Carretero C, van Dieën JH, Vera-Garcia FJ. Trunk stability, trunk strength and sport performance level in Judo. PLoS One. 2016; 11(5): e0156267. doi: 10.1371/journal.pone.0156267 27232602 PMC4883759

[87] Beaudette SM, Larson KJ, Larson DJ, Brown SHM. Low back skin sensitivity has minimal impact on active lumbar spine proprioception and stability in healthy adults. Exp Brain Res. 2016; 234(8): 2215–2226. doi: 10.1007/s00221-016-4625-5 27010722

[88] Ruggiero SA, Frost LR, Vallis LA, Brown SHM. Effect of short-term application of kinesio tape on the flexion-relaxation phenomenon, trunk postural control and trunk repositioning in healthy females. J Sports Sci. 2016; 34(9): 862–870. doi: 10.1080/02640414.2015.1076164 26252507

[89] Barbado Murillo D, Caballero Sánchez C, Moreside J, Vera-García FJ, Moreno FJ. Can the structure of motor variability predict learning rate? J Exp Psychol Hum Percept Perform. 2017; 43(3): 596–607. doi: 10.1037/xhp0000303 28095006

[90] Glofcheskie GO, Brown SHM. Athletic background is related to superior trunk proprioceptive ability, postural control, and neuromuscular responses to sudden perturbations. Hum Mov Sci. 2017; 52: 74–83. doi: 10.1016/j.humov.2017.01.009 28135584

[91] Acasio JC, Butowicz CM, Golyski PR, Nussbaum MA, Hendershot BD. Associations between trunk postural control in walking and unstable sitting at various levels of task demand. J Biomech. 2018; 75: 181–185. doi: 10.1016/j.jbiomech.2018.05.006 29792285

[92] Williams AD, Boser QA, Kumawat AS, Agarwal K, Rouhani H, Vette AH. Design and evaluation of an instrumented wobble board for assessing and training dynamic seated balance. J Biomech Eng. 2018; 140(4): 041006. doi: 10.1115/1.4038747 29238816

[93] Barbado D, Reina R, Roldan A, McCulloch K, Campayo-Piernas M, Vera-Garcia FJ. How much trunk control is affected in adults with moderate-to-severe cerebral palsy? J Biomech. 2019; 82: 368–874. doi: 10.1016/j.jbiomech.2018.11.009 30473138

[94] Acasio JC, Nussbaum MA, Hendershot BD. Trunk-pelvic coordination during unstable sitting with varying task demand: a methodological study. J Biomech. 2021; 118: 110299. doi: 10.1016/j.jbiomech.2021.110299 33581439

[95] Rogério de Oliveira M, Fabrin LF, Wilson de Oliveira Gil A, Benassi GH, Camargo MZ, Alexandre da Silva R, et al. Acute effect of core stability and sensory-motor exercises on postural control during sitting and standing positions in young adults. J Bodyw Mov Ther. 2021; 28: 98–103. doi: 10.1016/j.jbmt.2021.07.021 34776207

[96] Reeves NP, Ramadan A, Popovich JM Jr, Prokop LL, Zatkin MA, DeStefano LA, et al. Stability threshold during seated balancing is sensitive to low back pain and safe to assess. J Biomech. 2021; 125: 110541. doi: 10.1016/j.jbiomech.2021.110541 34198020

[97] Tanaka ML, Nussbaum MA, Ross SD. Evaluation of the threshold of stability for the human spine. J Biomech. 2009; 42(8): 1017–1022. doi: 10.1016/j.jbiomech.2009.02.008 19345355 PMC2683902

[98] Collins JJ, De Luca CJ. Open-loop and closed-loop control of posture: a random-walk analysis of center-of-pressure trajectories. Exp Brain Res. 1993; 95: 308–318. doi: 10.1007/BF00229788 8224055

[99] Collins J, De Luca C. Upright, correlated random walks: a statistical‐biomechanics approach to the human postural control system. Chaos. 1995; 5(1): 57–63. doi: 10.1063/1.166086 12780156

[100] De Luca JC-C. The effects of visual input on open-loop and closed-loop postural control mechanisms. Exp Brain Res. 1995; 103: 151–163. doi: 10.1007/BF00241972 7615030

[101] Reeves NP, Cholewicki J, Narendra KS. Effects of reflex delays on postural control during unstable seated balance. J Biomech. 2009; 42(2): 164–170. doi: 10.1016/j.jbiomech.2008.10.016 19121523 PMC5571836

[102] Willigenburg NW, Kingma I, van Dieën JH. Precision control of an upright trunk posture in low back pain patients. Clin Biomech. 2012; 27(9): 866–871. doi: 10.1016/j.clinbiomech.2012.06.002 22748373

[103] Collins J, De Luca C, Burrows A, Lipsitz L. Age-related changes in open-loop and closed-loop postural control mechanisms. Exp Brain Res. 1995; 104: 480–492. doi: 10.1007/BF00231982 7589299

[104] van Dieën JH, Selen LP, Cholewicki J. Trunk muscle activation in low-back pain patients, an analysis of the literature. J Electromyogr Kinesiol. 2003; 13(4): 333–351. doi: 10.1016/s1050-6411(03)00041-5 12832164

[105] Radebold A, Cholewicki J, Panjabi MM, Patel TC. Muscle response pattern to sudden trunk loading in healthy individuals and in patients with chronic low back pain. Spine. 2000; 25(8): 947–954. doi: 10.1097/00007632-200004150-00009 10767807

[106] Tong MH, Mousavi SJ, Kiers H, Ferreira P, Refshauge K, van Dieën J. Is there a relationship between lumbar proprioception and low back pain? A systematic review with meta-analysis. Arch Phys Med Rehabil. 2017; 98(1): 120–136. doi: 10.1016/j.apmr.2016.05.016 27317866

[107] Lee AS, Cholewicki J, Reeves NP, Zazulak BT, Mysliwiec LW. Comparison of trunk proprioception between patients with low back pain and healthy controls. Arch Phys Med Rehabil. 2010; 91(9): 1327–1331. doi: 10.1016/j.apmr.2010.06.004 20801248 PMC4896302

[108] Parkhurst TM, Burnett CN. Injury and proprioception in the lower back. J Orthop Sports Phys Ther. 1994; 19(5): 282–295. doi: 10.2519/jospt.1994.19.5.282 8199622

[109] Korakakis V, O’Sullivan K, Kotsifaki A, Sotiralis Y, Giakas G. Lumbo-pelvic proprioception in sitting is impaired in subgroups of low back pain–but the clinical utility of the differences is unclear. A systematic review and meta-analysis. PLoS One. 2021; 16(4): e0250673. doi: 10.1371/journal.pone.0250673 33901255 PMC8075231

[110] Freddolini M, Strike S, Lee RYW. The role of trunk muscles in sitting balance control in people with low back pain. J Electromyogr Kinesiol. 2014; 24(6): 947–953. doi: 10.1016/j.jelekin.2014.09.009 25287529

[111] Van Daele U, Hagman F, Truijen S, Vorlat P, Van Gheluwe B, Vaes P. Differences in balance strategies between nonspecific chronic low back pain patients and healthy control subjects during unstable sitting. Spine. 2009; 34(11): 1233–1238. doi: 10.1097/BRS.0b013e31819ca3ee 19444072

[112] Hodges P, van den Hoorn W, Dawson A, Cholewicki J. Changes in the mechanical properties of the trunk in low back pain may be associated with recurrence. J Biomech. 2009; 42(1): 61–66. doi: 10.1016/j.jbiomech.2008.10.001 19062020

[113] Navalgund A, Buford JA, Briggs MS, Givens DL. Trunk muscle reflex amplitudes increased in patients with subacute, recurrent LBP treated with a 10-week stabilization exercise program. Motor Control. 2013; 17(1): 1–17. doi: 10.1123/mcj.17.1.1 22964879 PMC3881973

[114] Liebetrau A, Puta C, Anders C, de Lussanet MH, Wagner H. Influence of delayed muscle reflexes on spinal stability: model-based predictions allow alternative interpretations of experimental data. Hum Mov Sci. 2013; 32(5): 954–970. doi: 10.1016/j.humov.2013.03.006 23915574

[115] Shenoy S, Balachander H, Sandhu J. Long latency reflex response of superficial trunk musculature in athletes with chronic low back pain. J Back Musculoskelet Rehabil. 2013; 26(4): 445–450. doi: 10.3233/BMR-130404 23948831

[116] Cholewicki J, Silfies SP, Shah RA, Greene HS, Reeves NP, Alvi K, et al. Delayed trunk muscle reflex responses increase the risk of low back injuries. Spine. 2005; 30(23): 2614–2620. doi: 10.1097/01.brs.0000188273.27463.bc 16319747

[117] Vera-Garcia FJ, Brown SHM, Gray JR, McGill SM. Effects of different levels of torso coactivation on trunk muscular and kinematic responses to posteriorly applied sudden loads. Clin Biomech. 2006; 21(5): 443–455. doi: 10.1016/j.clinbiomech.2005.12.006 16442677

[118] Marras WS, Davis KG, Ferguson SA, Lucas BR, Gupta P. Spine loading characteristics of patients with low back pain compared with asymptomatic individuals. Spine. 2001; 26(23): 2566–2574. doi: 10.1097/00007632-200112010-00009 11725237

[119] Peterka RJ. Sensorimotor integration in human postural control. J Neurophysiol. 2002; 88(3): 1097–1118. doi: 10.1152/jn.2002.88.3.1097 12205132

[120] Lee WA. A control systems framework for understanding normal and abnormal posture. Am J Occup Ther. 1989; 43(5): 291–301. doi: 10.5014/ajot.43.5.291 2655455

[121] Ivanenko Y, Gurfinkel VS. Human postural control. Front Neurosci. 2018; 12: 171. doi: 10.3389/fnins.2018.00171 29615859 PMC5869197

[122] Akpunarli B, Yilgor C, Alanay A. Proprioception after spine injury and surgery. In: Kaya D, Yosmaoglu B, Doral MN, editors. Proprioception in orthopaedics, sports medicine and rehabilitation. Cham, Switzerland: Springer international publishing AG; 2018. pp. 65–71.

[123] Peterka RJ. Postural control model interpretation of stabilogram diffusion analysis. Biol Cybern. 2000; 82(4): 335–343. doi: 10.1007/s004220050587 10804065

[124] Oomen NM, Reeves NP, Priess MC, van Dieën JH. Trunk muscle coactivation is tuned to changes in task dynamics to improve responsiveness in a seated balance task. J Electromyogr Kinesiol. 2015; 25(5): 765–772. doi: 10.1016/j.jelekin.2015.07.001 26216868

[125] Fielding RA, Vellas B, Evans WJ, Bhasin S, Morley JE, Newman AB, et al. Sarcopenia: an undiagnosed condition in older adults. Current consensus definition: prevalence, etiology, and consequences. International working group on sarcopenia. J Am Med Dir Assoc. 2011; 12(4): 249–256. doi: 10.1016/j.jamda.2011.01.003 21527165 PMC3377163

[126] Faulkner JA, Larkin LM, Claflin DR, Brooks SV. Age-related changes in the structure and function of skeletal muscles. Clin Exp Pharmacol Physiol. 2007; 34(11): 1091–1096. doi: 10.1111/j.1440-1681.2007.04752.x 17880359

[127] Ribeiro F, Oliveira J. Aging effects on joint proprioception: the role of physical activity in proprioception preservation. Eur Rev Aging Phys Act. 2007; 4(2): 71–76.

[128] Mignardot J-B, Olivier I, Promayon E, Nougier V. Obesity impact on the attentional cost for controlling posture. PLoS One. 2010; 5(12): e14387. doi: 10.1371/journal.pone.0014387 21187914 PMC3004786

[129] Del Porto H, Pechak C, Smith D, Reed-Jones R. Biomechanical effects of obesity on balance. Int J Exerc Sci. 2012; 5(4): 301–320.

[130] Linton SJ, Shaw WS. Impact of psychological factors in the experience of pain. Phys Ther. 2011; 91(5): 700–711. doi: 10.2522/ptj.20100330 21451097

[131] Sterne JA, Sutton AJ, Ioannidis JP, Terrin N, Jones DR, Lau J, et al. Recommendations for examining and interpreting funnel plot asymmetry in meta-analyses of randomised controlled trials. BMJ. 2011; 343: d4002. doi: 10.1136/bmj.d4002 21784880

[132] Schielzeth H, Dingemanse NJ, Nakagawa S, Westneat DF, Allegue H, Teplitsky C, et al. Robustness of linear mixed‐effects models to violations of distributional assumptions. Methods Ecol Evol. 2020; 11(9): 1141–1152.

