# Supplementary material for: Trunk postural control during unstable sitting among individuals with and without low back pain: A systematic review with an individual participant data meta-analysis
Source: PLoS One. 2024 Jan 24;19(1):e0296968. doi: 10.1371/journal.pone.0296968 (PMC10807788; doi:10.1371/journal.pone.0296968)
Supplement: S2 Table — (DOCX) [file pone.0296968.s003.docx]

| **Table S2.** Search strategy used in Embase database | | |
| --- | --- | --- |
| **#** | **Query** | **Results** |
| S4 | #1 AND #2 AND #3 | 3,319 |
| S3 | (sit:ab,ti OR sitting:ab,ti OR 'unstable sitting':ab,ti OR seat:ab,ti OR 'unstable seat':ab,ti OR seated:ab,ti OR 'unstable seated':ab,ti OR chair:ab,ti OR 'wobble chair':ab,ti OR 'unstable chair':ab,ti) AND ([adult]/lim OR [young adult]/lim OR [middle aged]/lim OR [aged]/lim OR [very elderly]/lim) AND [humans]/lim AND [english]/lim AND [embase]/lim | 34,732 |
| S2 | (balance:ab,ti OR 'balance control':ab,ti OR 'postural balance':ab,ti OR 'postural control':ab,ti OR stability:ab,ti OR 'postural stability':ab,ti OR 'trunk stability':ab,ti OR 'spine stability':ab,ti OR 'motor control':ab,ti OR 'trunk control':ab,ti OR 'spine control':ab,ti OR 'postural sway':ab,ti OR equilibrium:ab,ti OR kinematics:ab,ti OR 'cent* of pressure':ab,ti OR cop:ab,ti) AND ([adult]/lim OR [young adult]/lim OR [middle aged]/lim OR [aged]/lim OR [very elderly]/lim) AND [humans]/lim AND [english]/lim AND [embase]/lim | 144,436 |
| S1 | ('low back pain':ab,ti OR 'lower back pain':ab,ti OR 'back pain':ab,ti OR lbp:ab,ti OR clbp:ab,ti OR nslbp:ab,ti OR 'low back ache':ab,ti OR 'lower back ache':ab,ti OR 'back ache':ab,ti OR 'backache':ab,ti OR 'low back injury':ab,ti OR 'lower back injury':ab,ti OR 'back injury':ab,ti OR 'lumbar pain':ab,ti OR lumbago:ab,ti OR 'healthy':ab,ti OR 'pain-free':ab,ti OR 'symptom-free':ab,ti OR 'without pain':ab,ti OR 'subjects':ab,ti OR 'participants':ab,ti OR 'adults':ab,ti OR 'individuals':ab,ti OR 'volunteers':ab,ti) AND ([adult]/lim OR [young adult]/lim OR [middle aged]/lim OR [aged]/lim OR [very elderly]/lim) AND [humans]/lim AND [english]/lim AND [embase]/lim | 2,133,775 |
| ***Interface:*** Elsevier.  ***Search Screen:*** Advanced Search.  ***Database:*** Embase.  ***Searching Date:*** 25 March 2022. | | |
