# Supplementary material for: Trunk postural control during unstable sitting among individuals with and without low back pain: A systematic review with an individual participant data meta-analysis
Source: PLoS One. 2024 Jan 24;19(1):e0296968. doi: 10.1371/journal.pone.0296968 (PMC10807788; doi:10.1371/journal.pone.0296968)
Supplement: S3 Table — (DOCX) [file pone.0296968.s004.docx]

| **Table S3.** Search strategy used in Scopus database | | |
| --- | --- | --- |
| **#** | **Query** | **Results** |
| S4 | #1 AND #2 AND #3 | 3,952 |
| S3 | TITLE-ABS ("sit" OR "sitting" OR "unstable sitting" OR "seat" OR "unstable seat" OR "seated" OR "unstable seated" OR "chair" OR "wobble chair" OR "unstable chair") AND (LIMIT-TO (LANGUAGE, "English")) AND (LIMIT-TO (EXACTKEYWORD, "Human") OR LIMIT-TO (EXACTKEYWORD, "Humans") OR LIMIT-TO (EXACTKEYWORD, "Adult")) | 58,101 |
| S2 | TITLE-ABS ("balance" OR "balance control" OR "postural balance" OR "postural control" OR "stability" OR "postural stability" OR "trunk stability" OR "spine stability" OR "motor control" OR "trunk control" OR "spine control" OR "postural sway" OR "equilibrium" OR "kinematics" OR "cent* of pressure" OR "CoP") AND (LIMIT-TO (LANGUAGE, "English")) AND (LIMIT-TO (EXACTKEYWORD, "Human") OR LIMIT-TO (EXACTKEYWORD, "Humans") OR LIMIT-TO (EXACTKEYWORD, "Adult")) | 404,976 |
| S1 | TITLE-ABS ("low back pain" OR "lower back pain" OR "back pain" OR "LBP" OR "CLBP" OR "NSLBP" OR "low back ache" OR "lower back ache" OR "back ache" OR "backache" OR "low back injury" OR "lower back injury" OR "back injury" OR "lumbar pain" OR "lumbago" OR "healthy" OR "pain-free" OR "symptom-free" OR "without pain" OR "subjects" OR "participants" OR "adults" OR "individuals" OR "volunteers") AND (LIMIT-TO (LANGUAGE, "English")) AND (LIMIT-TO (EXACTKEYWORD, "Human") OR LIMIT-TO (EXACTKEYWORD, "Humans") OR LIMIT-TO (EXACTKEYWORD, "Adult")) | 3,965,689 |
| ***Interface:*** Elsevier.  ***Search Screen:*** Advanced Search.  ***Database:*** Scopus.  ***Searching Date:*** 25 March 2022. | | |
