# Supplementary material for: Trunk postural control during unstable sitting among individuals with and without low back pain: A systematic review with an individual participant data meta-analysis
Source: PLoS One. 2024 Jan 24;19(1):e0296968. doi: 10.1371/journal.pone.0296968 (PMC10807788; doi:10.1371/journal.pone.0296968)
Supplement: S8 Table — (DOCX) [file pone.0296968.s009.docx]

| **Table S8.** Updated search strategy used in Web of Science Core Collection database | | |
| --- | --- | --- |
| **#** | **Query** | **Results** |
| S11 | S10 (Limiters: English Language) | 857 |
| S10 | S7 AND S8 AND S9 | 865 |
| S9 | S5 OR S6 | 15,150 |
| S8 | S3 OR S4 | 380,140 |
| S7 | S1 OR S2 | 773,300 |
| S6 | AB=(sit OR sitting OR unstable sitting OR seat OR unstable seat OR seated OR unstable seated OR chair OR wobble chair OR unstable chair) | 14,447 |
| S5 | TI=(sit OR sitting OR unstable sitting OR seat OR unstable seat OR seated OR unstable seated OR chair OR wobble chair OR unstable chair) | 2,317 |
| S4 | AB=(balance OR balance control OR postural balance OR postural control OR stability OR postural stability OR trunk stability OR spine stability OR motor control OR trunk control OR spine control OR postural sway OR equilibrium OR kinematics OR cent* of pressure OR CoP) | 370,046 |
| S3 | TI=(balance OR balance control OR postural balance OR postural control OR stability OR postural stability OR trunk stability OR spine stability OR motor control OR trunk control OR spine control OR postural sway OR equilibrium OR kinematics OR cent* of pressure OR CoP) | 51,917 |
| S2 | AB=(low back pain OR lower back pain OR back pain OR LBP OR CLBP OR NSLBP OR low back ache OR lower back ache OR back ache OR backache OR low back injury OR lower back injury OR back injury OR lumbar pain OR lumbago OR healthy OR pain-free OR symptom-free OR without pain OR subjects OR participants OR adults OR individuals OR volunteers) | 741,154 |
| S1 | TI=(low back pain OR lower back pain OR back pain OR LBP OR CLBP OR NSLBP OR low back ache OR lower back ache OR back ache OR backache OR low back injury OR lower back injury OR back injury OR lumbar pain OR lumbago OR healthy OR pain-free OR symptom-free OR without pain OR subjects OR participants OR adults OR individuals OR volunteers) | 106,685 |
| ***Interface:*** Clarivate.  ***Search Screen:*** Advanced Search.  ***Database:*** Web of Science Core Collection.  ***Updated Searching Date:*** 26 March 2022 - 7 September 2023. | | |
