# Supplementary material for: Trunk postural control during unstable sitting among individuals with and without low back pain: A systematic review with an individual participant data meta-analysis
Source: PLoS One. 2024 Jan 24;19(1):e0296968. doi: 10.1371/journal.pone.0296968 (PMC10807788; doi:10.1371/journal.pone.0296968)
Supplement: S9 Table — (DOCX) [file pone.0296968.s010.docx]

| **Table S9.** A checklist for comprehensiveness of reporting and methodological quality | | | |  |
| --- | --- | --- | --- | --- |
| **Domain** | **Item** | **Description of information that should be provided (R = report) or methodological issues that influence the quality of interpretation (Q = quality)** | **Available in published paper**  **(Y/P/N)** | **Available from author for IPD**  **(Y/P/N)** |
| Participant characteristics | Age | R: Summary measure *(e.g., range or mean ± standard deviation)* |  |  |
|  | Sex | R: Number or proportion of male/female |  |  |
|  | Height | R: Summary measure *(e.g., range or mean ± standard deviation)* |  |  |
|  | Weight | R: Summary measure *(e.g., range or mean ± standard deviation)* |  |  |
|  | Specific participant group | R: Information about whether or not the participants are from a specific participant group  *(e.g., participants of a specific sport; military; ethnicity; etc.)* |  |  |
|  | History of LBP | R: Information regarding whether pain-free controls/participants had history of LBP |  |  |
| LBP characteristics | Type | R: Type of LBP *(e.g., non-specific, stenosis, etc.)* |  |  |
|  | Symptom duration | R: Information about duration of LBP to determine whether pain is:  acute (0-6 weeks), subacute (7-12 weeks) or chronic (>12 weeks) |  |  |
|  | Severity level | R: Pain intensity level using a valid and reliable scale *(e.g., VAS, NPRS)* |  |  |
|  | Disability level | R: Disability level using a valid and reliable scale *(e.g., RMDQ, ODI)* |  |  |
|  | Psychological factors | R: Psychological factors identified using a valid and reliable scale *(e.g., PCS, FABQ)* |  |  |
| Experimental setup/protocol | Seat apparatus | R: Information about the seat build characteristics |  |  |
|  | Visual condition | R: Information about the visual condition *(e.g., eyes open and/or eyes closed)* |  |  |
|  | Trial duration | Q: A minimum duration of 30 seconds for each trial |  |  |
|  | Repetition | Q: At least three repetitions |  |  |
|  | Instructions | R: Instructions given to participants before recording |  |  |
|  | Sampling & filtering | R: Information about the sampling rate and applied low pass filter characteristics |  |  |
|  | Outcome measures | R: Clear description about how outcome measures were calculated  *(e.g., descriptions, equations or a citation of article)* |  |  |
|  | Excluded data | R: Information about excluded participants/trials and trials with safety bar/rail touch |  |  |
| Confounding effects control | Age | Q: Statistical adjustment for age |  |  |
|  | Sex | Q: Statistical adjustment for sex |  |  |
|  | Height | Q: Controlling or statistical adjustment for height |  |  |
|  | Weight | Q: Controlling or statistical adjustment for weight |  |  |
| Statistical information | Statistical method | R: Adequate information about the statistical methods used for analysis |  |  |
|  | Sample size | R: Information about power calculation |  |  |
| **Abbreviations:** Y, yes; P, partially; N, no; IPD, individual participant data; LBP, low back pain; VAS, Visual Analogue Scale; NPRS, Numeric Pain Rating Scale; RMDQ, Rolland-Morris Disability Questionnaire; ODI, Oswestry Disability Index; PCS, Pain Catastrophising Score; FABQ, Fear-Avoidance Beliefs Questionnaire.  **Scoring system:** Items are scored as ‘1’ (yes), ‘0.5’ (partially) or ‘0’ (no). The overall reporting/quality score is obtained from the sum of all scores converted to a percentage. Separate reporting/quality scores are calculated for each domain. Reporting/Quality scores range from 0 to 100%, with higher scores indicating higher reporting/quality. | | | | |
