# Supplementary material for: Trunk postural control during unstable sitting among individuals with and without low back pain: A systematic review with an individual participant data meta-analysis
Source: PLoS One. 2024 Jan 24;19(1):e0296968. doi: 10.1371/journal.pone.0296968 (PMC10807788; doi:10.1371/journal.pone.0296968)
Supplement: S10 Table — (DOCX) [file pone.0296968.s011.docx]

| **Table S10.** List of data used for the descriptive analysis | |
| --- | --- |
| **Characteristics** | First author, year of publication, and sample size  **Individuals with and without LBP**: Sex, age height, weight, and BMI  **Individuals with only LBP:** Pain type, pain form, pain duration, pain intensity, disability level, and psychological factors |
| **Experimental setup** | Seat apparatus, seat build characteristics, presence of a foot plate, presence of a safety rail, presence of a force plate, and type of recoded data |
| **Experimental protocol** | Position of arms, visual condition, task duration, number of repetitions, and given instructions |
| **Results**  *Main findings (e.g.)* | Differences in trunk postural control measures between individuals with and without LBP  Impact of visual condition or seat/task difficulty level on balance performance  Effects of covariates (e.g., sex, age, BMI, and bar touch) on balance performance  Effects of LBP clinical features (e.g., pain intensity, disability level or psychological factors) on balance performance  Reliability of trunk postural control measures (e.g., CoP or seat angle parameters) during unstable sitting tasks |
| **Abbreviations:** LBP, low back pain; BMI, body mass index; CoP, centre of pressure. | |
