# Supplementary material for: Trunk postural control during unstable sitting among individuals with and without low back pain: A systematic review with an individual participant data meta-analysis
Source: PLoS One. 2024 Jan 24;19(1):e0296968. doi: 10.1371/journal.pone.0296968 (PMC10807788; doi:10.1371/journal.pone.0296968)
Supplement: S11 Table — (DOCX) [file pone.0296968.s012.docx]

| **Table S11.** Inclusion and exclusion criteria for studies with data from individuals with LBP | | |
| --- | --- | --- |
| **Study** | **Inclusion criteria** | **Exclusion criteria** |
| Radebold et al. [22] | **LBP:** A persisting or periodic pain lasting longer than 6 months. LBP radiographs showed only normal, age-related changes.  **Control:** No history of any neuromuscular or postural disorder and no back pain lasting longer than 3 consecutive days.  **Matching between groups:** Age, height and weight. | **LBP:** Neurologic deficits, structural deformities, genetic spinal disorders, or previous spinal surgery.  **LBP & Control:** Vestibular or visual disorders. |
| Reeves et al. [73] | **LBP:** Persistent or intermittent pain lasting longer than 6 months. | **LBP:** Spinal surgery, electrical implants (i.e., cardiac pacemakers, drug delivery pumps, etc.), history of Parkinson, multiple sclerosis, cerebral palsy, Alzheimer, amyotrophic lateral sclerosis, stroke, seizures, head trauma, urinary incontinence, vestibular or vision problems, orthostatic hypotension, or blind, pregnant or lactating. |
| Navalgund [72] | **LBP:** A primary complaint of subacute, recurrent LBP. LBP subjects also included if the duration of pain in the current episode was less than or equal to 8 weeks and had experienced at least 1 separate episode in the past year.  **Control:** No history of significant LBP in the past 2 years.  **Matching between groups:** Age, gender, height and weight. | **General (both groups):** History of lumbar surgery and presence of sciatica or medical conditions which might affect spinal control such as cauda equina syndrome, neurological disorders, fracture, cancer, infection, or systemic disease. |
| van Dieën et al. [33] | **LBP:** Current-LBP and recent-LBP (no current pain, but pain within last 12 months).  **Control:** No LBP in last 12 months.  **Matching between groups:** Age. | **General (both groups):** Prior back surgery and previously diagnosed specific LBP, such as due to disc. Self-reported or diagnosed disorders that might interfere with balance. |
| van Dieën et al. [74] | **LBP:** Not available/reported.  **Control:** Not available/reported.  **Matching between groups:** Age. | **LBP:** Not available/reported.  **Control:** Not available/reported. |
| Willigenburg et al. [31] | **LBP:** LBP during the last 6 weeks or longer.  **Control:** No LBP during the previous year. | **LBP:** Any specific diagnosis, previous spine surgery or scored >105 on a questionnaire identifying psychosocial risk factors. |
| Larivière et al. [24] | **LBP:** Lumbar or lumbosacral pain with or without proximal radicular pain (limited distally at the knees) and presence of chronic pain defined as a daily or almost daily pain for at least 3 months. | **General (both groups):** Surgery of the pelvis or spinal column; scoliosis; systemic or degenerative disease; BMI over 31.5 kg^2^/m (women) or 33 kg^2^/m (men); one positive response to the Physical Activity Readiness Questionnaire; history of neurological diseases or deficits not related to back pain (e.g., stroke, peripheral neuropathies, balance deficits); anticonvulsive, antidepressive and anxiolitic medication (use of antispasmodic, anti-inflammatory and analgesic drugs for back pain was accepted); pregnancy and claustrophobia.  **Control:** Back pain in the preceding year or back pain lasting more than 1 week the years before. |
| **Abbreviations:** LBP, low back pain; BMI, body mass index. | | |

| **Table S11.** Inclusion and exclusion criteria for studies with data from individuals with LBP (cont.) | | |
| --- | --- | --- |
| **Study** | **Inclusion criteria** | **Exclusion criteria** |
| Larivière et al. [34] | **LBP:** Lumbar or lumbosacral pain with or without proximal radicular pain (limited distally at the knees) and presence of chronic pain defined as a daily or almost daily pain for at least three months. | **General (both groups):** Surgery of the pelvis or spinal column; scoliosis; systemic or degenerative disease; BMI over 31.5 kg^2^/m (women) or 33 kg^2^/m (men); one positive response to the Physical Activity Readiness Questionnaire; history of neurological diseases or deficits not related to back pain (e.g., stroke, peripheral neuropathies, balance deficits); anticonvulsive, antidepressive or anxiolitic medication (use of antispasmodic, anti-inflammatory and analgesic drugs for back pain was accepted); pregnancy or claustrophobia.  **Control:** Back pain in the preceding year or back pain lasting more than one week the years before. |
| Sung et al. [19] | **LBP:** Acute to subacute LBP (duration of less than 3 months by self-report of history) who were identified to have movement coordination impairments through clinical examination. Bilateral or unilateral LBP in the region between T12 and sacrum, average pain greater than 3 of 10 on a Numeric Pain Rating Scale, and Oswestry Disability Index of 20% or greater. If they had a history of LBP, they were included in the study if their previous symptoms had gone into remission for at least 6 months, they had returned to premorbid levels of activity, and this episode was a recent exacerbation of their pain.  **Control:** No history of LBP that required medical or allied health intervention or reduced their functional activities for greater than 3 days. They did not participate in regular core stabilization exercises.  **Matching between groups:** Age, gender and BMI. | **LBP:** Reporting rehabilitation intervention for the current episode of LBP. Facture, osteoporosis, and tumor, frank neurologic signs, a BMI of greater than 30, reported spinal surgeries, pregnancy, or lower extremity injury. |
| Cyr et al. [30] | **LBP:** Continuous or recurring back pain for more than 3 months, had a minimum weekly average pain intensity of 3 on the numeric pain rating scale (rated 0 to 10), experienced back pain at least 3 days/week on average, and were able to walk without an assistive device.  **Control:** No history of back pain.  **Matching between groups:** Age and gender. | **General (both groups):** A BMI >30, back surgery within the past year, spinal deformities, reported Meniere’s disease or vertigo, neurological, or cardiovascular issues, recent head trauma, or were pregnant. |
| Shahvarpour et al. [29] | **LBP:** Lumbar or lumbosacral pain (with or without radicular pain) for at least four weeks (non-acute phase), and a score higher than 12% on the Oswestry disability index to allow minimum important change of 10%. Only non-acute patients were recruited since exercise is not the primary intervention strategy in nonacute LBP.  **Control:** All healthy controls had to meet a BMI ≤30 kg/m^2^ as the amount of subcutaneous tissues and fat are well known to act as confounding factors for some of the measures (e.g. ultrasound imaging; surface electromyography). This might also be the case for the trunk postural balance test as subcutaneous fat is not distributed the same way between subjects. Consequently, only the recruited patients that also met this criterion were retained for neuromuscular testing. | **General (both groups):** Surgery on the pelvis or spinal column; a specific lumbar pathology (fracture, infection or tumor) or scoliosis; systemic or degenerative disease; the initiation of an exercise program in the last three months; pregnancy; or claustrophobia. Litigation relating to a back injury; and the presence of one positive neurological sign in two of three test categories: reduced Achilles and patellar tendon reflexes; reduced strength in lumbosacral myotomes; reduced sensation in lumbosacral dermatomes.  **Control:** Back pain in the preceding year, or a history of back pain lasting more than one week. |
| **Abbreviations:** LBP, low back pain; BMI, body mass index. | | |

| **Table S11.** Inclusion and exclusion criteria for studies with data from individuals with LBP (cont.) | | |
| --- | --- | --- |
| **Study** | **Inclusion criteria** | **Exclusion criteria** |
| Shahvarpour et al. [32] | **LBP & Control:** Mastery of French or English; being currently employed, or, for subjects with LBP, having been employed before the current episode of LBP.  **LBP:** Lumbar or lumbosacral pain (with or without radicular pain) for at least 4 weeks (non-acute phase); no radicular pain below the knees. | **General (both groups):** Pelvic or spinal surgery; specific lumbar pathology (fracture, infection or tumor); scoliosis; systemic or degenerative disease; BMI >30 kg/m^2^; high blood pressure (systolic >140 mmHg and/or diastolic >90 mmHg); history of neurological condition other than those related to back pain; anxiolytic, anticonvulsant, antidepressant, or other medication which can influence neuronal excitability (antispasmodic, anti-inflammatory and analgesic medications were accepted); sacroiliac pain as identified with five clinical tests; and pending litigation over a compensation.  **Control:** Presence of back pain in the last year, or having a history of back pain lasting more than a week. |
| Shahvarpour et al. [75] | **LBP:** Lumbar or lumbosacral pain with or without proximal radicular pain (limited distally at the knees) and presence of chronic pain defined as a daily or almost daily pain for at least 3 months.  **LBP & Control:** Body height close to the finite element model (vertical distance from the S1 to the C7 of 46.76 cm). | **Control:** Back pain in the previous year or back pain lasting longer than a week during the preceding years. |
| Larivière et al. [76] | **LBP:** Lumbar or lumbosacral pain, with or without radicular pain, for at least four weeks (non-acute); a score above 12% on the Oswestry Disability Index (version 2.1a). | **General (both groups):** Surgery of the pelvis or spinal column; existence of a specific lumbar pathology (fracture, infection or tumor) or scoliosis; existence of a systemic or degenerative disease; BMI over 30 kg/m^2^.  **Control:** Back pain in the past year, or history of back pain lasting for more than 1 week. |
| van den Hoorn et al. [35] | **LBP:** An acute episode of LBP that began within the last two weeks (preceded by at least 1 month without pain) and present with pain and/or disability at the time of testing. An acute LBP episode was defined as pain between the gluteal fold and 12th thoracic vertebrae that lasted for >24 hours, affected their function and caused them to seek or seriously consider treatment.  **Control:** No pain or disability at the time of testing and no history of LBP in the past 12 months. | **General (both groups):** Other major pain or injury in any body region in the previous 12 months, suspected spinal pathology (e.g., fracture, inflammatory/infective spinal disease, causa equina syndrome, metastasis or neurological disorders), major diseases, or were <18 or >50 years old. |
| **Abbreviations:** LBP, low back pain; BMI, body mass index. | | |
