# Supplementary material for: Trunk postural control during unstable sitting among individuals with and without low back pain: A systematic review with an individual participant data meta-analysis
Source: PLoS One. 2024 Jan 24;19(1):e0296968. doi: 10.1371/journal.pone.0296968 (PMC10807788; doi:10.1371/journal.pone.0296968)
Supplement: S12 Table — (DOCX) [file pone.0296968.s013.docx]

| **Table S12.** Inclusion and exclusion criteria for studies with data from only pain-free individuals | | |
| --- | --- | --- |
| **Study** | **Inclusion criteria** | **Exclusion criteria** |
| Cholewicki et al. [21] | Free of any back pain for at least 1 year. | Not available/reported. |
| Silfies et al. [77] | No history or evidence of postural, musculoskeletal, visual or vestibular disorders. | Not available/reported. |
| van der Burg et al. [79] | Not available/reported. | Not available/reported. |
| Reeves et al. [78] | Free of any back pain for at least 1 year, No subjects reported having neurological or musculoskeletal problems. | Not available/reported. |
| Cholewicki et al. [80] | Healthy subjects. | Not available/reported. |
| Lee and Granata [81] | No history of low back pain. | Not available/reported. |
| Lee et al. [82] | No history of chronic low-back pain. | Not available/reported. |
| Slota et al. [23] | No previous history of low back pain. | Not available/reported. |
| Hendershot & Nussbaum [83] | No low back pain at the time of testing. | Any recent history (6 months) of falls, neurologic deficits, or any underlying musculoskeletal disorders that could confound the results. |
| Hendershot et al. [84] | Free of current or recent injuries, illnesses, musculoskeletal disorders, and other health-related aspects that may have influenced the results. | Not available/reported. |
| Barbado et al. [86] | Male Judokas (black belt) with more than 7 years of experience and a work out frequency of 3-5 days per week. | Not available/reported. |
| Barbado et al. [85] | Male Judokas, kayakers and recreational athletes. Judokas and kayakers were competitive athletes and had more than 4 years of experience in national and/or international championships. The recreational athletes were physically-active men with a work-out frequency of 2-3 days per week. | A recent history of back injury, abdominal surgery or inguinal hernia, and neurological or musculoskeletal disorders. |
| Beaudette et al. [87] | Not available/reported. | Presence of pain within the lumbopelvic or lower-limb region as well as any diagnosed sensory or motor deficit disorders. |
| Ruggiero et al. [88] | Healthy female participants. | A history of low-back injury, chronic low-back pain, allergies to adhesives or any skin conditions affecting the back. |
| Barbado et al. [28] | Healthy male subjects without current pain in the hip or back, or past pathology in these regions. No subjects reported having neurologic or musculoskeletal problems. | Female subjects, in an attempt to minimize potential variability resulting from anthropometric gender differences. |
| Barbado et al. [89] | Healthy male subjects without current pain in the hip or back or past pathology in these regions. No subjects reported having no neurological or musculoskeletal problems. No subjects had previous experience in the balance task used in this study. | Not available/reported. |

| **Table S12.** Inclusion and exclusion criteria for studies with data from only pain-free individuals (cont.) | | |
| --- | --- | --- |
| **Study** | **Inclusion criteria** | **Exclusion criteria** |
| Glofcheskie & Brown [90] | Males competing in collegiate level long distance running, golf, and controls with no athletic experience. Long distance runners and golfers were current members of a collegiate varsity team, with a minimum of 7 years’ experience in their respective sport. Absence of low back pain and related musculoskeletal and neurological disorders in all participants. | Not available/reported. |
| Acasio et al. [91] | Participants with no current or recent history of illness, injury, or musculoskeletal disorders within the past 12 months. | Not available/reported. |
| Williams et al. [92] | A sample of nondisabled participants without history of neurological or musculoskeletal disorders, nor was experiencing back pain chronically or acutely at time of testing. | Not available/reported. |
| Barbado et al. [93] | No pain in the hip or back; no past pathology in these regions; and neurologic or musculoskeletal problems. | Not available/reported. |
| Roberts & Vette [25] | Non-disabled, young and male individuals without history of neurological or musculoskeletal impairments or pain, gait or balance difficulties, or use of a walking aid. | Not available/reported. |
| Roberts et al. [27] | Non-disabled, young and male individuals without history of neurological or musculoskeletal impairments or pain, gait or balance difficulties, or use of a walking aid. | Not available/reported. |
| Acasio et al. [94] | No current or recent history of illness, injury, or musculoskeletal disorders. | Not available/reported. |
| Alshehri et al. [26] | Healthy pain-free participants. | Had a diagnosis of inflammatory/infective spinal disease(s), fracture, cauda equina syndrome or a neurological disorder(s), were <18 or >50 years old, had major pain/injury to any body region in the previous 12 months, or other major disease(s)/disorder(s). |
| de Oliveira et al. [95] | Participants did not participate in physical exercise programs, did not present with low back pain, absence of any musculoskeletal, joint or overall health conditions that would limit implementation of the study protocol, age between 18 and 40 years old. | Participants who were not able to perform the proposed activities. |
