# Supplementary material for: Trunk postural control during unstable sitting among individuals with and without low back pain: A systematic review with an individual participant data meta-analysis
Source: PLoS One. 2024 Jan 24;19(1):e0296968. doi: 10.1371/journal.pone.0296968 (PMC10807788; doi:10.1371/journal.pone.0296968)
Supplement: S13 Table — (DOCX) [file pone.0296968.s014.docx]

| **Table S13.** Experimental setup and protocol used in studies with data from individuals with LBP | | | | | | | | | | |
| --- | --- | --- | --- | --- | --- | --- | --- | --- | --- | --- |
| **Study** | **Experimental setup** | | | | | **Experimental protocol** | | | | |
|  | **Seat apparatus**  ***Seat build characteristics*** | **Foot plate** | **Safety bar** | **Force plate** | **Recorded data** | **Arms crossed** | **Visual condition** | **Trial duration (s)** | **Repetition (n)** | **Given (specific) instructions** |
| Radebold et al. [22] | Hemisphere  *L0: infinity, flat surface*  *L1: radius 25 cm; height NA*  *L2: radius 22 cm; height NA*  *L3: radius 11 cm; height NA* | ✓ | ✓ | ✓ | CoP | ✓ | EO, EC | 7 | 5 | “maintain balance while sitting upright” |
| Reeves et al. [73] | Hemisphere  *L1: radius 20 cm; height NA*  *L2: radius 15 cm; height NA* | ✓ | ✓ | ✓ | CoP | ✓ | EC | 20 | 4 | “maintain balance” |
| Navalgund [72] | Springs  *L1: height NA; stiffness NA; R_spring_ 80%*  *L2: height NA; stiffness NA; R_spring_ 65%*  *L3: height NA; stiffness NA; R_spring_ 50%* | ✓ | ☓ | ✓ | CoP | ✓ | EO | 60 | 3 | “maintain an upright posture” |
| van Dieën et al. [33] | Hemisphere  *Radius 19.5 cm; height NA* | ✓ | ✓ | ✓ | CoP | ☓ | EO | 30 | 3 | “sit as quietly as possible” |
| van Dieën et al. [74] | Hemisphere  *Radius 19.5 cm; height NA* | ✓ | ✓ | ✓ | CoP | ☓ | EO | 30 | 3 | “sit as quietly as possible” |
| Willigenburg et al. [31] | Hemisphere  *Radius 25 cm; height 17 cm* | ✓ | ✓ | ✓ | CoP | ✓ | EO, EC | 50 | 2 | “sit as quietly as possible” |
| Larivière et al. [24] | Springs  *Height 4.5 cm; stiffness 8467 N/m; R_spring_ 60%* | ✓ | ✓ | ✓ | CoP  Seat angle | ✓ | EC | 60 | 3 | NA |
| Larivière et al. [34] | Springs  *Height 4.5 cm; stiffness 8467 N/m; R_spring_ 60%* | ✓ | ✓ | ✓ | CoP  Seat angle | ✓ | EC | 60 | 3 | NA |
| Sung et al. [19] | Hemisphere  *Radius 22 cm; height 9 cm* | ✓ | ✓ | ✓ | CoP | ✓ | EO, EC | 60 | 3 | “move as little as possible” |
| Shahvarpour et al. [75] | Springs  *Height 4.5 cm; stiffness 8467 N/m; R_spring_ 60%* | ✓ | ✓ | ✓ | CoP  Seat angle | ✓ | EC | 60 | 3 | “sit relaxed with the head and chest in the upright position” |
| **Abbreviations:** LBP, low back pain; L, level; NA, not available; CoP, center of pressure; EO, eyes open; EC, eyes closed; R_spring_, distance (radius) of springs from the pivot in percentage. | | | | | | | | | | |

| **Table S13.** Experimental setup and protocol used in studies with data from individuals with LBP (cont.) | | | | | | | | | | |
| --- | --- | --- | --- | --- | --- | --- | --- | --- | --- | --- |
| **Study** | **Experimental setup** | | | | | **Experimental protocol** | | | | |
|  | **Seat apparatus**  ***Seat build characteristics*** | **Foot plate** | **Safety bar** | **Force plate** | **Recorded data** | **Arms crossed** | **Visual condition** | **Trial duration (s)** | **Repetition (n)** | **Given (specific) instructions** |
| Shahvarpour et al. [29] | Springs  *Height 4.5 cm; stiffness 8467 N/m; R_spring_ 60%* | ✓ | ✓ | ☓ | Seat angle | ✓ | EC | 60 | 3 | NA |
| Shahvarpour et al. [32] | Springs  *Height 4.5 cm; stiffness 8467 N/m; R_spring_ 60%* | ✓ | ✓ | ☓ | Seat angle | ✓ | EC | 60 | 3 | NA |
| Cyr et al. [30] | Hemisphere  *Radius NA; height NA* | ✓ | ✓ | ✓ | CoP | ✓ | EO, FB, EC | 30 | 3 | “maintain balance” |
| Larivière et al. [76] | Springs  *Height 4.5 cm; stiffness 8467 N/m; R_spring_ 60%* | ✓ | ✓ | ☓ | Seat angle | ✓ | EC | 60 | 3 | NA |
| van den Hoorn et al. [35] | Hemisphere  *Radius 25 cm; height 19 cm* | ✓ | ✓ | ✓ | CoP | ✓ | EO, FB, EC | 30 | 3 | EO & EC: “to sit upright as quietly as possible”  FB: “keep the lines as close together as possible” |
| **Abbreviations:** LBP, low back pain; R_spring_, distance (radius) of springs from the pivot in percentage; EC, eyes closed; NA, not available; CoP, center of pressure; EO, eyes open; FB, feedback. | | | | | | | | | | |
