# Supplementary material for: Trunk postural control during unstable sitting among individuals with and without low back pain: A systematic review with an individual participant data meta-analysis
Source: PLoS One. 2024 Jan 24;19(1):e0296968. doi: 10.1371/journal.pone.0296968 (PMC10807788; doi:10.1371/journal.pone.0296968)
Supplement: S14 Table — (DOCX) [file pone.0296968.s015.docx]

| **Table S14.** Experimental setup and protocol used in studies with data from only pain-free individuals | | | | | | | | | | |
| --- | --- | --- | --- | --- | --- | --- | --- | --- | --- | --- |
| **Study** | **Experimental setup** | | | | | **Experimental protocol** | | | | |
|  | **Seat apparatus**  ***Seat build characteristics*** | **Foot plate** | **Safety bar** | **Force plate** | **Recorded data** | **Arms crossed** | **Visual condition** | **Trial duration (s)** | **Repetition (n)** | **Given (specific) instructions** |
| Cholewicki et al. [21] | Hemisphere  *L0: infinity, flat surface*  *L1: radius 25 cm; height NA*  *L2: radius 22 cm; height NA*  *L3: radius 11 cm; height NA* | ✓ | ✓ | ✓ | CoP | ✓ | EO, EC | 7 | 5 | “maintain his/her balance” |
| Silfies et al. [77] | Hemisphere  *L0: infinity, flat surface*  *L1: radius 25 cm; height 8.2 cm*  *L2: radius 25 cm; height 13.2 cm*  *L3: radius 25 cm; height 18.2 cm* | ✓ | ✓ | ✓ | CoP | ✓ | EO, EC | 60 | 4 | “maintain their sitting balance” |
| van der Burg et al. [79] | Hemisphere  *Radius 19.5 cm; height NA* | ✓ | ✓ | ✓ | CoP | ☓ | EO | 15 | 5 | “sit as quietly as possible” |
| Reeves et al. [78] | Hemisphere  *Radius 15 cm; height NA* | ✓ | ✓ | ✓ | CoP | ✓ | EO, EC | 20 | 3 | “maintain his/her balance” |
| Cholewicki et al. [80] | Hemisphere  *Radius 15 cm; height NA* | ✓ | ✓ | ✓ | CoP | ✓ | EO | 20 | 3 | “maintain his/her balance” |
| Lee and Granata [81] | Springs  *L1: height NA; stiffness NA; R_spring_ 100%*  *L2: height NA; stiffness NA; R_spring_ 75%*  *L3: height NA; stiffness NA; R_spring_ 50%* | ✓ | ☓ | ✓ | CoP  Seat angle | ✓ | EO | 60 | 5 | “maintain seated balance” |
| Lee et al. [82] | Springs  *Height NA; stiffness NA; R_spring_ 50%* | ✓ | ☓ | ☓ | Seat angle | ✓ | EO | 60 | 5 | “maintain seated balance” |
| Slota et al. [23] | Springs  *Height NA; stiffness 99.82 N/cm; R_spring_ 43.5%* | ✓ | ☓ | ✓ | CoP  Seat angle | ✓ | EO | 60 | 4 | “sit with an upright posture” |
| Hendershot & Nussbaum [83] | Springs  *Height NA; stiffness NA; R_spring_ 60%* | ✓ | ☓ | ✓ | CoP | ✓ | EO | 65 | 3 | “keep the chair surface as level as possible” |
| Hendershot et al. [84] | Springs  *Height NA; stiffness NA; R_spring_ 60%* | ✓ | ☓ | ✓ | CoP | ✓ | EO | 60 | 6 | “keep the chair surface as level as possible” |
| **Abbreviations:** L, level; NA, not available; CoP, center of pressure; EO, eyes open; EC, eyes closed; R_spring_, distance (radius) of springs from the pivot in percentage. | | | | | | | | | | |

| **Table S14.** Experimental setup and protocol used in studies with data from only pain-free individuals (cont.) | | | | | | | | | | |
| --- | --- | --- | --- | --- | --- | --- | --- | --- | --- | --- |
| **Study** | **Experimental setup** | | | | | **Experimental protocol** | | | | |
|  | **Seat apparatus**  ***Seat build characteristics*** | **Foot plate** | **Safety bar** | **Force plate** | **Recorded data** | **Arms crossed** | **Visual condition** | **Trial duration (s)** | **Repetition (n)** | **Given (specific) instructions** |
| Barbado et al. [86] | Hemisphere  *Radius 17.5 cm; height 12 cm* | ✓ | ✓ | ✓ | CoP | ✓ | EO, FB | 70 | 2 | EO: “to sit still in their preferred seated position”  FB: “align their CoP position with the target point located in the center of the screen” |
| Barbado et al. [85] | Hemisphere  *Radius 17.5 cm; height 12 cm* | ✓ | ✓ | ✓ | CoP | ✓ | EO, FB | 70 | 2 | EO: “to sit still in their preferred seated position”  FB: “align their CoP position with the target point located in the center of the screen” |
| Beaudette et al. [87] | Hemisphere  *Radius 10 cm; height 11 cm* | ☓ | ☓ | ✓ | CoP | ✓ | EO | 60 | 5 | “remain as stable as possible” |
| Ruggiero et al. [88] | Hemisphere  *Radius 10 cm; height 11 cm* | ☓ | ☓ | ✓ | CoP | ✓ | EO | 50 | 3 | “maintain their torso in a vertical position” |
| Barbado et al. [28] | Hemisphere  *Radius 17.5 cm; height 12 cm* | ✓ | ✓ | ✓ | CoP | ☓ | EO | 70 | 5 | “keeping the unstable seat as still as possible” |
| Barbado et al. [89] | Hemisphere  *Radius 17.5 cm; height 12 cm* | ✓ | ✓ | ✓ | CoP | ☓ | EO | 70 | 5 | “maintain their balance, keeping the unstable platform as still as possible” |
| Glofcheskie & Brown [90] | Hemisphere  *Radius 10 cm; height 11 cm* | ☓ | ☓ | ✓ | CoP | ✓ | EO | 60 | 5 | “maintain balance in an upright trunk posture” |
| Acasio et al. [91] | Springs  *L1: height NA; stiffness NA; R_spring_ 100%*  *L2: height NA; stiffness NA; R_spring_ 75%*  *L3: height NA; stiffness NA; R_spring_ 60%*  *L4: height NA; stiffness NA; R_spring_ 45%* | ✓ | ☓ | ✓ | CoP | ✓ | EO | 60 | 1 | “keep the chair level” |
| **Abbreviations:** CoP, center of pressure; EO, eyes open; FB, feedback; L, level; NA, not available; R_spring_, distance (radius) of springs from the pivot in percentage. | | | | | | | | | | |

| **Table S14.** Experimental setup and protocol used in studies with data from only pain-free individuals (cont.) | | | | | | | | | | |
| --- | --- | --- | --- | --- | --- | --- | --- | --- | --- | --- |
| **Study** | **Experimental setup** | | | | | **Experimental protocol** | | | | |
|  | **Seat apparatus**  ***Seat build characteristics*** | **Foot plate** | **Safety bar** | **Force plate** | **Recorded data** | **Arms crossed** | **Visual condition** | **Trial duration (s)** | **Repetition (n)** | **Given (specific) instructions** |
| Williams et al. [92] | Hemisphere  *L1: radius 25 cm; height 6.25 cm*  *L2: radius 20 cm; height 6.25 cm*  *L3: radius 15 cm; height 6.25 cm*  *L4: radius 13 cm; height 6.25 cm*  *L5: radius 11 cm; height 6.25 cm* | ☓ | ☓ | ☓ | Seat angle | ✓ | EO, EC | 30 | 3 | “maintain, as much as possible, an upright posture” |
| Barbado et al. [93] | Hemisphere  *Radius 20 cm; height 12 cm* | ✓ | ✓ | ✓ | CoP | ✓ | EO, FB | 70 | 2 | EO: “sit still in their preferred seated position”  FB: “align their CoP position with the target point located in the center of the screen” |
| Roberts & Vette [25] | Hemisphere  *L1: radius 20 cm; height 6.25 cm*  *L2: radius 13 cm; height 6.25 cm* | ☓ | ☓ | ☓ | Seat angle | ✓ | EO (L2)  EC (L1) | 35 | 4 | “not to move the lower limbs during the test” |
| Roberts et al. [27] | Hemisphere  *L1: radius 20 cm; height 6.25 cm*  *L2: radius 13 cm; height 6.25 cm* | ☓ | ☓ | ☓ | Seat angle | ✓ | EO (L2)  EC (L1) | 35 | 4 | “maintain this posture (an upright posture)” |
| Acasio et al. [94] | Springs  *L1: height NA; stiffness NA; R_spring_ 100%*  *L2: height NA; stiffness NA; R_spring_ 75%*  *L3: height NA; stiffness NA; R_spring_ 60%*  *L4: height NA; stiffness NA; R_spring_ 45%* | ✓ | ☓ | ✓ | CoP | ✓ | EO | 60 | 1 | “keep the chair as level as possible” |
| Alshehri et al. [26] | Hemisphere  *Radius 25 cm; height 19 cm* | ✓ | ✓ | ✓ | Seat angle | ✓ | EO, FB, EC | 30 | 3 | “sit as still as possible” |
| de Oliveira et al. [95] | Springs  *Height 5 cm; stiffness NA; distance from the pivot 15 cm* | ✓ | ✓ | ✓ | CoP | ✓ | EO | 30 | 2 | NA |
| **Abbreviations:** L, level; EO, eyes open; EC, eyes closed; CoP, center of pressure; FB, feedback; NA, not available; R_spring_, distance (radius) of springs from the pivot in percentage. | | | | | | | | | | |
