# Supplementary material for: Trunk postural control during unstable sitting among individuals with and without low back pain: A systematic review with an individual participant data meta-analysis
Source: PLoS One. 2024 Jan 24;19(1):e0296968. doi: 10.1371/journal.pone.0296968 (PMC10807788; doi:10.1371/journal.pone.0296968)
Supplement: S16 Table — (DOCX) [file pone.0296968.s017.docx]

| **Table S16.** Reporting/Quality scores for studies with data from individuals with LBP: available from IPD | | | | | | | | | | | | | | | | | | | | | | | | | | | | | | | |
| --- | --- | --- | --- | --- | --- | --- | --- | --- | --- | --- | --- | --- | --- | --- | --- | --- | --- | --- | --- | --- | --- | --- | --- | --- | --- | --- | --- | --- | --- | --- | --- |
| **Study** | **Participant characteristics** | | | | | | | **LBP characteristics** | | | | | | **Experimental setup/protocol** | | | | | | | | | **Confounding effects control** | | | | | **Statistical information** | | | **Total score** |
|  | **A** | **B** | **C** | **D** | **E** | **F** | **Score** | **G** | **H** | **I** | **J** | **K** | **Score** | **L** | **M** | **N** | **O** | **P** | **Q** | **R** | **S** | **Score** | **T** | **U** | **V** | **W** | **Score** | **X** | **Y** | **Score** |  |
| Radebold et al. [22] | 1 | 1 | 1 | 1 | 1 | 1 | 100 | 1 | 1 | 1 | 1 | 0 | 80 | ½ | 1 | 0 | 1 | 1 | 1 | 1 | 1 | 81.3 | 1 | 1 | 1 | 1 | 100 | 1 | 0 | 50 | 87.5 |
| Reeves et al. [73] | 1 | 1 | 1 | 1 | 1 | - | 100 | 1 | 1 | 1 | 1 | 0 | 80 | ½ | 1 | 0 | 1 | 1 | 1 | 1 | 1 | 81.3 | 1 | 1 | 1 | 1 | 100 | 1 | 0 | 50 | 87 |
| van Dieën et al. [33] | 1 | 1 | 1 | 1 | 1 | 1 | 100 | 1 | 0 | 0 | 0 | 0 | 20 | ½ | 1 | 1 | 1 | 1 | 1 | 1 | ½ | 87.5 | 1 | 1 | 1 | 1 | 100 | 1 | 0 | 50 | 78.3 |
| Willigenburg et al. [31] | 1 | 1 | 1 | 1 | 1 | 1 | 100 | 1 | 1 | 1 | 0 | 0 | 60 | 1 | 1 | 1 | 0 | 1 | 1 | 1 | 1 | 87.5 | 1 | 1 | 1 | 1 | 100 | 1 | 0 | 50 | 84 |
| Larivière et al. [34] | 1 | 1 | 1 | 1 | 1 | 1 | 100 | 1 | 1 | 1 | 1 | 1 | 100 | 1 | 1 | 1 | 1 | 0 | 1 | 1 | 1 | 87.5 | 1 | 1 | 1 | 1 | 100 | 1 | 0 | 50 | 92 |
| Sung et al. [19] | 1 | 1 | 1 | 1 | 1 | 1 | 100 | 1 | 1 | 1 | 1 | 1 | 100 | 1 | 1 | 1 | 1 | 1 | 1 | 1 | 1 | 100 | 1 | 1 | 1 | 1 | 100 | 1 | 1 | 100 | 100 |
| Shahvarpour et al. [29] | 1 | 1 | 1 | 1 | 1 | 1 | 100 | 1 | 1 | 1 | 1 | 1 | 100 | 1 | 1 | 1 | 1 | 0 | 1 | 1 | 1 | 87.5 | 1 | 1 | 1 | 1 | 100 | 1 | 0 | 50 | 92 |
| Shahvarpour et al. [32] | 1 | 1 | 1 | 1 | 1 | 1 | 100 | 1 | 1 | 1 | 1 | 1 | 100 | 1 | 1 | 1 | 1 | 0 | 1 | 1 | 1 | 87.5 | 1 | 1 | 1 | 1 | 100 | 1 | 0 | 50 | 92 |
| Cyr et al. [30] | 1 | 1 | 1 | 1 | 1 | 1 | 100 | 1 | 1 | 1 | 1 | 1 | 100 | 0 | 1 | 1 | 1 | 1 | ½ | 1 | 1 | 81.3 | 1 | 1 | 1 | 1 | 100 | 1 | 0 | 50 | 91.7 |
| van den Hoorn et al. [35] | 1 | 1 | 1 | 1 | 1 | 1 | 100 | 1 | 1 | 1 | 1 | 1 | 100 | 1 | 1 | 1 | 1 | 1 | 1 | 1 | 1 | 100 | 1 | 1 | 1 | 1 | 100 | 1 | 0 | 50 | 96 |
|  | **Total score** | | | | | | | | | | | | | | | | | | | | | | | | | | | | | | |
| All studies (*n*=10) | 100 | 100 | 100 | 100 | 100 | 100 | 100 | 100 | 90 | 90 | 80 | 60 | 84 | 75 | 100 | 80 | 90 | 70 | 95 | 100 | 95 | 88.1 | 100 | 100 | 100 | 100 | 100 | 100 | 10 | 55 | 90 |
| **Abbreviations:** LBP, low back pain; IPD, individual participant data.  **Checklist Items:** **A**, report summary measure of age; **B**, report number or proportion of male/female; **C**, report summary measure of height; **D**, report summary measure of weight; **E**, report information about whether the participants are from a specific participant group; **F**, report information about if pain-free controls/participants had history of LBP; **G**, report type of LBP; **H**, report information about duration of LBP to determine if pain is acute, subacute or chronic; **I**, report pain intensity level using a valid and reliable scale; **J**, report disability level using a valid and reliable scale; **K**, report psychological factors using a valid and reliable scale; **L**, report information about the seat build characteristics; **M**, report information about the visual condition; **N**, use a minimum duration of 30 seconds for each trial (quality); **O**, use at least three repetitions (quality); **P**, report instructions given to participants before recording; **Q**, report information about the sampling rate and applied low pass filter characteristics; **R**, report a clear description about how outcome measures were calculated; **S**, report information about the excluded participants/trials; **T**, statistical adjustment for age (quality); **U**, statistical adjustment for sex (quality); **V**, controlling or statistical adjustment for height (quality); **W**, controlling or statistical adjustment for weight (quality); **X**, report adequate information about the statistical methods used for analysis; **Y**, report information about the power calculation. | | | | | | | | | | | | | | | | | | | | | | | | | | | | | | | |
