# Supplementary material for: Trunk postural control during unstable sitting among individuals with and without low back pain: A systematic review with an individual participant data meta-analysis
Source: PLoS One. 2024 Jan 24;19(1):e0296968. doi: 10.1371/journal.pone.0296968 (PMC10807788; doi:10.1371/journal.pone.0296968)
Supplement: S17 Table — (DOCX) [file pone.0296968.s018.docx]

| **Table S17.** Reporting/Quality scores for studies with data from only pain-free individuals: available from published papers | | | | | | | | | | | | | | | | | | | | | | | | | | | | | | | |
| --- | --- | --- | --- | --- | --- | --- | --- | --- | --- | --- | --- | --- | --- | --- | --- | --- | --- | --- | --- | --- | --- | --- | --- | --- | --- | --- | --- | --- | --- | --- | --- |
| **Study** | **Participant characteristics** | | | | | | | **LBP characteristics** | | | | | | **Experimental setup/protocol** | | | | | | | | | **Confounding effects control** | | | | | **Statistical information** | | | **Total score** |
|  | **A** | **B** | **C** | **D** | **E** | **F** | **Score** | **G** | **H** | **I** | **J** | **K** | **Score** | **L** | **M** | **N** | **O** | **P** | **Q** | **R** | **S** | **Score** | **T** | **U** | **V** | **W** | **Score** | **X** | **Y** | **Score** |  |
| Cholewicki et al. [21]***** | 1 | 1 | 1 | 1 | 0 | 1 | 83.3 | - | - | - | - | - | - | ½ | 0 | 0 | 1 | 1 | 0 | 1 | ½ | 50 | 0 | 0 | 0 | 0 | 0 | 1 | 0 | 50 | 50 |
| Silfies et al. [77] | 1 | 1 | 1 | 1 | 0 | 1 | 83.3 | - | - | - | - | - | - | 1 | 1 | 1 | 1 | 1 | 1 | 1 | 0 | 87.5 | 0 | 0 | 0 | 0 | 0 | 1 | 0 | 50 | 65 |
| van der Burg et al. [79]**†** | 1 | 1 | 1 | 1 | 0 | 0 | 66.7 | - | - | - | - | - | - | ½ | 0 | 0 | 1 | 1 | ½ | ½ | ½ | 50 | 0 | 0 | 0 | 0 | 0 | 1 | 0 | 50 | 45 |
| Reeves et al. [78] | 1 | 1 | 1 | 1 | 0 | 1 | 83.3 | - | - | - | - | - | - | ½ | 1 | 0 | 1 | 1 | 0 | 1 | 0 | 56.3 | 0 | 0 | 0 | 0 | 0 | 1 | 0 | 50 | 52.5 |
| Cholewicki et al. [80] | 1 | 1 | 1 | 1 | 0 | 0 | 66.7 | - | - | - | - | - | - | ½ | 0 | 0 | 1 | 1 | 0 | 1 | ½ | 50 | 0 | 0 | 0 | 0 | 0 | 1 | 0 | 50 | 45 |
| Lee and Granata [81]**‡** | 1 | 1 | 1 | 1 | 0 | 1 | 83.3 | - | - | - | - | - | - | ½ | 1 | 1 | 1 | 1 | 1 | 1 | 0 | 81.3 | 0 | 0 | 1 | 1 | 50 | 1 | 0 | 50 | 72.5 |
| Lee et al. [82]**†** | 1 | 1 | 1 | 1 | 0 | 1 | 83.3 | - | - | - | - | - | - | ½ | 1 | 1 | 1 | 1 | 1 | 1 | 0 | 81.3 | 0 | 0 | 1 | 1 | 50 | 1 | 0 | 50 | 72.5 |
| Slota et al. [23] | 1 | 1 | 1 | 1 | 0 | 1 | 83.3 | - | - | - | - | - | - | 1 | 1 | 1 | 1 | 1 | 1 | 1 | ½ | 93.8 | 0 | 0 | 1 | 1 | 50 | 1 | 0 | 50 | 77.5 |
| Hendershot & Nussbaum [83] | 1 | 1 | 1 | 1 | 0 | 1 | 83.3 | - | - | - | - | - | - | 1 | 1 | 1 | 1 | 1 | 1 | 1 | 0 | 87.5 | 0 | - | 1 | 1 | 66.7 | 1 | 0 | 50 | 78.9 |
| Hendershot et al. [84] | 1 | 1 | 1 | 1 | 0 | 1 | 83.3 | - | - | - | - | - | - | 1 | 1 | 1 | 1 | 1 | 1 | 1 | ½ | 93.8 | 0 | 0 | 1 | 1 | 50 | 1 | 0 | 50 | 77.5 |
| Barbado et al. [86]***** | 1 | 1 | 1 | 1 | 1 | 0 | 83.3 | - | - | - | - | - | - | 1 | 1 | 1 | 0 | 1 | ½ | 1 | 0 | 68.8 | 0 | - | 0 | 0 | 0 | 1 | 0 | 50 | 60.5 |
| Barbado et al. [85] | 1 | 1 | 1 | 1 | 1 | 1 | 100 | - | - | - | - | - | - | 1 | 1 | 1 | 0 | 1 | 1 | 1 | 0 | 75 | 0 | - | 0 | 0 | 0 | 1 | 0 | 50 | 68.4 |
| Beaudette et al. [87] | 1 | 1 | 1 | 1 | 0 | 0 | 66.7 | - | - | - | - | - | - | ½ | 0 | 1 | 1 | 1 | 1 | 1 | 0 | 68.8 | 0 | 0 | 0 | 0 | 0 | 1 | 0 | 50 | 52.5 |
| Ruggiero et al. [88] | 1 | 1 | 0 | 0 | 0 | 1 | 50 | - | - | - | - | - | - | 0 | 1 | 1 | 1 | 1 | ½ | ½ | 1 | 75 | 0 | - | 0 | 0 | 0 | 1 | 0 | 50 | 52.6 |
| Barbado et al. [28] | 1 | 1 | 1 | 1 | 1 | 1 | 100 | - | - | - | - | - | - | 1 | 0 | 1 | 1 | 1 | ½ | 1 | 0 | 68.8 | 0 | - | 0 | 0 | 0 | 1 | 0 | 50 | 65.8 |
| Barbado et al. [89]***** | 1 | 1 | 1 | 1 | 0 | 1 | 83.3 | - | - | - | - | - | - | 1 | 0 | 1 | 1 | 1 | 1 | 1 | 0 | 75 | 0 | - | 0 | 0 | 0 | 1 | 0 | 50 | 63.2 |
| Glofcheskie & Brown [90] | 1 | 1 | 1 | 1 | 1 | 0 | 83.3 | - | - | - | - | - | - | 0 | 0 | 0 | 1 | 1 | 1 | 1 | 0 | 50 | 0 | - | 0 | 0 | 0 | 1 | 0 | 50 | 52.6 |
| Acasio et al. [91] | 1 | 1 | 1 | 1 | 0 | 1 | 83.3 | - | - | - | - | - | - | 1 | 1 | 1 | 0 | 1 | 1 | 1 | 0 | 75 | 0 | 0 | 1 | 1 | 50 | 1 | 0 | 50 | 70 |
| Williams et al. [92] | 1 | 1 | 1 | 1 | 0 | 1 | 83.3 | - | - | - | - | - | - | ½ | 1 | 1 | 1 | 1 | 1 | 1 | 0 | 81.3 | 0 | 0 | 0 | 0 | 0 | 1 | 0 | 50 | 62.5 |
| Barbado et al. [93]**†** | 1 | 1 | 0 | 1 | 0 | 1 | 66.7 | - | - | - | - | - | - | 1 | 0 | 1 | 0 | 1 | 1 | 1 | 0 | 62.5 | 0 | 0 | 0 | 0 | 0 | 1 | 0 | 50 | 50 |
| Roberts & Vette [25] | 1 | 1 | 1 | 1 | 0 | 1 | 83.3 | - | - | - | - | - | - | ½ | 1 | 1 | 1 | 1 | 1 | 1 | 0 | 81.3 | 0 | - | 0 | 0 | 0 | 1 | 0 | 50 | 65.8 |
| Roberts et al. [27]***** | 1 | 1 | 1 | 1 | 0 | 1 | 83.3 | - | - | - | - | - | - | ½ | 1 | 1 | 1 | 1 | 1 | 1 | 0 | 81.3 | 0 | - | 0 | 0 | 0 | 1 | 0 | 50 | 65.8 |
| Acasio et al. [94]***** | 1 | 1 | 1 | 1 | 0 | 1 | 83.3 | - | - | - | - | - | - | 1 | 1 | 1 | 0 | 1 | 1 | 1 | ½ | 81.3 | 0 | 0 | 1 | 1 | 50 | 1 | 0 | 50 | 72.5 |
| Alshehri et al. [26]***** | 1 | 1 | 1 | 1 | 0 | 1 | 83.3 | - | - | - | - | - | - | 1 | 1 | 1 | 1 | 1 | 1 | 1 | 1 | 100 | 0 | 0 | 1 | 1 | 50 | 1 | 0 | 50 | 80 |
| de Oliveira et al. [95]**‡** | 1 | 1 | 1 | 1 | 0 | 0 | 66.7 | - | - | - | - | - | - | 1 | 1 | 1 | 0 | 1 | 1 | ½ | 0 | 68.8 | 0 | 0 | 1 | 1 | 50 | 1 | 1 | 100 | 67.5 |
|  | **Total score** | | | | | | | | | | | | | | | | | | | | | | | | | | | | | | |
| All studies (*n*=25) | 100 | 100 | 92 | 96 | 16 | 76 | 80 | - | - | - | - | - | - | 72 | 68 | 80 | 76 | 100 | 80 | 94 | 20 | 73.5 | 0 | 0 | 36 | 36 | 18.7 | 100 | 4 | 52 | 63.4 |
| Studies with only IPD (*n*=14) | 100 | 100 | 92.9 | 92.9 | 21.4 | 78.6 | 81 | - | - | - | - | - | - | 67.9 | 71.4 | 78.6 | 85.7 | 100 | 78.6 | 96.4 | 17.9 | 74.6 | 0 | 0 | 28.6 | 28.6 | 15.5 | 100 | 0 | 50 | 63.3 |
| **Abbreviations:** IPD, individual participant data.  **Checklist Items:** **A**, report summary measure of age; **B**, report number or proportion of male/female; **C**, report summary measure of height; **D**, report summary measure of weight; **E**, report information about whether the participants are from a specific participant group; **F**, report information about if pain-free controls/participants had history of LBP; **G**, report type of LBP; **H**, report information about duration of LBP to determine if pain is acute, subacute or chronic; **I**, report pain intensity level using a valid and reliable scale; **J**, report disability level using a valid and reliable scale; **K**, report psychological factors using a valid and reliable scale; **L**, report information about the seat build characteristics; **M**, report information about the visual condition; **N**, use a minimum duration of 30 seconds for each trial (quality); **O**, use at least three repetitions (quality); **P**, report instructions given to participants before recording; **Q**, report information about the sampling rate and applied low pass filter characteristics; **R**, report a clear description about how outcome measures were calculated; **S**, report information about the excluded participants/trials; **T**, statistical adjustment for age (quality); **U**, statistical adjustment for sex (quality); **V**, controlling or statistical adjustment for height (quality); **W**, controlling or statistical adjustment for weight (quality); **X**, report adequate information about the statistical methods used for analysis; **Y**, report information about the power calculation.  *****Studies that were only included in the descriptive analysis but excluded from quantitative/IPD analysis as these studies referred to an already identified IPD set.  **†**IPD were not available as authors did not have access/authorisation to provide the IPD set.  **‡**IPD were not available as authors did not respond to IPD request. | | | | | | | | | | | | | | | | | | | | | | | | | | | | | | | |
