# Supplementary material for: Trunk postural control during unstable sitting among individuals with and without low back pain: A systematic review with an individual participant data meta-analysis
Source: PLoS One. 2024 Jan 24;19(1):e0296968. doi: 10.1371/journal.pone.0296968 (PMC10807788; doi:10.1371/journal.pone.0296968)
Supplement: S19 Table — (DOCX) [file pone.0296968.s020.docx]

| **Table S19.** Reporting/Quality total scores for studies with data from individuals with LBP: published papers versus IPD | | | | | | | | | | | | |
| --- | --- | --- | --- | --- | --- | --- | --- | --- | --- | --- | --- | --- |
| **Study** | **Score (%)** | | | | | | | | | | | |
|  | **Participant characteristics*** | | **LBP characteristics†** | | **Experimental setup/protocol‡** | | **Confounding effects control§** | | **Statistical information**** | | **Total score** | |
|  | **PUB††** | **IPD‡‡** | **PUB††** | **IPD‡‡** | **PUB††** | **IPD‡‡** | **PUB††** | **IPD‡‡** | **PUB††** | **IPD‡‡** | **PUB††** | **IPD‡‡** |
| Radebold et al. [22] | 83.3 | 100 | 80 | 80 | 68.8 | 81.3 | 75 | 100 | 50 | 50 | 74 | 87.5 |
| Reeves et al. [73] | 80 | 100 | 80 | 80 | 75 | 81.3 | 0 | 100 | 50 | 50 | 62.5 | 87 |
| van Dieën et al. [33] | 66.7 | 100 | 20 | 20 | 68.8 | 87.5 | 0 | 100 | 50 | 50 | 46 | 78.3 |
| Willigenburg et al. [31] | 50 | 100 | 60 | 60 | 87.5 | 87.5 | 0 | 100 | 50 | 50 | 56 | 84 |
| Larivière et al. [34] | 83.3 | 100 | 80 | 100 | 75 | 87.5 | 75 | 100 | 50 | 50 | 76 | 92 |
| Sung et al. [19] | 83.3 | 100 | 100 | 100 | 87.5 | 100 | 0 | 100 | 100 | 100 | 76 | 100 |
| Shahvarpour et al. [29] | 83.3 | 100 | 80 | 100 | 75 | 87.5 | 50 | 100 | 50 | 50 | 72 | 92 |
| Shahvarpour et al. [32] | 83.3 | 100 | 80 | 100 | 75 | 87.5 | 50 | 100 | 50 | 50 | 72 | 92 |
| Cyr et al. [30] | 50 | 100 | 60 | 100 | 75 | 81.3 | 0 | 100 | 50 | 50 | 52 | 91.7 |
| van den Hoorn et al. [35] | 83.3 | 100 | 100 | 100 | 100 | 100 | 75 | 100 | 50 | 50 | 88 | 96 |
|  | **Total score** | | | | | | | | | | | |
| All studies (*n*=10) | 74.7 | 100 | 74 | 84 | 78.8 | 88.1 | 32.5 | 100 | 55 | 55 | 67.5 | 90 |
| **Abbreviations:** LBP, low back pain; IPD, individual participant data; PUB, published.  *****This domain assesses the comprehensiveness of reporting about: 1) summary measure of age, 2) number or proportion of male/female, 3) summary measure of height, 4) summary measure of weight, 5) information about whether the participants are from a specific participant group, and 6) information about if pain-free controls/participants had history of LBP.  **†**This domain assesses the comprehensiveness of reporting about: 7) type of LBP, 8) information about duration of LBP to determine if pain is acute, subacute or chronic, 9) pain intensity level using a valid and reliable scale, 10) disability level using a valid and reliable scale, and 11) psychological factors using a valid and reliable scale.  **‡**This domain assesses the comprehensiveness of reporting and methodological quality about: 12) information about the seat build characteristics, 13) information about the visual condition, 14) use a minimum duration of 30 seconds for each trial (quality), 15) use at least three repetitions (quality), 16) instructions given to participants before recording, 17) information about the sampling rate and applied low pass filter characteristics, 18) a clear description about how outcome measures were calculated, and 19) information about the excluded participants/trials.  **§**This domain assesses the methodological quality about controlling and/or statistical adjustment for: 20) age, 21) sex, 22) height, and 23) weight.  ******This domain assesses the comprehensiveness of reporting about: 24) adequate information about the statistical methods used for analysis, and 25) information about the power calculation.  **††**Scores based on the study-level data that are extracted from the published version of papers.  **‡‡**Scores based on the individual-level data that are obtained from the authors. | | | | | | | | | | | | |
