# Supplementary material for: Trunk postural control during unstable sitting among individuals with and without low back pain: A systematic review with an individual participant data meta-analysis
Source: PLoS One. 2024 Jan 24;19(1):e0296968. doi: 10.1371/journal.pone.0296968 (PMC10807788; doi:10.1371/journal.pone.0296968)
Supplement: S21 Table — (DOCX) [file pone.0296968.s022.docx]

| **Table S21.** Excluded participants from the IPD analysis | | | | | | |
| --- | --- | --- | --- | --- | --- | --- |
| **Study** | **Group** | **N** | **Missing information** | | | **<18 years** |
|  |  |  | **Sex** | **Age** | **BMI** |  |
| **Data from individuals with and without LBP** | | | | | | |
| Dieën et al. (2010) | Control | 4 |  | × | × |  |
| Sung et al. (2015) | LBP | 2 | × | × | × |  |
| All |  | 6 | 1 | 4 | 5 |  |
| **Data from only pain-free individuals** | | | | | | |
| Cholewicki et al. (2007) | Pain-free | 1 |  | × | × |  |
| Barbado et al. (2016b) | Pain-free | 9 |  | × |  | × |
| Glofcheskie and Brown (2017) | Pain-free | 7 |  | × | × |  |
| All |  | 17 |  | 9 | 8 | 8 |
| **Not excluded participants*** | | | | | | |
| Slota et al. (2008) | Pain-free | 21 | × | × | × |  |
| Ruggiero et al. (2016) | Pain-free | 24 |  | × | × |  |
| All |  | 45 | 21 | 45 | 29 |  |
| **Abbreviations:** IPD, individual participant data; N, number; BMI, body mass index; LBP, low back pain.  *****Statistical adjustments of sex, age, and BMI variables were not performed in the IPD analysis for two studies with data from only pain-free individuals as these studies did not report at least one demographic variable for all participants. Therefore, we did not exclude these two studies to avoid losing the large number of participants and also they were not studies with data from individuals with versus without LBP where demographic information are critical. | | | | | | |
