# Supplementary material for: Trunk postural control during unstable sitting among individuals with and without low back pain: A systematic review with an individual participant data meta-analysis
Source: PLoS One. 2024 Jan 24;19(1):e0296968. doi: 10.1371/journal.pone.0296968 (PMC10807788; doi:10.1371/journal.pone.0296968)
Supplement: S22 Table — (DOCX) [file pone.0296968.s023.docx]

| **Table S22.** Included and excluded levels of the seat difficulty in the IPD analysis | | |
| --- | --- | --- |
| **Study** | **Seat apparatus**  ***Seat difficulty levels*** | **Included in the IPD analysis** |
| Radebold et al. [22] | Hemisphere  *Level 0: flat surface*  *Level 1: radius 25 cm*  ***Level 2: radius 22 cm***  *Level 3: radius 11 cm* | Level 2 was selected as it is the most closet level to hemisphere characteristics that were used in other studies. |
| Silfies et al. [77] | Hemisphere  *Level 0: flat surface*  *Level 1: radius 25 cm; height 8.2 cm*  ***Level 2: radius 25 cm; height 13.2 cm***  *Level 3: radius 25 cm; height 18.2 cm* | Level 2 was selected. |
| Acasio et al. [91] | Springs  *Level 1: R_spring_ 100%*  *Level 2: R_spring_ 75%*  ***Level 3: R_spring_ 60%***  *Level 4: R_spring_ 45%* | Level 3 was selected as it is the most closet level to springs characteristics that were used in other studies. |
| Williams et al. [92] | Hemisphere  *L1: radius 25 cm; height NA*  ***L2: radius 20 cm; height NA***  *L3: radius 15 cm; height NA*  *L4: radius 13 cm; height NA*  *L5: radius 11 cm; height NA* | Level 2 was selected as it is the most closet level to hemisphere characteristics that were used in other studies. |
| **Abbreviation:** IPD, individual participant data; R_spring_, distance (radius) of springs from the pivot in percentage; NA, not available. | | |
