# Supplementary material for: Trunk postural control during unstable sitting among individuals with and without low back pain: A systematic review with an individual participant data meta-analysis
Source: PLoS One. 2024 Jan 24;19(1):e0296968. doi: 10.1371/journal.pone.0296968 (PMC10807788; doi:10.1371/journal.pone.0296968)
Supplement: S23 Table — (DOCX) [file pone.0296968.s024.docx]

| **Table S23.** A two-stage IPD meta-regression of associations between LBP intensity or disability and trunk postural control | | | | | | | |
| --- | --- | --- | --- | --- | --- | --- | --- |
| **Outcome** | | **VAS/NPRS** | | | **RMDQ** | | |
|  |  | **Coef. (SE)** | **I^2^_res_** | ***P*-value** | **Coef. (SE)** | **I^2^_res_** | ***P*-value** |
| RMS_displ_ | EO-AP | −0.17 (0.77) | 29.64 | 0.860 | Insufficient Observations | | |
|  | EO-ML | −0.94 (1.39) | 78.33 | 0.622 | Insufficient Observations | | |
|  | EC-AP | 0.12 (0.11) | 33.06 | 0.324 | −0.01 (0.02) | 43.55 | 0.873 |
|  | EC-ML | 0.04 (0.13) | 53.46 | 0.770 | −0.01 (0.03) | 70.27 | 0.659 |
| M_vel_ | EO-AP | Insufficient Observations | | | Insufficient Observations | | |
|  | EO-ML | Insufficient Observations | | | Insufficient Observations | | |
|  | EC-AP | −0.04 (0.11) | 0.00 | 0.759 | −0.01 (0.03) | 39.08 | 0.771 |
|  | EC-ML | 0.07 (0.10) | 0.00 | 0.561 | −0.02 (0.02) | 0.00 | 0.489 |
| Range | EO-AP | Insufficient Observations | | | Insufficient Observations | | |
|  | EO-ML | Insufficient Observations | | | Insufficient Observations | | |
|  | EC-AP | 0.37 (0.34) | 15.59 | 0.350 | −0.04 (0.04) | 0.00 | 0.429 |
|  | EC-ML | 0.25 (0.33) | 22.80 | 0.496 | −0.05 (0.04) | 0.00 | 0.346 |
| MPF | EO-AP | Insufficient Observations | | | Insufficient Observations | | |
|  | EO-ML | Insufficient Observations | | | Insufficient Observations | | |
|  | EC-AP | 0.4^e-2^ (0.3^e-2^) | 0.00 | 0.319 | −0.6^e-3^ (0.7^e-3^) | 0.00 | 0.442 |
|  | EC-ML | 0.4^e-2^ (0.3^e-2^) | 0.00 | 0.342 | −0.3^e-3^ (0.7^e-3^) | 0.00 | 0.721 |
| **Abbreviations:** IPD, individual participant data; LBP, low back pain; VAS, visual analogue scale; NPRS, numeric pain rating scale; RMDQ, Roland-Morris disability questionnaire; Coef., coefficient; SE, standard error; I^2^_res_, residual heterogeneity statistic as a percentage; RMS_displ_, root mean square displacement; M_vel_, mean velocity; MPF, mean power frequency; EO, eyes open; EC, eyes closed; AP, anteroposterior; ML, mediolateral.  *P*-values of statistically significant regression coefficients (*P*<0.05) are printed bold. | | | | | | | |
