# Supplementary material for: Trunk postural control during unstable sitting among individuals with and without low back pain: A systematic review with an individual participant data meta-analysis
Source: PLoS One. 2024 Jan 24;19(1):e0296968. doi: 10.1371/journal.pone.0296968 (PMC10807788; doi:10.1371/journal.pone.0296968)
Supplement: S24 Table — (DOCX) [file pone.0296968.s025.docx]

| **Table S24.** A two-stage IPD meta-regression of associations between pain catastrophizing or fear-avoidance beliefs and trunk postural control | | | | | | | | | | | | | |
| --- | --- | --- | --- | --- | --- | --- | --- | --- | --- | --- | --- | --- | --- |
| **Outcome** | | **PCS** | | | **FABQ-PA** | | | **FABQ-W** | | | **FABQ** | | |
|  |  | **Coef. (SE)** | **I^2^_res_** | ***P*-value** | **Coef. (SE)** | **I^2^_res_** | ***P*-value** | **Coef. (SE)** | **I^2^_res_** | ***P*-value** | **Coef. (SE)** | **I^2^_res_** | ***P*-value** |
| RMS_displ_ | EO-AP | Insufficient Observations | | | −0.39 (0.28) | 79.10 | 0.393 | −0.02 (0.07) | 24.23 | 0.788 | −0.07 (0.04) | 85.18 | 0.355 |
|  | EO-ML | Insufficient Observations | | | −0.36 (0.25) | 86.83 | 0.390 | −0.02 (0.05) | 22.26 | 0.752 | −0.05 (0.03) | 83.13 | 0.329 |
|  | EC-AP | 0.01 (0.01) | 0.00 | 0.704 | −0.01 (0.02) | 0.00 | 0.749 | 0.01 (0.03) | 71.05 | 0.794 | 0.1^e-3^ (0.02) | 66.38 | 0.997 |
|  | EC-ML | 0.01 (0.01) | 0.00 | 0.352 | 0.2^e-3^ (0.03) | 0.00 | 0.995 | 0.02 (0.01) | 27.21 | 0.346 | 0.01 (0.02) | 69.11 | 0.745 |
| M_vel_ | EO-AP | Insufficient Observations | | | Insufficient Observations | | | Insufficient Observations | | | Insufficient Observations | | |
|  | EO-ML | Insufficient Observations | | | Insufficient Observations | | | Insufficient Observations | | | Insufficient Observations | | |
|  | EC-AP | 0.01 (0.01) | 0.00 | 0.242 | −0.01 (0.03) | 0.00 | 0.759 | 0.04 (0.03) | 85.69 | 0.422 | 0.02 (0.02) | 82.19 | 0.487 |
|  | EC-ML | 0.01 (0.01) | 0.00 | 0.244 | −0.03 (0.03) | 0.00 | 0.404 | 0.03 (0.02) | 76.95 | 0.391 | 0.02 (0.02) | 60.19 | 0.413 |
| Range | EO-AP | Insufficient Observations | | | Insufficient Observations | | | Insufficient Observations | | | Insufficient Observations | | |
|  | EO-ML | Insufficient Observations | | | Insufficient Observations | | | Insufficient Observations | | | Insufficient Observations | | |
|  | EC-AP | 0.6^e-3^ (0.01) | 0.00 | 0.965 | Convergence Not Achieved | | | 0.01 (0.09) | 72.66 | 0.906 | 0.4^e-2^ (0.06) | 47.49 | 0.958 |
|  | EC-ML | 0.01 (0.01) | 0.00 | 0.593 | 0.01 (0.08) | 0.00 | 0.933 | 0.05 (0.06) | 35.08 | 0.532 | 0.03 (0.05) | 27.78 | 0.672 |
| MPF | EO-AP | Insufficient Observations | | | Insufficient Observations | | | Insufficient Observations | | | Insufficient Observations | | |
|  | EO-ML | Insufficient Observations | | | Insufficient Observations | | | Insufficient Observations | | | Insufficient Observations | | |
|  | EC-AP | −0.7^e-4^ (0.2^e-3^) | 42.26 | 0.787 | −0.2^e-2^ (0.1^e-2^) | 0.00 | 0.407 | Insufficient Observations | | | Insufficient Observations | | |
|  | EC-ML | −0.3^e-4^ (0.1^e-3^) | 0.00 | 0.840 | −0.2^e-2^ (0.2^e-2^) | 0.00 | 0.399 | Insufficient Observations | | | Insufficient Observations | | |
| **Abbreviations:** IPD, individual participant data; PCS, pain catastrophizing scale; FABQ-PA, fear-avoidance beliefs questionnaire - physical activity; FABQ-W, fear-avoidance beliefs questionnaire - work; FABQ, fear-avoidance beliefs questionnaire; Coef., coefficient; SE, standard error; I^2^_res_, residual heterogeneity statistic as a percentage; RMS_displ_, root mean square displacement; M_vel_, mean velocity; MPF, mean power frequency; EO, eyes open; EC, eyes closed; AP, anteroposterior; ML, mediolateral.  *P*-values of statistically significant regression coefficients (*P*<0.05) are printed bold. | | | | | | | | | | | | | |
