# Supplementary material for: Trunk postural control during unstable sitting among individuals with and without low back pain: A systematic review with an individual participant data meta-analysis
Source: PLoS One. 2024 Jan 24;19(1):e0296968. doi: 10.1371/journal.pone.0296968 (PMC10807788; doi:10.1371/journal.pone.0296968)
Supplement: S25 Table — (DOCX) [file pone.0296968.s026.docx]

| **Table S25.** Individual IPD analysis of associations between LBP intensity or disability and RMS_displ_ for each study | | | | | |
| --- | --- | --- | --- | --- | --- |
| **Outcome** | **Study** | **VAS/NPRS** | | **RMDQ** | |
|  |  | **Coef. (SE)** | ***P*-value** | **Coef. (SE)** | ***P*-value** |
| EO-AP | Larivière et al. [34] | - | - | - | - |
|  | Sung et al. [19] | −0.04 (0.10) | 0.656 | - | - |
|  | Shahvarpour et al. [29] | - | - | - | - |
|  | Cyr et al. [30] | 1.78 (1.55) | 0.253 | 1.27 (0.57) | **0.025** |
|  | Shahvarpour et al. [32] | - | - | - | - |
|  | van den Hoorn et al. [35] | 0.10 (0.09) | 0.276 | 0.01 (0.03) | 0.767 |
| EO-ML | Larivière et al. [34] | - | - | - | - |
|  | Sung et al. [19] | −0.04 (0.12) | 0.741 | - | - |
|  | Shahvarpour et al. [29] | - | - | - | - |
|  | Cyr et al. [30] | 2.33 (1.10) | **0.035** | 1.24 (0.40) | **0.002** |
|  | Shahvarpour et al. [32] | - | - | - | - |
|  | van den Hoorn et al. [35] | 0.04 (0.08) | 0.580 | 0.03 (0.03) | 0.315 |
| EC-AP | Larivière et al. [34] | −0.25 (0.21) | 0.236 | 0.04 (0.14) | 0.777 |
|  | Sung et al. [19] | −0.04 (0.34) | 0.902 | - | - |
|  | Shahvarpour et al. [29] | 0.33 (0.22) | 0.134 | −0.02 (0.08) | 0.829 |
|  | Cyr et al. [30] | 10.55 (5.69) | 0.064 | 5.05 (2.28) | **0.026** |
|  | Shahvarpour et al. [32] | 0.17 (0.18) | 0.346 | 0.01 (0.10) | 0.946 |
|  | van den Hoorn et al. [35] | 0.23 (0.21) | 0.287 | −0.05 (0.08) | 0.527 |
| EC-ML | Larivière et al. [34] | −0.26 (0.22) | 0.234 | −0.02 (0.14) | 0.906 |
|  | Sung et al. [19] | 0.22 (0.38) | 0.572 | - | - |
|  | Shahvarpour et al. [29] | 0.10 (0.23) | 0.656 | −0.09 (0.08) | 0.257 |
|  | Cyr et al. [30] | 11.73 (4.93) | 0.017 | 5.80 (1.85) | **0.002** |
|  | Shahvarpour et al. [32] | 0.22 (0.18) | 0.211 | 0.02 (0.09) | 0.803 |
|  | van den Hoorn et al. [35] | 0.10 (0.18) | 0.557 | −0.04 (0.07) | 0.531 |
| **Abbreviations:** IPD, individual participant data; LBP, low back pain; RMS_displ_, root mean square displacement; VAS, visual analogue scale; NPRS, numeric pain rating scale; RMDQ, Roland-Morris disability questionnaire; Coef., coefficient; SE, standard error; EO, eyes open; EC, eyes closed; AP, anteroposterior; ML, mediolateral.  *P*-values of statistically significant regression coefficients (*P*<0.05) are printed bold. | | | | | |
