# Supplementary material for: Trunk postural control during unstable sitting among individuals with and without low back pain: A systematic review with an individual participant data meta-analysis
Source: PLoS One. 2024 Jan 24;19(1):e0296968. doi: 10.1371/journal.pone.0296968 (PMC10807788; doi:10.1371/journal.pone.0296968)
Supplement: S26 Table — (DOCX) [file pone.0296968.s027.docx]

| **Table S26.** Individual IPD analysis of associations between pain catastrophizing or fear-avoidance beliefs and RMS_displ_ for each study | | | | | | | | | |
| --- | --- | --- | --- | --- | --- | --- | --- | --- | --- |
| **Outcome** | **Study** | **PCS** | | **FABQ-PA** | | **FABQ-W** | | **FABQ** | |
|  |  | **Coef. (SE)** | ***P*-value** | **Coef. (SE)** | ***P*-value** | **Coef. (SE)** | ***P*-value** | **Coef. (SE)** | ***P*-value** |
| EO-AP | Larivière et al. [34] | - | - | - | - | - | - | - | - |
|  | Sung et al. [19] | 0.01 (0.01) | 0.376 | 0.01 (0.02) | 0.402 | 0.2^e-2^ (0.01) | 0.712 | 0.01 (0.01) | 0.547 |
|  | Shahvarpour et al. [29] | - | - | - | - | - | - | - | - |
|  | Cyr et al. [30] | - | - | 1.29 (0.56) | **0.021** | 0.37 (0.30) | 0.226 | 0.45 (0.17) | **0.006** |
|  | Shahvarpour et al. [32] | - | - | - | - | - | - | - | - |
|  | van den Hoorn et al. [35] | 0.02 (0.02) | 0.275 | −0.02 (0.03) | 0.412 | 0.02 (0.02) | 0.289 | 0.01 (0.01) | 0.582 |
| EO-ML | Larivière et al. [34] | - | - | - | - | - | - | - | - |
|  | Sung et al. [19] | 0.1^e-2^ (0.02) | 0.941 | −0.01 (0.02 | 0.798 | −0.01 (0.01) | 0.631 | −0.4^e-2^ (0.01) | 0.671 |
|  | Shahvarpour et al. [29] | - | - | - | - | - | - | - | - |
|  | Cyr et al. [30] | - | - | 1.14 (0.42) | **0.007** | 0.29 (0.24) | 0.233 | 0.37 (0.13) | **0.005** |
|  | Shahvarpour et al. [32] | - | - | - | - | - | - | - | - |
|  | van den Hoorn et al. [35] | 0.02 (0.01) | 0.094 | 0.02 (0.03) | 0.560 | 0.01 (0.01) | 0.369 | 0.01 (0.01) | 0.274 |
| EC-AP | Larivière et al. [34] | 0.01 (0.04) | 0.762 | - | - | - | - | - | - |
|  | Sung et al. [19] | −0.4^e-2^ (0.04) | 0.922 | 0.08 (0.06) | 0.168 | −0.05 (0.04) | 0.230 | −0.01 (0.03) | 0.760 |
|  | Shahvarpour et al. [29] | 0.04 (0.03) | 0.103 | 0.04 (0.06) | 0.478 | - | - | - | - |
|  | Cyr et al. [30] | - | - | 3.49 (2.54) | 0.169 | 0.55 (1.28) | 0.666 | 1.27 (0.78) | 0.103 |
|  | Shahvarpour et al. [32] | 0.06 (0.03) | 0.058 | 0.02 (0.06) | 0.691 | 0.09 (0.02) | **0.000** | 0.06 (0.02) | **0.001** |
|  | van den Hoorn et al. [35] | 0.07 (0.04) | 0.065 | 0.02 (0.07) | 0.830 | 0.05 (0.04) | 0.211 | 0.04 (0.03) | 0.201 |
| EC-ML | Larivière et al. [34] | 0.01 (0.04) | 0.842 | - | - | - | - | - | - |
|  | Sung et al. [19] | 0.01 (0.06) | 0.830 | 0.03 (0.07) | 0.684 | −0.04 (0.05) | 0.370 | −0.02 (0.04) | 0.587 |
|  | Shahvarpour et al. [29] | 0.04 (0.03) | 0.126 | 0.02 (0.06) | 0.718 | - | - | - | - |
|  | Cyr et al. [30] | - | - | 3.71 (2.31) | 0.108 | 0.97 (1.17) | 0.409 | 1.45 (0.68) | **0.032** |
|  | Shahvarpour et al. [32] | 0.06 (0.03) | **0.045** | −0.1^e-2^ (0.06) | 0.981 | 0.07 (0.02) | **0.002** | 0.05 (0.02) | **0.016** |
|  | van den Hoorn et al. [35] | 0.1^e-2^ (0.03) | 0.957 | 0.04 (0.06) | 0.513 | −0.01 (0.03) | 0.705 | 0.1^e-2^ (0.03) | 0.958 |
| **Abbreviations:** IPD, individual participant data; RMS_displ_, root mean square displacement; PCS, pain catastrophizing scale; FABQ-PA, fear-avoidance beliefs questionnaire - physical activity; FABQ-W, fear-avoidance beliefs questionnaire - work; FABQ, fear-avoidance beliefs questionnaire; Coef., coefficient; SE, standard error; EO, eyes open; EC, eyes closed; AP, anteroposterior; ML, mediolateral.  *P*-values of statistically significant regression coefficients (*P*<0.05) are printed bold. | | | | | | | | | |
