# Supplementary material for: Trunk postural control during unstable sitting among individuals with and without low back pain: A systematic review with an individual participant data meta-analysis
Source: PLoS One. 2024 Jan 24;19(1):e0296968. doi: 10.1371/journal.pone.0296968 (PMC10807788; doi:10.1371/journal.pone.0296968)
Supplement: S27 Table — (DOCX) [file pone.0296968.s028.docx]

| **Table S27.** Individual IPD analysis of associations between LBP intensity or disability and M_vel_ for each study | | | | | |
| --- | --- | --- | --- | --- | --- |
| **Outcome** | **Study** | **VAS/NPRS** | | **RMDQ** | |
|  |  | **Coef. (SE)** | ***P*-value** | **Coef. (SE)** | ***P*-value** |
| EO-AP | Reeves et al. [73] | - | - | - | - |
|  | Larivière et al. [34] | - | - | - | - |
|  | Sung et al. [19] | −0.35 (0.15) | **0.019** | - | - |
|  | Shahvarpour et al. [29] | - | - | - | - |
|  | Shahvarpour et al. [32] | - | - | - | - |
|  | van den Hoorn et al. [35] | 0.11 (0.10) | 0.271 | −0.02 (0.04) | 0.525 |
| EO-ML | Reeves et al. [73] | - | - | - | - |
|  | Larivière et al. [34] | - | - | - | - |
|  | Sung et al. [19] | −0.34 (0.19) | 0.080 | - | - |
|  | Shahvarpour et al. [29] | - | - | - | - |
|  | Shahvarpour et al. [32] | - | - | - | - |
|  | van den Hoorn et al. [35] | 0.07 (0.11) | 0.504 | −0.04 (0.04) | 0.416 |
| EC-AP | Reeves et al. [73] | −0.83 (0.46) | 0.071 | - | - |
|  | Larivière et al. [34] | 0.56 (0.33) | 0.090 | 0.16 (0.22) | 0.452 |
|  | Sung et al. [19] | 0.16 (0.30) | 0.586 | - | - |
|  | Shahvarpour et al. [29] | 0.25 (0.31) | 0.422 | 0.01 (0.11) | 0.928 |
|  | Shahvarpour et al. [32] | 0.05 (0.22) | 0.807 | 0.09 (0.12) | 0.436 |
|  | van den Hoorn et al. [35] | 0.06 (0.27) | 0.838 | −0.16 (0.11) | 0.135 |
| EC-ML | Reeves et al. [73] | −0.75 (0.57) | 0.188 | - | - |
|  | Larivière et al. [34] | −0.45 (0.30) | 0.137 | 0.11 (0.20) | 0.577 |
|  | Sung et al. [19] | −0.09 (0.29) | 0.741 | - | - |
|  | Shahvarpour et al. [29] | 0.02 (0.29) | 0.950 | −0.07 (0.10) | 0.505 |
|  | Shahvarpour et al. [32] | 0.7^e-3^ (0.23) | 0.997 | 0.10 (0.12) | 0.395 |
|  | van den Hoorn et al. [35] | −0.02 (0.23) | 0.927 | −0.11 (0.09) | 0.218 |
| **Abbreviations:** IPD, individual participant data; LBP, low back pain; M_vel_, mean velocity; VAS, visual analogue scale; NPRS, numeric pain rating scale; RMDQ, Roland-Morris disability questionnaire; Coef., coefficient; SE, standard error; EO, eyes open; EC, eyes closed; AP, anteroposterior; ML, mediolateral.  *P*-values of statistically significant regression coefficients (*P*<0.05) are printed bold. | | | | | |
