# Supplementary material for: Trunk postural control during unstable sitting among individuals with and without low back pain: A systematic review with an individual participant data meta-analysis
Source: PLoS One. 2024 Jan 24;19(1):e0296968. doi: 10.1371/journal.pone.0296968 (PMC10807788; doi:10.1371/journal.pone.0296968)
Supplement: S28 Table — (DOCX) [file pone.0296968.s029.docx]

| **Table S28.** Individual IPD analysis of associations between pain catastrophizing or fear-avoidance beliefs and M_vel_ for each study | | | | | | | | | |
| --- | --- | --- | --- | --- | --- | --- | --- | --- | --- |
| **Outcome** | **Study** | **PCS** | | **FABQ-PA** | | **FABQ-W** | | **FABQ** | |
|  |  | **Coef. (SE)** | ***P*-value** | **Coef. (SE)** | ***P*-value** | **Coef. (SE)** | ***P*-value** | **Coef. (SE)** | ***P*-value** |
| EO-AP | Reeves et al. [73] | - | - | - | - | - | - | - | - |
|  | Larivière et al. [34] | - | - | - | - | - | - | - | - |
|  | Sung et al. [19] | 0.03 (0.02) | 0.250 | 0.04 (0.03) | 0.131 | 0.05 (0.02) | **0.003** | 0.04 (0.01) | **0.003** |
|  | Shahvarpour et al. [29] | - | - | - | - | - | - | - | - |
|  | Shahvarpour et al. [32] | - | - | - | - | - | - | - | - |
|  | van den Hoorn et al. [35] | 0.1^e-2^ (0.02) | 0.947 | −0.2^e-2^ (0.03) | 0.937 | 0.02 (0.02) | 0.279 | 0.01 (0.02) | 0.356 |
| EO-ML | Reeves et al. [73] | - | - | - | - | - | - | - | - |
|  | Larivière et al. [34] | - | - | - | - | - | - | - | - |
|  | Sung et al. [19] | 0.03 (0.03) | 0.365 | 0.03 (0.04) | 0.366 | 0.07 (0.02) | **0.001** | 0.05 (0.02) | **0.005** |
|  | Shahvarpour et al. [29] | - | - | - | - | - | - | - | - |
|  | Shahvarpour et al. [32] | - | - | - | - | - | - | - | - |
|  | van den Hoorn et al. [35] | −0.01 (0.02) | 0.738 | 0.01 (0.04) | 0.874 | 0.01 (0.02) | 0.678 | 0.01 (0.02) | 0.609 |
| EC-AP | Reeves et al. [73] | - | - | - | - | - | - | - | - |
|  | Larivière et al. [34] | 0.05 (0.06) | 0.382 | - | - | - | - | - | - |
|  | Sung et al. [19] | −0.03 (0.04) | 0.448 | 0.02 (0.05) | 0.713 | −0.09 (0.03) | **0.004** | −0.05 (0.03) | 0.053 |
|  | Shahvarpour et al. [29] | 0.03 (0.04) | 0.418 | −0.01 (0.08) | 0.875 | - | - | - | - |
|  | Shahvarpour et al. [32] | 0.06 (0.04) | 0.118 | 0.02 (0.08) | 0.770 | 0.10 (0.03) | **0.000** | 0.07 (0.02) | **0.002** |
|  | van den Hoorn et al. [35] | −0.02 (0.05) | 0.660 | 0.02 (0.09) | 0.861 | -0.06 (0.05) | 0.243 | -0.03 (0.04) | 0.437 |
| EC-ML | Reeves et al. [73] | - | - | - | - | - | - | - | - |
|  | Larivière et al. [34] | 0.04 (0.06) | 0.531 | - | - | - | - | - | - |
|  | Sung et al. [19] | −0.01 (0.04) | 0.890 | 0.05 (0.05) | 0.318 | −0.06 (0.03) | 0.076 | -0.02 (0.03) | 0.418 |
|  | Shahvarpour et al. [29] | 0.03 (0.03) | 0.393 | −0.05 (0.07) | 0.506 | - | - | - | - |
|  | Shahvarpour et al. [32] | 0.06 (0.04) | 0.119 | −0.3^e-3^ (0.08) | 0.997 | 0.10 (0.03) | **0.001** | 0.07 (0.02) | **0.008** |
|  | van den Hoorn et al. [35] | −0.04 (0.04) | 0.327 | 0.02 (0.08) | 0.771 | −0.04 (0.04) | 0.348 | -0.02 (0.04) | 0.566 |
| **Abbreviations:** IPD, individual participant data; M_vel_, mean velocity; PCS, pain catastrophizing scale; FABQ-PA, fear-avoidance beliefs questionnaire - physical activity; FABQ-W, fear-avoidance beliefs questionnaire - work; FABQ, fear-avoidance beliefs questionnaire; Coef., coefficient; SE, standard error; EO, eyes open; EC, eyes closed; AP, anteroposterior; ML, mediolateral.  *P*-values of statistically significant regression coefficients (*P*<0.05) are printed bold. | | | | | | | | | |
