# Supplementary material for: Trunk postural control during unstable sitting among individuals with and without low back pain: A systematic review with an individual participant data meta-analysis
Source: PLoS One. 2024 Jan 24;19(1):e0296968. doi: 10.1371/journal.pone.0296968 (PMC10807788; doi:10.1371/journal.pone.0296968)
Supplement: S29 Table — (DOCX) [file pone.0296968.s030.docx]

| **Table S29.** Individual IPD analysis of associations between LBP intensity or disability and range for each study | | | | | |
| --- | --- | --- | --- | --- | --- |
| **Outcome** | **Study** | **VAS/NPRS** | | **RMDQ** | |
|  |  | **Coef. (SE)** | ***P*-value** | **Coef. (SE)** | ***P*-value** |
| EO-AP | Larivière et al. [34] | - | - | - | - |
|  | Sung et al. [19] | −0.56 (0.61) | 0.356 | - | - |
|  | Shahvarpour et al. [29] | - | - | - | - |
|  | Shahvarpour et al. [32] | - | - | - | - |
|  | van den Hoorn et al. [35] | 0.59 (0.42) | 0.161 | 0.04 (0.17) | 0.796 |
| EO-ML | Larivière et al. [34] | - | - | - | - |
|  | Sung et al. [19] | −0.46 (0.57) | 0.427 | - | - |
|  | Shahvarpour et al. [29] | - | - | - | - |
|  | Shahvarpour et al. [32] | - | - | - | - |
|  | van den Hoorn et al. [35] | 0.11 (0.36) | 0.758 | 0.15 (0.14) | 0.297 |
| EC-AP | Larivière et al. [34] | −0.80 (0.54) | 0.137 | 0.28 (0.35) | 0.417 |
|  | Sung et al. [19] | 1.34 (1.64) | 0.415 | - | - |
|  | Shahvarpour et al. [29] | 0.64 (0.61) | 0.290 | −0.17 (0.22) | 0.438 |
|  | Shahvarpour et al. [32] | 0.63 (0.55) | 0.253 | 0.10 (0.29) | 0.719 |
|  | van den Hoorn et al. [35] | 0.86 (0.96) | 0.371 | −0.36 (0.38) | 0.345 |
| EC-ML | Larivière et al. [34] | −0.70 (0.49) | 0.151 | 0.18 (0.32) | 0.570 |
|  | Sung et al. [19] | 1.86 (1.87) | 0.320 | - | - |
|  | Shahvarpour et al. [29] | 0.42 (0.66) | 0.521 | −0.27 (0.23) | 0.245 |
|  | Shahvarpour et al. [32] | 0.58 (0.50) | 0.252 | 0.12 (0.27) | 0.674 |
|  | van den Hoorn et al. [35] | 0.45 (0.87) | 0.603 | −0.36 (0.34) | 0.297 |
| **Abbreviations:** IPD, individual participant data; LBP, low back pain; VAS, visual analogue scale; NPRS, numeric pain rating scale; RMDQ, Roland-Morris disability questionnaire; Coef., coefficient; SE, standard error; EO, eyes open; EC, eyes closed; AP, anteroposterior; ML, mediolateral.  *P*-values of statistically significant regression coefficients (*P*<0.05) are printed bold. | | | | | |
