# Supplementary material for: Trunk postural control during unstable sitting among individuals with and without low back pain: A systematic review with an individual participant data meta-analysis
Source: PLoS One. 2024 Jan 24;19(1):e0296968. doi: 10.1371/journal.pone.0296968 (PMC10807788; doi:10.1371/journal.pone.0296968)
Supplement: S30 Table — (DOCX) [file pone.0296968.s031.docx]

| **Table S30.** Individual IPD analysis of associations between pain catastrophizing or fear-avoidance beliefs and range for each study | | | | | | | | | |
| --- | --- | --- | --- | --- | --- | --- | --- | --- | --- |
| **Outcome** | **Study** | **PCS** | | **FABQ-PA** | | **FABQ-W** | | **FABQ** | |
|  |  | **Coef. (SE)** | ***P*-value** | **Coef. (SE)** | ***P*-value** | **Coef. (SE)** | ***P*-value** | **Coef. (SE)** | ***P*-value** |
| EO-AP | Larivière et al. [34] | - | - | - | - | - | - | - | - |
|  | Sung et al. [19] | 0.12 (0.09) | 0.167 | 0.14 (0.11) | 0.199 | 0.05 (0.07) | 0.470 | 0.06 (0.06) | 0.291 |
|  | Shahvarpour et al. [29] | - | - | - | - | - | - | - | - |
|  | Shahvarpour et al. [32] | - | - | - | - | - | - | - | - |
|  | van den Hoorn et al. [35] | 0.09 (0.08) | 0.263 | −0.14 (0.14) | 0.337 | 0.12 (0.08) | 0.156 | 0.05 (0.07) | 0.486 |
| EO-ML | Larivière et al. [34] | - | - | - | - | - | - | - | - |
|  | Sung et al. [19] | 0.04 (0.08) | 0.656 | 0.03 (0.10) | 0.807 | −0.7^e-2^ (0.07) | 0.922 | 0.006 (0.05) | 0.913 |
|  | Shahvarpour et al. [29] | - | - | - | - | - | - | - | - |
|  | Shahvarpour et al. [32] | - | - | - | - | - | - | - | - |
|  | van den Hoorn et al. [35] | 0.12 (0.07) | 0.070 | 0.12 (0.12) | 0.343 | 0.09 (0.07) | 0.187 | 0.09 (0.06) | 0.114 |
| EC-AP | Larivière et al. [34] | 0.07 (0.10) | 0.505 | - | - | - | - | - | - |
|  | Sung et al. [19] | −0.03 (0.22) | 0.907 | 0.30 (0.30) | 0.313 | −0.20 (0.19) | 0.308 | −0.06 (0.15) | 0.686 |
|  | Shahvarpour et al. [29] | 0.10 (0.07) | 0.143 | 0.07 (0.15) | 0.659 | - | - | - | - |
|  | Shahvarpour et al. [32] | 0.18 (0.09) | **0.043** | 0.10 (0.18) | 0.573 | 0.24 (0.07) | **0.001** | 0.17 (0.06) | **0.004** |
|  | van den Hoorn et al. [35] | 0.23 (0.18) | 0.194 | 0.05 (0.33) | 0.874 | 0.20 (0.19) | 0.282 | 0.17 (0.15) | 0.266 |
| EC-ML | Larivière et al. [34] | 0.07 (0.09) | 0.433 | - | - | - | - | - | - |
|  | Sung et al. [19] | −0.05 (0.26) | 0.861 | 0.07 (0.34) | 0.830 | −0.21 (0.22) | 0.347 | −0.13 (0.18) | 0.475 |
|  | Shahvarpour et al. [29] | 0.08 (0.08) | 0.326 | 0.03 (0.16) | 0.836 | - | - | - | - |
|  | Shahvarpour et al. [32] | 0.17 (0.09) | **0.042** | 0.03 (0.18) | 0.846 | 0.19 (0.07) | **0.006** | 0.13 (0.06) | **0.023** |
|  | van den Hoorn et al. [35] | −0.04 (0.16) | 0.790 | 0.16 (0.30) | 0.582 | −0.02 (0.17) | 0.922 | 0.04 (0.14) | 0.794 |
| **Abbreviations:** IPD, individual participant data; PCS, pain catastrophizing scale; FABQ-PA, fear-avoidance beliefs questionnaire - physical activity; FABQ-W, fear-avoidance beliefs questionnaire - work; FABQ, fear-avoidance beliefs questionnaire; Coef., coefficient; SE, standard error; EO, eyes open; EC, eyes closed; AP, anteroposterior; ML, mediolateral.  *P*-values of statistically significant regression coefficients (*P*<0.05) are printed bold. | | | | | | | | | |
