# Supplementary material for: Trunk postural control during unstable sitting among individuals with and without low back pain: A systematic review with an individual participant data meta-analysis
Source: PLoS One. 2024 Jan 24;19(1):e0296968. doi: 10.1371/journal.pone.0296968 (PMC10807788; doi:10.1371/journal.pone.0296968)
Supplement: S31 Table — (DOCX) [file pone.0296968.s032.docx]

| **Table S31.** Individual IPD analysis of associations between LBP intensity or disability and MPF for each study | | | | | |
| --- | --- | --- | --- | --- | --- |
| **Outcome** | **Study** | **VAS/NPRS** | | **RMDQ** | |
|  |  | **Coef. (SE)** | ***P*-value** | **Coef. (SE)** | ***P*-value** |
| EO-AP | Larivière et al. [34] | - | - | - | - |
|  | Shahvarpour et al. [29] | - | - | - | - |
|  | Shahvarpour et al. [32] | - | - | - | - |
|  | van den Hoorn et al. [35] | 0.2^e-2^ (0.5^e-2^) | 0.639 | −0.1^e-2^ (0.2^e-2^) | 0.560 |
| EO-ML | Larivière et al. [34] | - | - | - | - |
|  | Shahvarpour et al. [29] | - | - | - | - |
|  | Shahvarpour et al. [32] | - | - | - | - |
|  | van den Hoorn et al. [35] | 0.4^e-3^ (0.4^e-2^) | 0.916 | −0.7^e-3^ (0.2^e-2^) | 0.686 |
| EC-AP | Larivière et al. [34] | −0.01 (0.8^e-2^) | 0.08 | 0.2^e-2^ (0.5^e-2^) | 0.742 |
|  | Shahvarpour et al. [29] | −0.6^e-3^ (0.01) | 0.955 | −0.2^e-2^ (0.4^e-2^) | 0.618 |
|  | Shahvarpour et al. [32] | −0.01 (0.8^e-2^) | 0.134 | 0.4^e-2^ (0.4^e-2^) | 0.263 |
|  | van den Hoorn et al. [35] | −0.4^e-2^ (0.5^e-2^) | 0.365 | −0.4^e-3^ (0.2^e-2^) | 0.842 |
| EC-ML | Larivière et al. [34] | −0.8^e-2^ (0.9^e-2^) | 0.365 | 0.2^e-2^ (0.6^e-2^) | 0.751 |
|  | Shahvarpour et al. [29] | −0.2^e-3^ (0.01) | 0.985 | −0.4^e-4^ (0.4^e-2^) | 0.992 |
|  | Shahvarpour et al. [32] | −0.02 (0.9^e-2^) | 0.041 | 0.4^e-2^ (0.5^e-2^) | 0.355 |
|  | van den Hoorn et al. [35] | −0.4^e-2^ (0.4^e-2^) | 0.286 | −0.4^e-3^ (0.2^e-2^) | 0.797 |
| **Abbreviations:** IPD, individual participant data; LBP, low back pain; MPF, mean power frequency; VAS, visual analogue scale; NPRS, numeric pain rating scale; RMDQ, Roland-Morris disability questionnaire; Coef., coefficient; SE, standard error; EO, eyes open; EC, eyes closed; AP, anteroposterior; ML, mediolateral.  *P*-values of statistically significant regression coefficients (*P*<0.05) are printed bold. | | | | | |
