# Supplementary material for: Trunk postural control during unstable sitting among individuals with and without low back pain: A systematic review with an individual participant data meta-analysis
Source: PLoS One. 2024 Jan 24;19(1):e0296968. doi: 10.1371/journal.pone.0296968 (PMC10807788; doi:10.1371/journal.pone.0296968)
Supplement: S32 Table — (DOCX) [file pone.0296968.s033.docx]

| **Table S32.** Individual IPD analysis of associations between pain catastrophizing or fear-avoidance beliefs and MPF for each study | | | | | | | | | |
| --- | --- | --- | --- | --- | --- | --- | --- | --- | --- |
| **Outcome** | **Study** | **PCS** | | **FABQ-PA** | | **FABQ-W** | | **FABQ** | |
|  |  | **Coef. (SE)** | ***P*-value** | **Coef. (SE)** | ***P*-value** | **Coef. (SE)** | ***P*-value** | **Coef. (SE)** | ***P*-value** |
| EO-AP | Larivière et al. [34] | **-** | **-** | **-** | **-** | **-** | **-** | **-** | **-** |
|  | Shahvarpour et al. [29] | **-** | **-** | **-** | **-** | **-** | **-** | **-** | **-** |
|  | Shahvarpour et al. [32] | **-** | **-** | **-** | **-** | **-** | **-** | **-** | **-** |
|  | van den Hoorn et al. [35] | −0.1^e-2^ (0.9^e-3^) | 0.192 | 0.2^e-2^ (0.2^e-2^) | 0.314 | −0.2^e-3^ (0.9^e-3^) | 0.833 | 0.2^e-3^ (0.7^e-3^) | 0.752 |
| EO-ML | Larivière et al. [34] | **-** | **-** | **-** | **-** | **-** | **-** | **-** | **-** |
|  | Shahvarpour et al. [29] | **-** | **-** | **-** | **-** | **-** | **-** | **-** | **-** |
|  | Shahvarpour et al. [32] | **-** | **-** | **-** | **-** | **-** | **-** | **-** | **-** |
|  | van den Hoorn et al. [35] | −0.9^e-3^ (0.7^e-3^) | 0.256 | 0.3^e-3^ (0.1^e-2^) | 0.838 | 0.2^e-3^ (0.8^e-3^) | 0.845 | −0.3^e-4^ (0.7^e-3^) | 0.969 |
| EC-AP | Larivière et al. [34] | 0.1^e-2^ (0.2^e-2^) | 0.371 | **-** | **-** | **-** | **-** | **-** | **-** |
|  | Shahvarpour et al. [29] | −0.1^e-2^ (0.1^e-2^) | 0.299 | −0.4^e-2^ (0.3^e-2^) | 0.095 | **-** | **-** | **-** | **-** |
|  | Shahvarpour et al. [32] | −0.2^e-2^ (0.1^e-2^) | 0.064 | −0.5^e-3^ (0.2^e-2^) | 0.853 | −0.2^e-2^ (0.1^e-2^) | **0.032** | −0.2^e-2^ (0.8^e-3^) | 0.070 |
|  | van den Hoorn et al. [35] | −0.1^e-2^ (0.8^e-3^) | 0.126 | 0.5^e-3^ (0.2^e-2^) | 0.746 | −0.2^e-2^ (0.9^e-3^) | **0.013** | −0.1^e-2^ (0.7^e-3^) | **0.053** |
| EC-ML | Larivière et al. [34] | 0.1^e-2^ (0.2^e-2^) | 0.514 | **-** | **-** | **-** | **-** | **-** | **-** |
|  | Shahvarpour et al. [29] | −0.7^e-3^ (0.1^e-2^) | 0.628 | −0.4^e-2^ (0.3^e-2^) | 0.173 | **-** | **-** | **-** | **-** |
|  | Shahvarpour et al. [32] | −0.2^e-2^ (0.2^e-2^) | 0.260 | 0.2^e-2^ (0.3^e-2^) | 0.572 | −0.2^e-2^ (0.1^e-2^) | 0.134 | −0.1^e-2^ (0.1^e-2^) | 0.311 |
|  | van den Hoorn et al. [35] | −0.6^e-3^ (0.8^e-3^) | 0.411 | −0.4^e-3^ (0.1^e-2^) | 0.782 | −0.8^e-3^ (0.8^e-3^) | 0.334 | −0.6^e-3^ (0.6^e-3^) | 0.385 |
| **Abbreviations:** IPD, individual participant data; MPF, mean power frequency; PCS, pain catastrophizing scale; FABQ-PA, fear-avoidance beliefs questionnaire - physical activity; FABQ-W, fear-avoidance beliefs questionnaire - work; FABQ, fear-avoidance beliefs questionnaire; Coef., coefficient; SE, standard error; EO, eyes open; EC, eyes closed; AP, anteroposterior; ML, mediolateral.  *P*-values of statistically significant regression coefficients (*P*<0.05) are printed bold. | | | | | | | | | |
