# Supplementary material for: Trunk postural control during unstable sitting among individuals with and without low back pain: A systematic review with an individual participant data meta-analysis
Source: PLoS One. 2024 Jan 24;19(1):e0296968. doi: 10.1371/journal.pone.0296968 (PMC10807788; doi:10.1371/journal.pone.0296968)
Supplement: S33 Table — (DOCX) [file pone.0296968.s034.docx]

| **Table S33.** Individual IPD analysis of associations between LBP intensity or disability and stabilogram diffusion measures* for each study | | | | | | |
| --- | --- | --- | --- | --- | --- | --- |
| **Outcome** | | **Study** | **VAS/NPRS** | | **RMDQ** | |
|  |  |  | **Coef. (SE)** | ***P*-value** | **Coef. (SE)** | ***P*-value** |
| D_short_ | EO-AP | van den Hoorn et al. [35] | 0.58 (0.51) | 0.259 | 0.04 (0.20) | 0.841 |
|  | EO-ML | van den Hoorn et al. [35] | 0.19 (0.41) | 0.645 | 0.12 (0.16) | 0.456 |
|  | EC-AP | van den Hoorn et al. [35] | 0.97 (3.19) | 0.762 | −1.69 (1.25) | 0.177 |
|  | EC-ML | van den Hoorn et al. [35] | −0.05 (2.22) | 0.982 | −0.82 (0.87) | 0.346 |
| D_long_ | EO-AP | van den Hoorn et al. [35] | 0.09 (0.08) | 0.259 | 0.03 (0.03) | 0.327 |
|  | EO-ML | van den Hoorn et al. [35] | −0.02 (0.06) | 0.682 | −0.02 (0.02) | 0.264 |
|  | EC-AP | van den Hoorn et al. [35] | −0.07 (0.74) | 0.927 | −0.01 (0.29) | 0.986 |
|  | EC-ML | van den Hoorn et al. [35] | 0.41 (0.43) | 0.341 | −0.13 (0.17) | 0.439 |
| CP_dist_ | EO-AP | van den Hoorn et al. [35] | 2.25 (2.20) | 0.307 | 0.36 (0.87) | 0.681 |
|  | EO-ML | van den Hoorn et al. [35] | 1.64 (2.32) | 0.479 | 1.31 (0.91) | 0.150 |
|  | EC-AP | van den Hoorn et al. [35] | 12.04 (10.37) | 0.246 | −3.27 (4.10) | 0.426 |
|  | EC-ML | van den Hoorn et al. [35] | −2.93 (9.65) | 0.761 | −1.14 (3.81) | 0.764 |
| CP_time_ | EO-AP | van den Hoorn et al. [35] | −0.01 (0.03) | 0.629 | 0.3^e-2^ (0.01) | 0.800 |
|  | EO-ML | van den Hoorn et al. [35] | 0.01 (0.03) | 0.730 | 0.02 (0.01) | 0.109 |
|  | EC-AP | van den Hoorn et al. [35] | 0.05 (0.04) | 0.205 | 0.01 (0.02) | 0.528 |
|  | EC-ML | van den Hoorn et al. [35] | −0.8^e-3^ (0.03) | 0.980 | 0.01 (0.01) | 0.389 |
| **Abbreviations:** IPD, individual participant data; LBP, low back pain; D_short_, short-term diffusion coefficient; D_long_, long-term diffusion coefficient; CP_dist_, mean squared distance coordinate of the critical point; CP_time_, mean time coordinate of the critical point; VAS, visual analogue scale; NPRS, numeric pain rating scale; RMDQ, Roland-Morris disability questionnaire; Coef., coefficient; SE, standard error; EO, eyes open; EC, eyes closed; AP, anteroposterior; ML, mediolateral.  *P*-values of statistically significant regression coefficients (*P*<0.05) are printed bold.  *IPD analysis for stabilogram diffusion outcome measures was possible for one study. | | | | | | |
