# Supplementary material for: Trunk postural control during unstable sitting among individuals with and without low back pain: A systematic review with an individual participant data meta-analysis
Source: PLoS One. 2024 Jan 24;19(1):e0296968. doi: 10.1371/journal.pone.0296968 (PMC10807788; doi:10.1371/journal.pone.0296968)
Supplement: S34 Table — (DOCX) [file pone.0296968.s035.docx]

| **Table S34.** Individual IPD analysis of associations between pain catastrophizing or fear-avoidance beliefs and stabilogram diffusion measures* for each study | | | | | | | | | | |
| --- | --- | --- | --- | --- | --- | --- | --- | --- | --- | --- |
| **Outcome** | | | **PCS** | | **FABQ-PA** | | **FABQ-W** | | **FABQ** | |
|  |  |  | **Coef. (SE)** | ***P*-value** | **Coef. (SE)** | ***P*-value** | **Coef. (SE)** | ***P*-value** | **Coef. (SE)** | ***P*-value** |
| D_short_ | EO-AP | van den Hoorn et al. [35] | 0.06 (0.10) | 0.521 | −0.20 (0.18) | 0.262 | 0.11 (0.10) | 0.272 | 0.03 (0.08) | 0.704 |
|  | EO-ML | van den Hoorn et al. [35] | 0.07 (0.08) | 0.325 | 0.05 (0.14) | 0.715 | 0.10 (0.08) | 0.218 | 0.08 (0.07) | 0.223 |
|  | EC-AP | van den Hoorn et al. [35] | −0.12 (0.59) | 0.839 | −0.29 (1.08) | 0.788 | −0.46 (0.62) | 0.458 | −0.32 (0.51) | 0.529 |
|  | EC-ML | van den Hoorn et al. [35] | −0.12 (0.41) | 0.762 | 0.36 (0.76) | 0.634 | −0.32 (0.43) | 0.462 | −0.10 (0.36) | 0.772 |
| D_long_ | EO-AP | van den Hoorn et al. [35] | 0.03 (0.01) | **0.023** | 0.5^e-2^ (0.03) | 0.865 | −0.02 (0.02) | 0.277 | −0.01 (0.01) | 0.447 |
|  | EO-ML | van den Hoorn et al. [35] | −0.3^e-2^ (0.01) | 0.803 | −0.2^e-2^ (0.02) | 0.919 | −0.7^e-2^ (0.01) | 0.498 | −0.5^e-2^ (0.9^e-2^) | 0.581 |
|  | EC-AP | van den Hoorn et al. [35] | 0.19 (0.14) | 0.165 | −0.06 (0.25) | 0.802 | −0.09 (0.14) | 0.541 | −0.06 (0.12) | 0.597 |
|  | EC-ML | van den Hoorn et al. [35] | −0.9^e-3^ (0.08) | 0.992 | −0.12 (0.15) | 0.400 | −0.05 (0.08) | 0.537 | −0.07 (0.07) | 0.343 |
| CP_dist_ | EO-AP | van den Hoorn et al. [35] | 0.48 (0.41) | 0.237 | −1.11 (0.75) | 0.135 | 0.55 (0.43) | 0.201 | 0.13 (0.35) | 0.724 |
|  | EO-ML | van den Hoorn et al. [35] | 0.55 (0.43) | 0.197 | 0.25 (0.79) | 0.755 | 0.40 (0.45) | 0.376 | 0.33 (0.37) | 0.375 |
|  | EC-AP | van den Hoorn et al. [35] | 3.70 (1.91) | **0.052** | −0.74 (3.55) | 0.834 | 3.15 (2.01) | 0.116 | 2.14 (1.66) | 0.198 |
|  | EC-ML | van den Hoorn et al. [35] | 0.17 (1.79) | 0.923 | 2.70 (3.28) | 0.410 | −1.69 (1.87) | 0.367 | −0.48 (1.55) | 0.759 |
| CP_time_ | EO-AP | van den Hoorn et al. [35] | 0.3^e-2^ (0.6^e-2^) | 0.580 | −0.03 (0.01) | **0.012** | 0.3^e-2^ (0.6^e-2^) | 0.578 | −0.3^e-2^ (0.5^e-2^) | 0.544 |
|  | EO-ML | van den Hoorn et al. [35] | 0.7^e-2^ (0.6^e-2^) | 0.244 | 0.01 (0.01) | 0.212 | −0.2^e-2^ (0.6^e-2^) | 0.774 | 0.2^e-2^ (0.5^e-2^) | 0.664 |
|  | EC-AP | van den Hoorn et al. [35] | 0.02 (0.7^e-2^) | **0.024** | 0.6^e-2^ (0.01) | 0.666 | 0.01 (0.8^e-2^) | **0.057** | 0.01 (0.6^e-2^) | 0.067 |
|  | EC-ML | van den Hoorn et al. [35] | −0.1^e-2^ (0.6^e-2^) | 0.846 | 0.6^e-2^ (0.01) | 0.624 | −0.1^e-2^ (0.6^e-2^) | 0.878 | −0.5^e-4^ (0.5^e-2^) | 0.992 |
| **Abbreviations:** IPD, individual participant data; PCS, pain catastrophizing scale; FABQ-PA, fear-avoidance beliefs questionnaire - physical activity; FABQ-W, fear-avoidance beliefs questionnaire - work; FABQ, fear-avoidance beliefs questionnaire; D_short_, short-term diffusion coefficient; D_long_, long-term diffusion coefficient; CP_dist_, mean squared distance coordinate of the critical point; CP_time_, mean time coordinate of the critical point; Coef., coefficient; SE, standard error; EO, eyes open; EC, eyes closed; AP, anteroposterior; ML, mediolateral.  *P*-values of statistically significant regression coefficients (*P*<0.05) are printed bold.  *IPD analysis for stabilogram diffusion measures was possible for one study. | | | | | | | | | | |
