# Supplementary figures and images for: Trunk postural control during unstable sitting among individuals with and without low back pain: A systematic review with an individual participant data meta-analysis
Source: PLoS One. 2024 Jan 24;19(1):e0296968. doi: 10.1371/journal.pone.0296968 (PMC10807788; doi:10.1371/journal.pone.0296968)

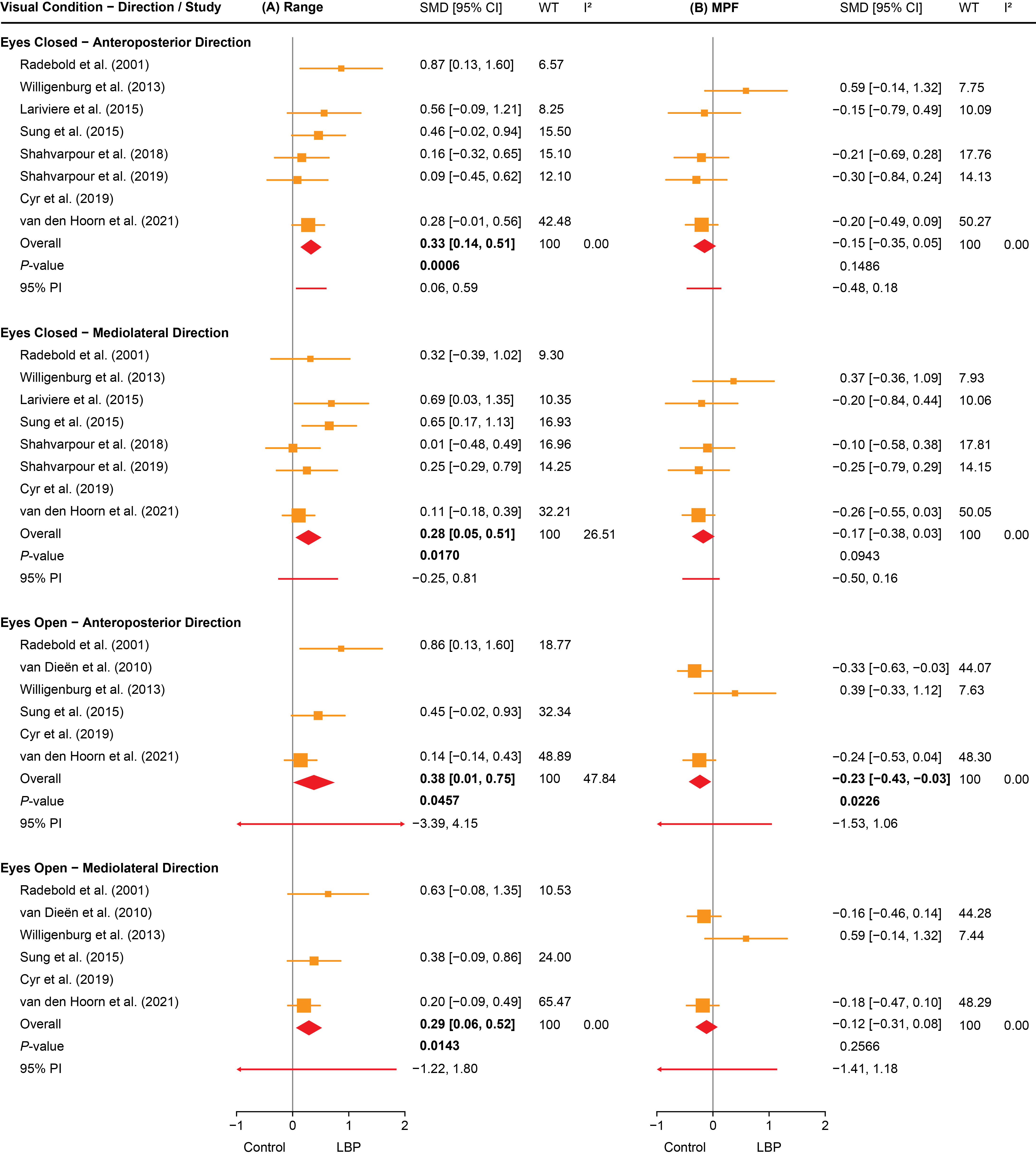

Supplement: S1 Fig — (A) range and (B) mean power frequency (MPF). The results are presented as standardised mean differences (SMDs) with 95% confidence intervals (95% CIs) using forest plots. Significant overall effect sizes with their respective P-values are highlighted in bold font. Sizing of squares reflects the weight (WT) of the contribution of a study on the pooled meta-analysis (weighted average) in percentage. I2 reflects the percentage of total variability due to heterogeneity between studies. 95% prediction interval (95% PI) reflects how much the effect size varies across studies. (JPG) [file pone.0296968.s036.jpg]

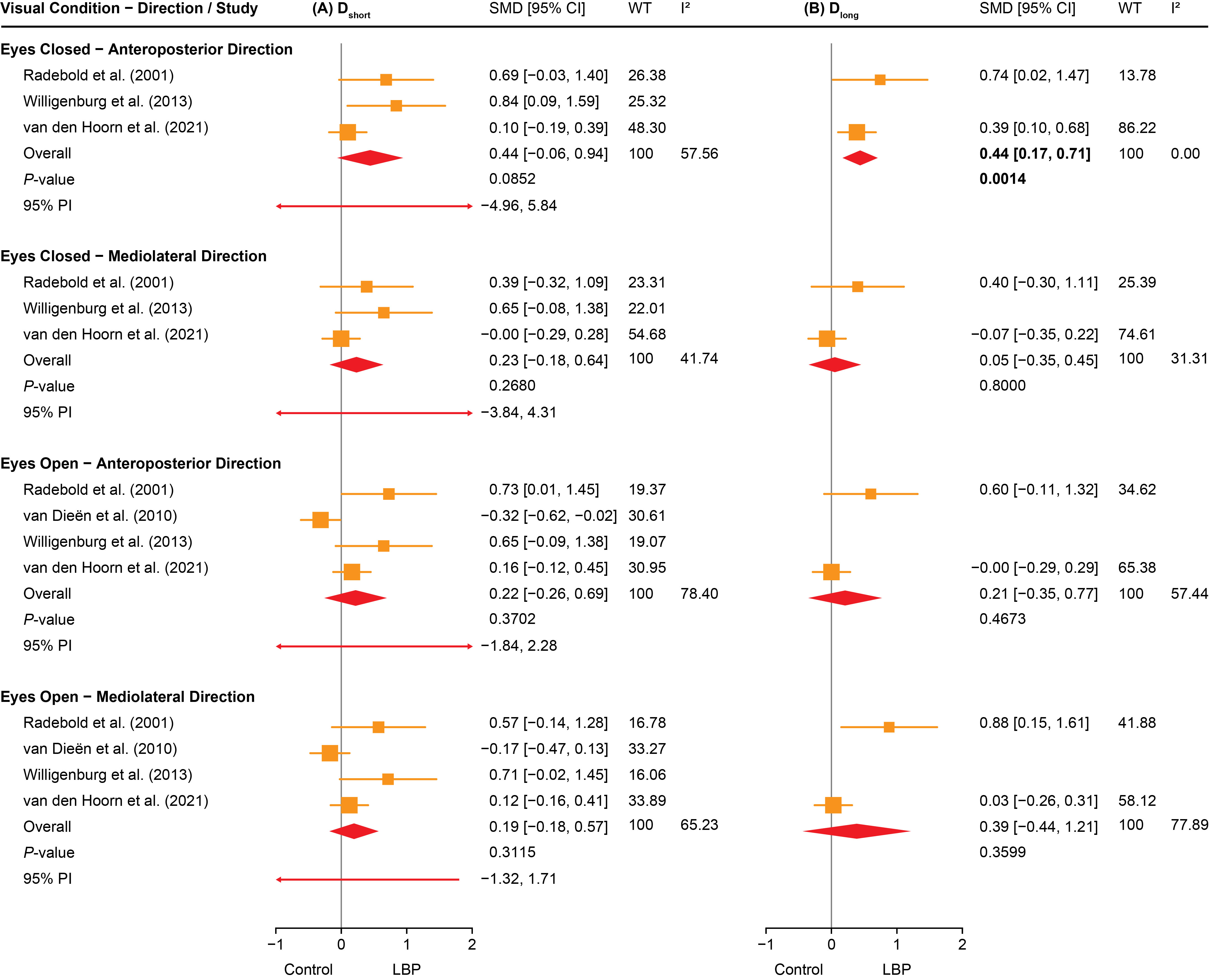

Supplement: S2 Fig — (A) short-term diffusion (Dshort) and (B) long-term diffusion (Dlong). The results are presented as standardised mean differences (SMDs) with 95% confidence intervals (95% CIs) using forest plots. Significant overall effect sizes with their respective P-values are highlighted in bold font. Sizing of squares reflects the weight (WT) of the contribution of a study on the pooled meta-analysis (weighted average) in percentage. I2 reflects the percentage of total variability due to heterogeneity between studies. 95% prediction interval (95% PI) reflects how much the effect size varies across studies. (JPG) [file pone.0296968.s037.jpg]

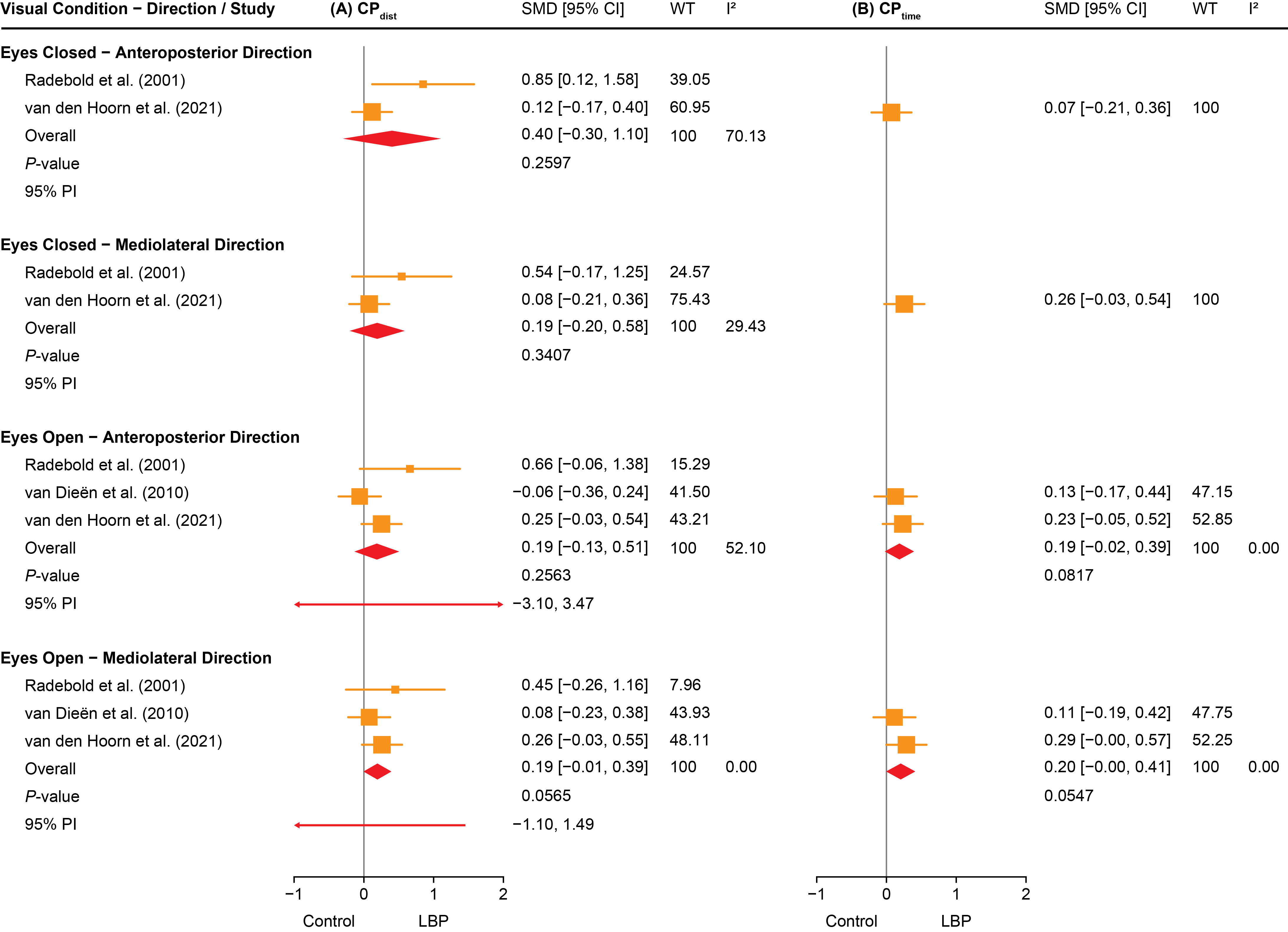

Supplement: S3 Fig — (A) mean squared distance coordinate of the critical point (CPdist) and (B) mean time coordinate of the critical point (CPtime). The results are presented as standardised mean differences (SMDs) with 95% confidence intervals (95% CIs) using forest plots. Significant overall effect sizes with their respective P-values are highlighted in bold font. Sizing of squares reflects the weight (WT) of the contribution of a study on the pooled meta-analysis (weighted average) in percentage. I2 reflects the percentage of total variability due to heterogeneity between studies. 95% prediction interval (95% PI) reflects how much the effect size varies across studies. (JPG) [file pone.0296968.s038.jpg]

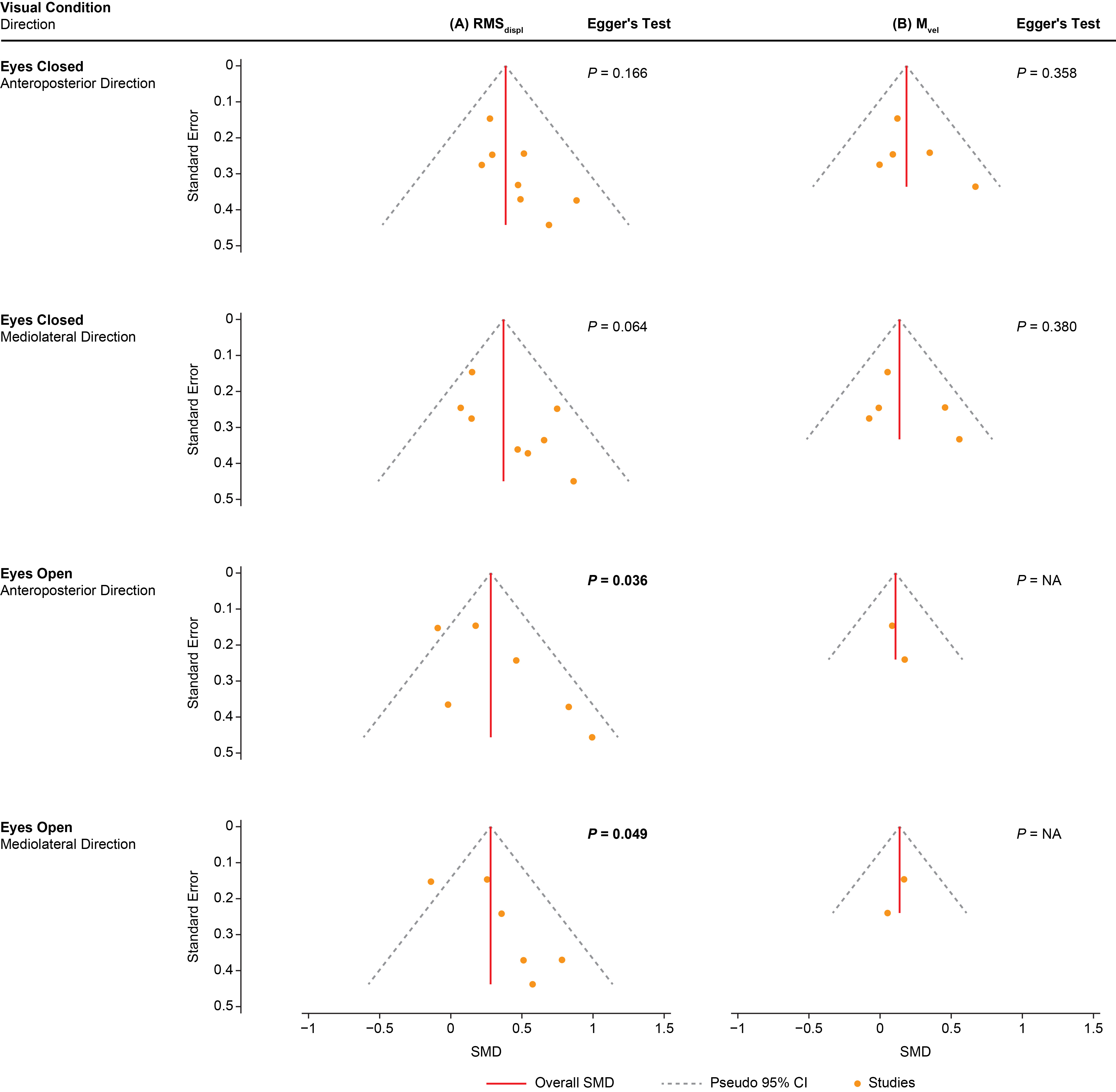

Supplement: S4 Fig — (A) root mean square displacement (RMSdispl) and (B) mean velocity (Mvel). The vertical solid red lines represent the overall standardized mean differences (SMDs) from the IPD meta-analysis. The two diagonal dashed lines in both sides represent the pseudo 95% confidence intervals (95% CIs) around the overall SMDs for each standard error (precision). Each orange dot represents a SMD for an individual study. Small studies are scattered more widely at the bottom of the funnel plot (lower precision) and larger studies are scattered more at the top of the funnel plot (greater precision). P-values for the potential presence of significant small-study effects (funnel plot asymmetry) are highlighted in bold font. (JPG) [file pone.0296968.s039.jpg]

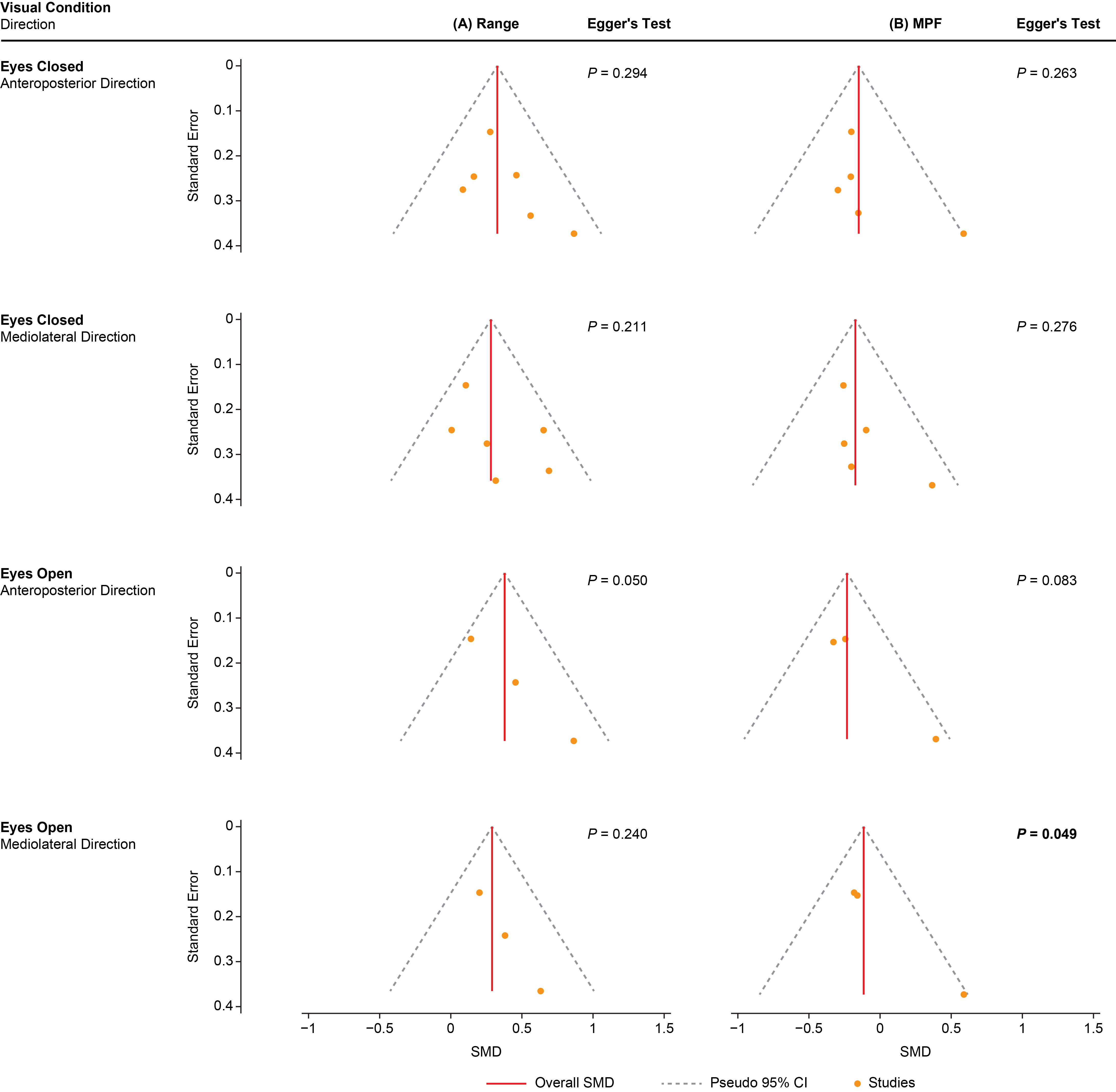

Supplement: S5 Fig — (A) range and (B) mean power frequency (MPF). The vertical solid red lines represent the overall standardized mean differences (SMDs) from the IPD meta-analysis. The two diagonal dashed lines in both sides represent the pseudo 95% confidence intervals (95% CIs) around the overall SMDs for each standard error (precision). Each orange dot represents a SMD for an individual study. Small studies are scattered more widely at the bottom of the funnel plot (lower precision) and larger studies are scattered more at the top of the funnel plot (greater precision). P-values for the potential presence of significant small-study effects (funnel plot asymmetry) are highlighted in bold font. (JPG) [file pone.0296968.s040.jpg]

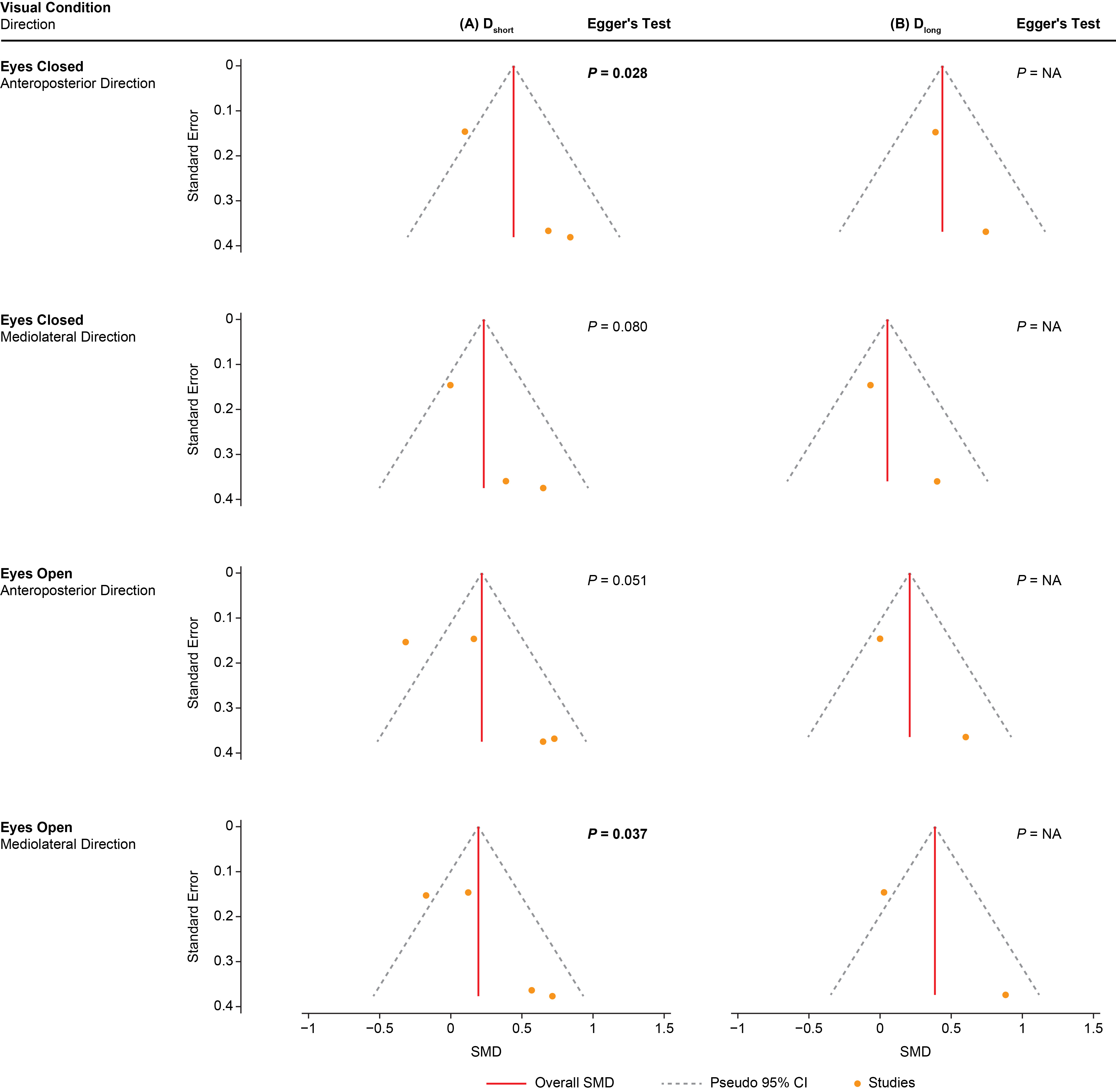

Supplement: S6 Fig — (A) short-term diffusion (Dshort) and (B) long-term diffusion (Dlong). The vertical solid red lines represent the overall standardized mean differences (SMDs) from the IPD meta-analysis. The two diagonal dashed lines in both sides represent the pseudo 95% confidence intervals (95% CIs) around the overall SMDs for each standard error (precision). Each orange dot represents a SMD for an individual study. Small studies are scattered more widely at the bottom of the funnel plot (lower precision) and larger studies are scattered more at the top of the funnel plot (greater precision). P-values for the potential presence of significant small-study effects (funnel plot asymmetry) are highlighted in bold font. (JPG) [file pone.0296968.s041.jpg]

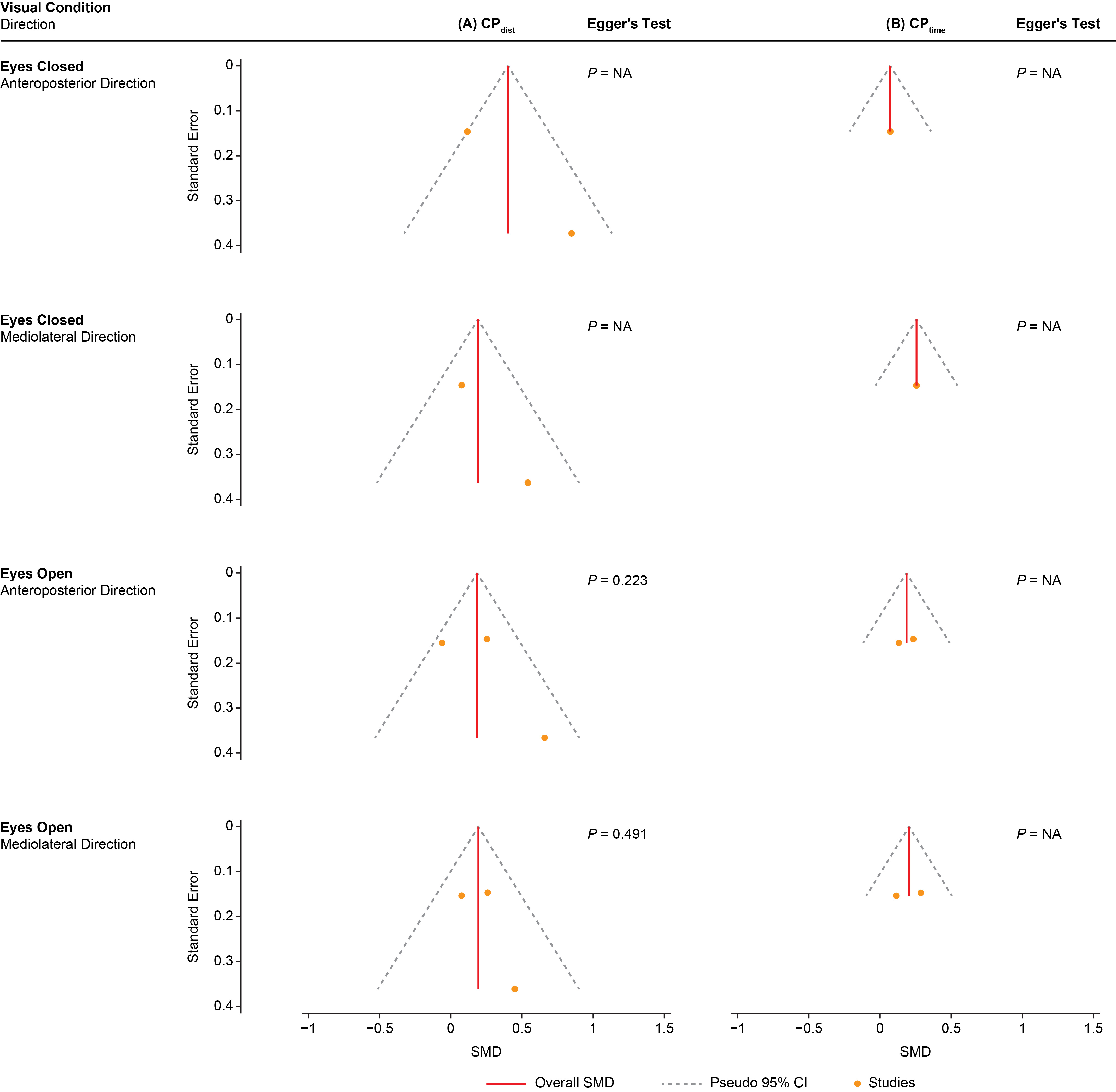

Supplement: S7 Fig — (A) mean squared distance coordinate of the critical point (CPdist) and (B) mean time coordinate of the critical point (CPtime). The vertical solid red lines represent the overall standardized mean differences (SMDs) from the IPD meta-analysis. The two diagonal dashed lines in both sides represent the pseudo 95% confidence intervals (95% CIs) around the overall SMDs for each standard error (precision). Each orange dot represents a SMD for an individual study. Small studies are scattered more widely at the bottom of the funnel plot (lower precision) and larger studies are scattered more at the top of the funnel plot (greater precision). P-values for the potential presence of significant small-study effects (funnel plot asymmetry) are highlighted in bold font. (JPG) [file pone.0296968.s042.jpg]

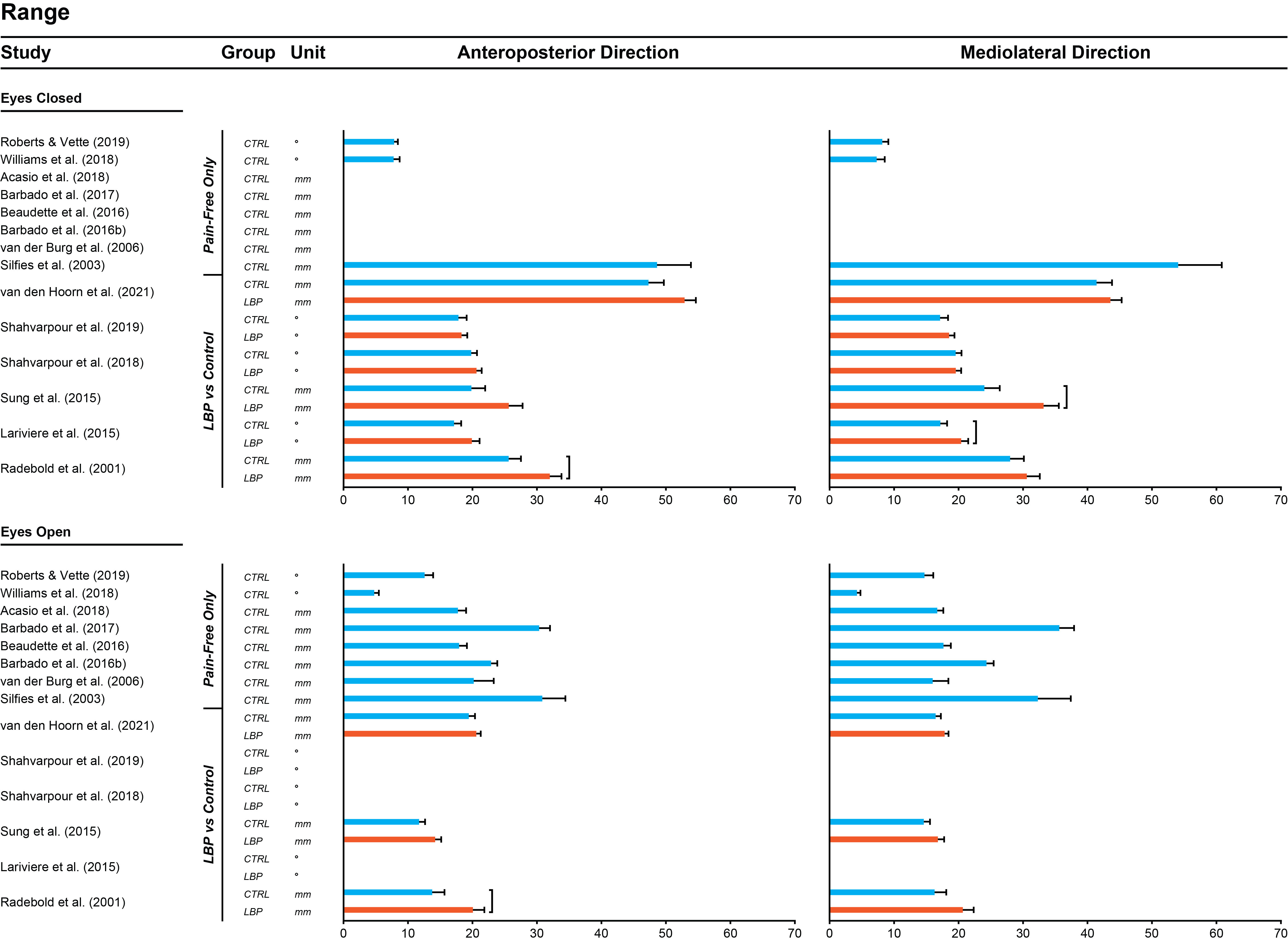

Supplement: S8 Fig — Mean plots of the results from studies that were included in the individual participant data (IPD) meta-analysis (individuals with versus without low back pain [LBP]) are presented with mean plots of the results from studies that tested only pain-free individuals [CTRL]. The results are presented as means with standard errors. For studies with two groups, significant differences between individuals with and without LBP are shown with square bracket. No bars in some studies = no data available. (JPG) [file pone.0296968.s043.jpg]

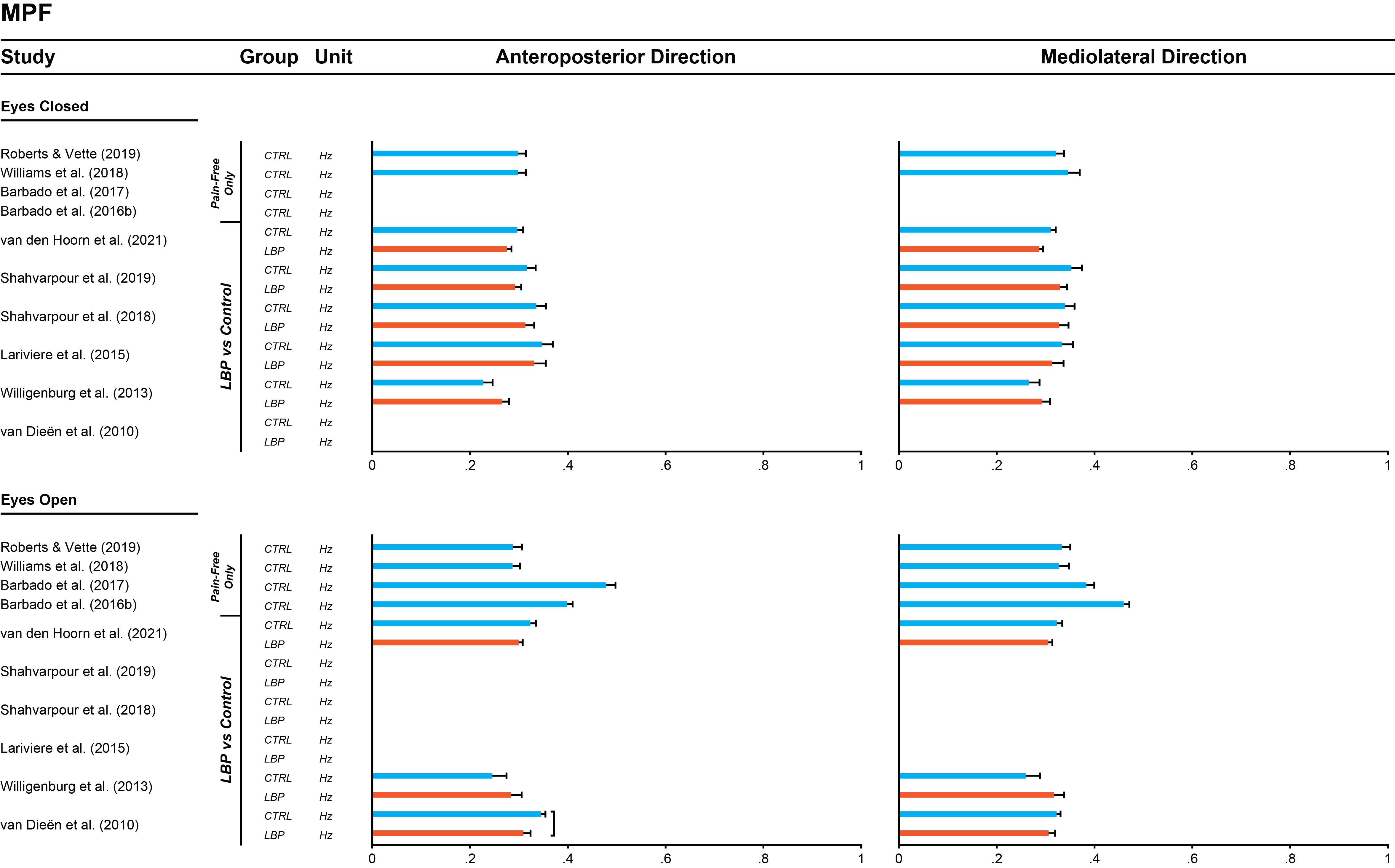

Supplement: S9 Fig — Mean plots of the results from studies that were included in the individual participant data (IPD) meta-analysis (individuals with versus without low back pain [LBP]) are presented with mean plots of the results from studies that tested only pain-free individuals [CTRL]. The results are presented as means with standard errors. For studies with two groups, significant differences between individuals with and without LBP are shown with square bracket. No bars in some studies = no data available. (JPG) [file pone.0296968.s044.jpg]

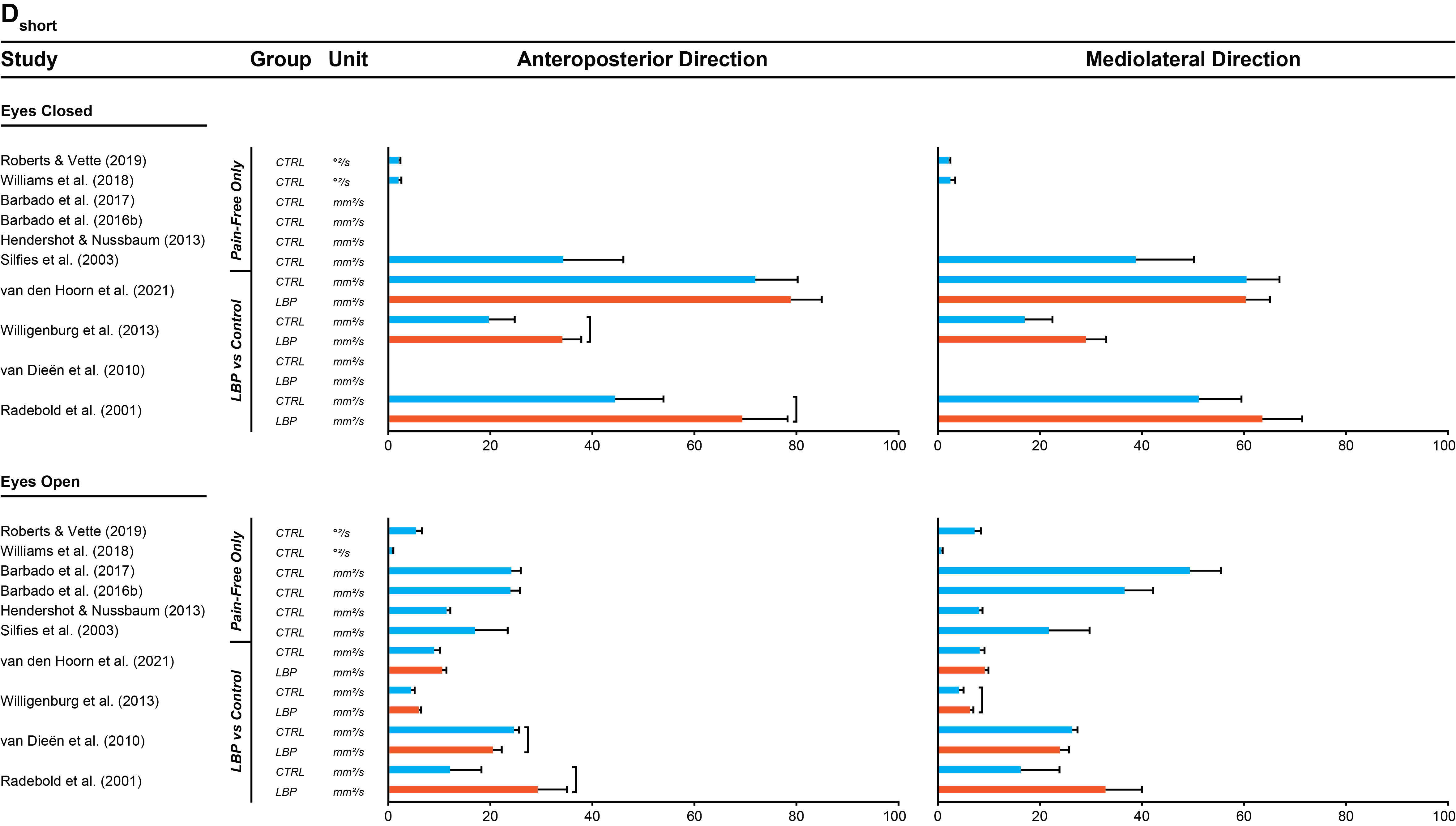

Supplement: S10 Fig — Mean plots of the results from studies that were included in the individual participant data (IPD) meta-analysis (individuals with versus without low back pain [LBP]) are presented with mean plots of the results from studies that tested only pain-free individuals [CTRL]. The results are presented as means with standard errors. For studies with two groups, significant differences between individuals with and without LBP are shown with square bracket. No bars in some studies = no data available. (JPG) [file pone.0296968.s045.jpg]

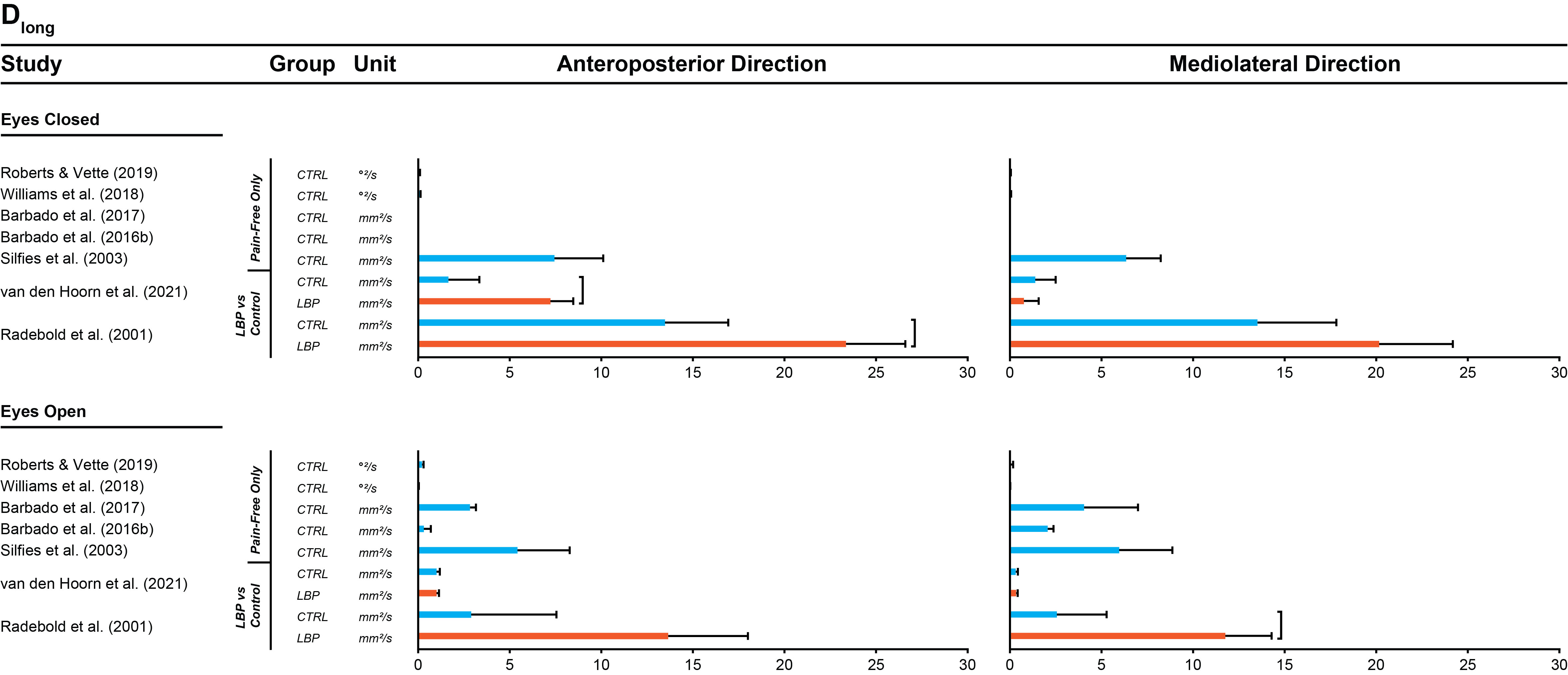

Supplement: S11 Fig — Mean plots of the results from studies that were included in the individual participant data (IPD) meta-analysis (individuals with versus without low back pain [LBP]) are presented with mean plots of the results from studies that tested only pain-free individuals [CTRL]. The results are presented as means with standard errors. For studies with two groups, significant differences between individuals with and without LBP are shown with square bracket. No bars in some studies = no data available. (JPG) [file pone.0296968.s046.jpg]

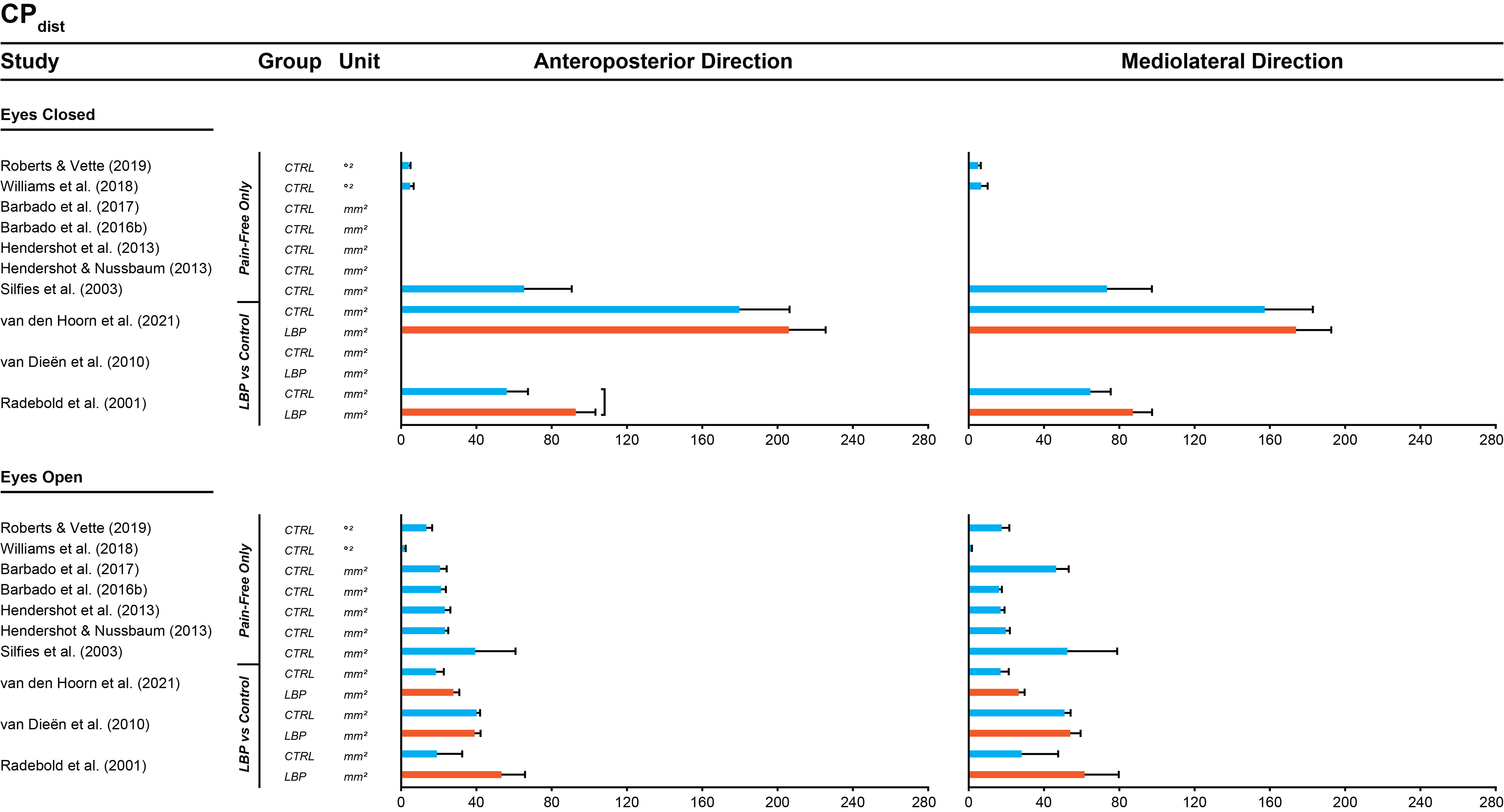

Supplement: S12 Fig — Mean plots of the results from studies that were included in the individual participant data (IPD) meta-analysis (individuals with versus without low back pain [LBP]) are presented with mean plots of the results from studies that tested only pain-free individuals [CTRL]. The results are presented as means with standard errors. For studies with two groups, significant differences between individuals with and without LBP are shown with square bracket. No bars in some studies = no data available. (JPG) [file pone.0296968.s047.jpg]

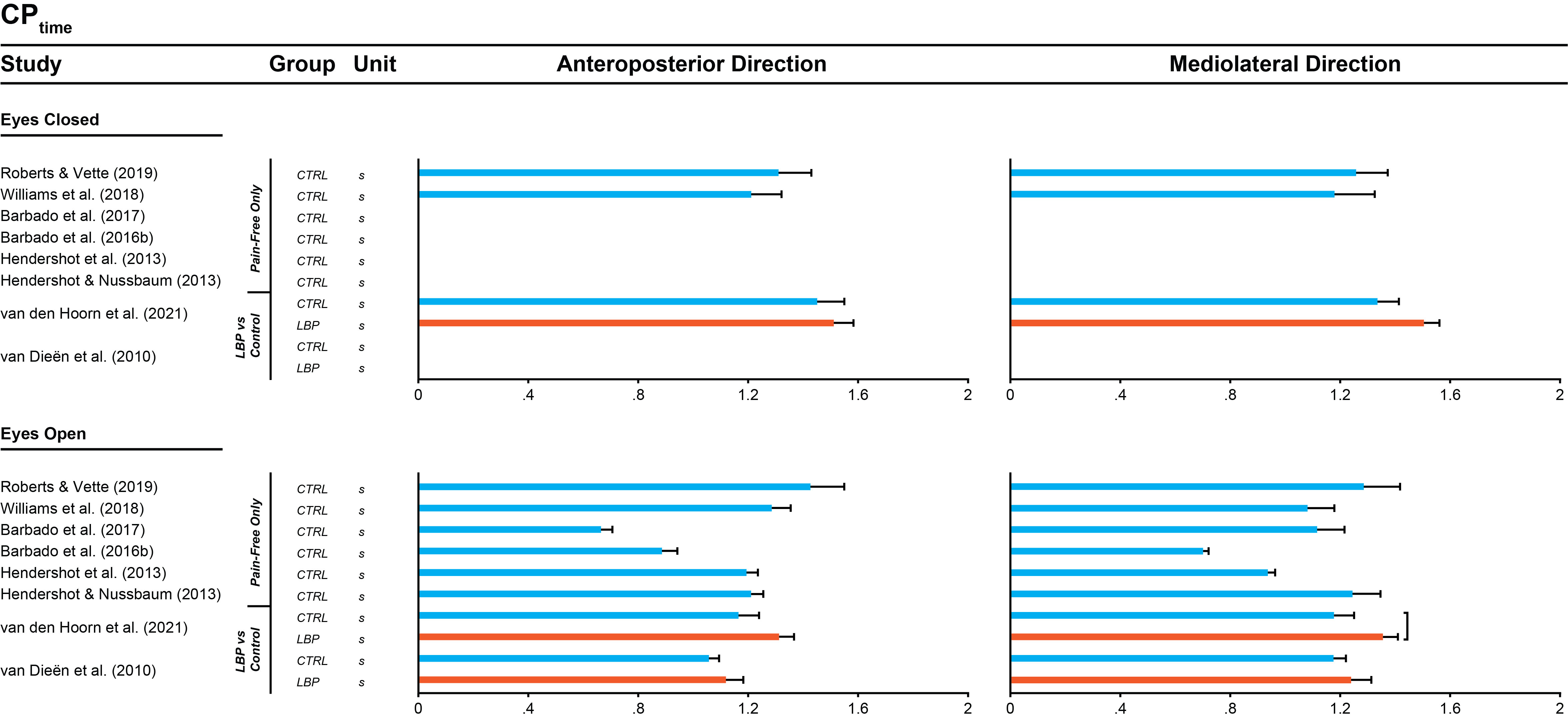

Supplement: S13 Fig — Mean plots of the results from studies that were included in the individual participant data (IPD) meta-analysis (individuals with versus without low back pain [LBP]) are presented with mean plots of the results from studies that tested only pain-free individuals [CTRL]. The results are presented as means with standard errors. For studies with two groups, significant differences between individuals with and without LBP are shown with square bracket. No bars in some studies = no data available. (JPG) [file pone.0296968.s048.jpg]

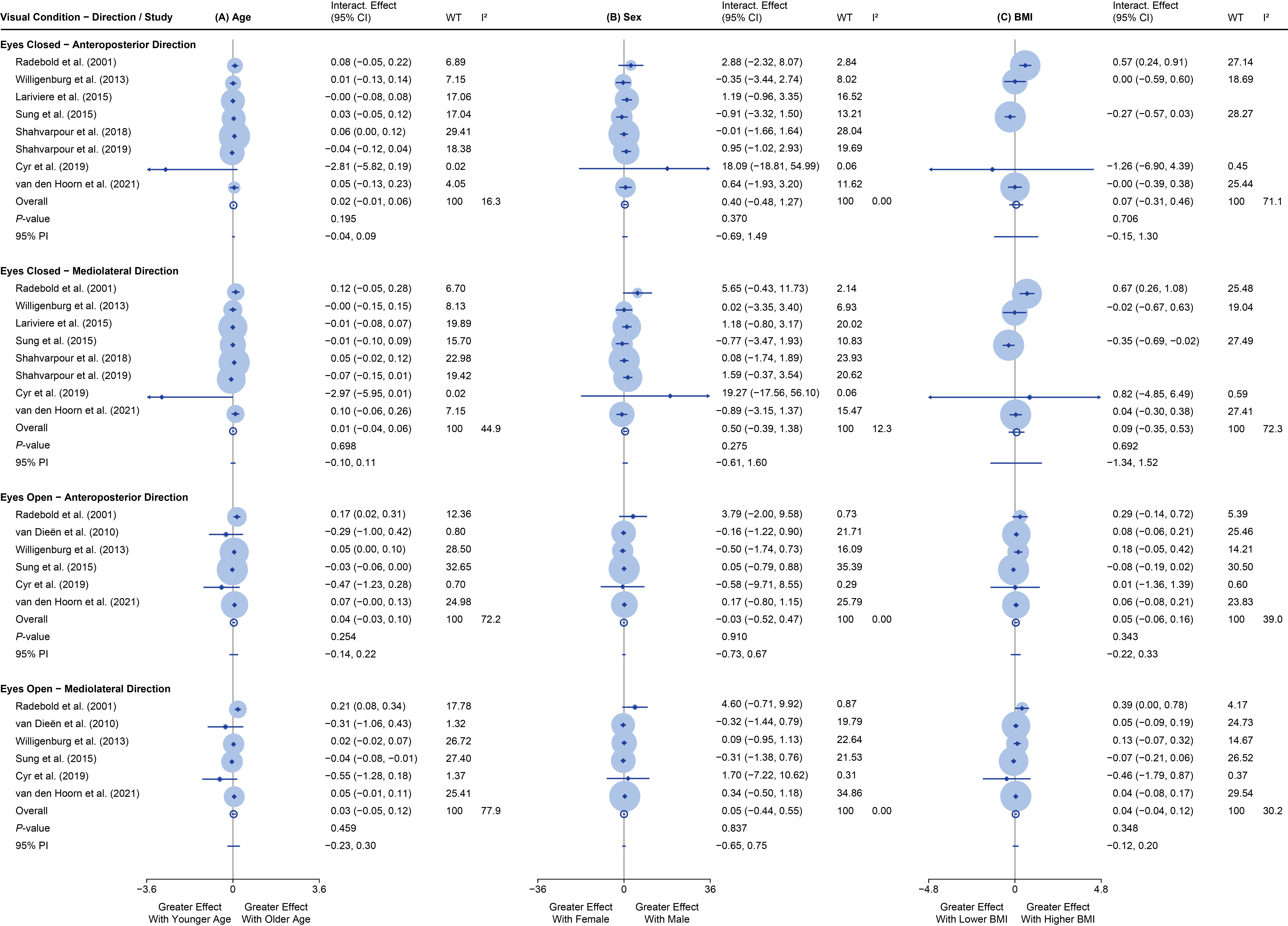

Supplement: S14 Fig — (A) age, (B) sex, and (C) body mass index (BMI). The results are presented as interaction (interact.) effect coefficients with 95% confidence intervals (95% CIs) using forest plots. Significant overall interact. effects with their respective P-values are highlighted in bold font. Greater effect in either directions indicates worse effect on trunk postural control for individuals with versus without low back pain. Sizing of circles reflects the weight (WT) of the contribution of a study on the pooled meta-analysis (weighted average) in percentage. I2 reflects the percentage of total variability due to heterogeneity between studies. 95% prediction interval (95% PI) reflects how much the effect size varies across studies. (JPG) [file pone.0296968.s049.jpg]

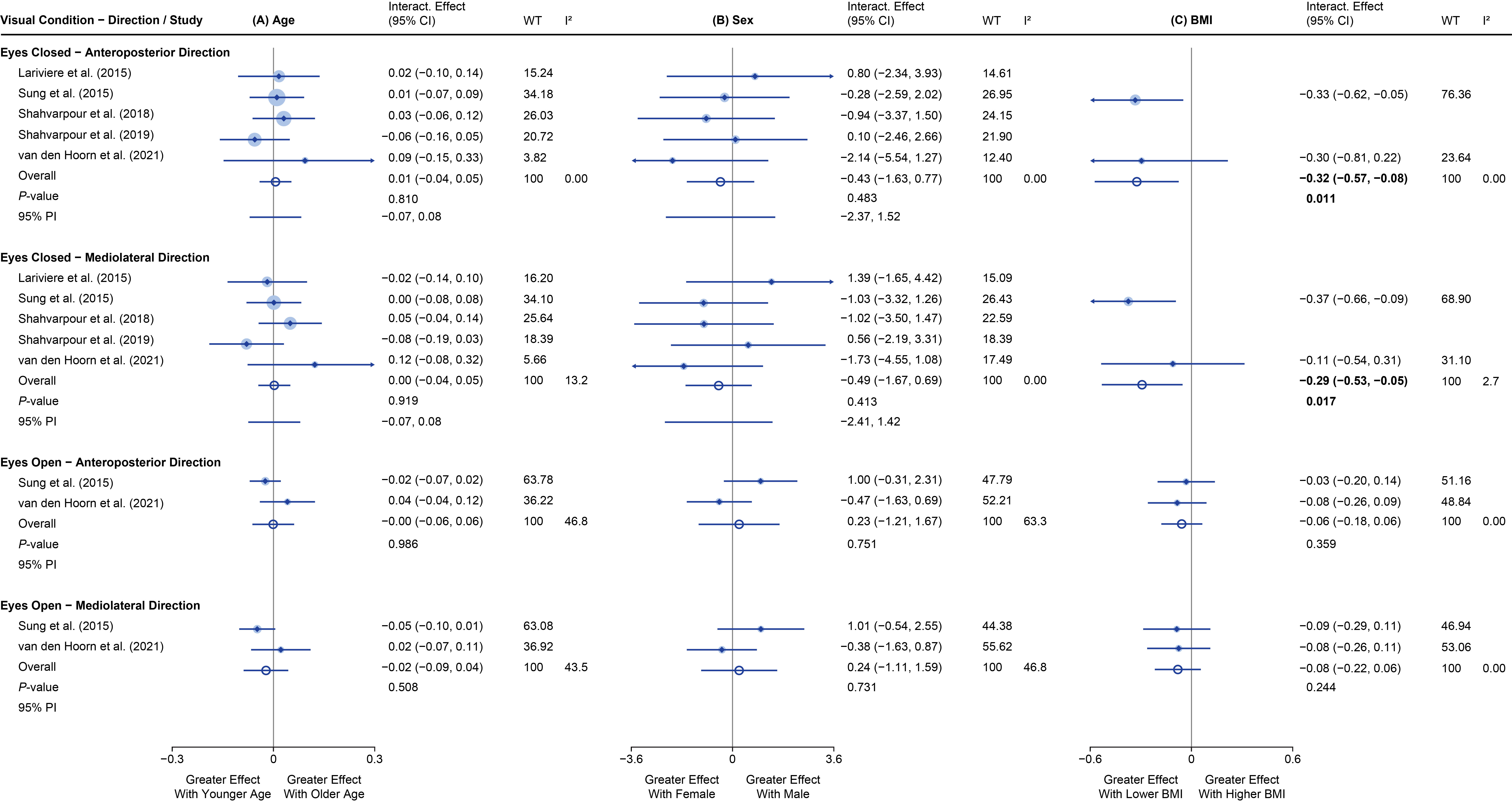

Supplement: S15 Fig — (A) age, (B) sex, and (C) body mass index (BMI). The results are presented as interaction (interact.) effect coefficients with 95% confidence intervals (95% CIs) using forest plots. Significant overall interact. effects with their respective P-values are highlighted in bold font. Greater effect in either directions indicates worse effect on trunk postural control for individuals with versus without low back pain. Sizing of circles reflects the weight (WT) of the contribution of a study on the pooled meta-analysis (weighted average) in percentage. I2 reflects the percentage of total variability due to heterogeneity between studies. 95% prediction interval (95% PI) reflects how much the effect size varies across studies. (JPG) [file pone.0296968.s050.jpg]

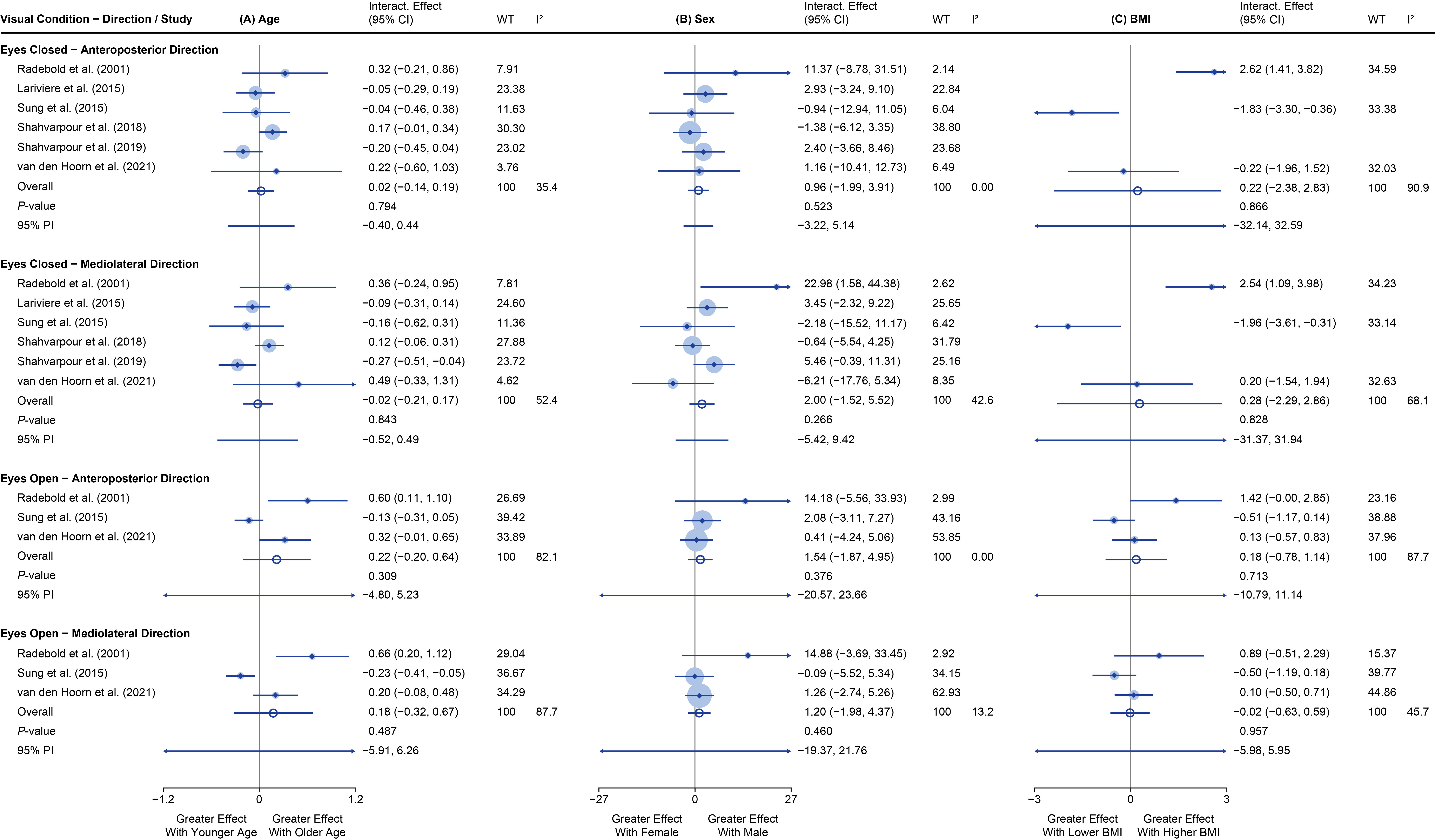

Supplement: S16 Fig — (A) age, (B) sex, and (C) body mass index (BMI). The results are presented as interaction (interact.) effect coefficients with 95% confidence intervals (95% CIs) using forest plots. Significant overall interact. effects with their respective P-values are highlighted in bold font. Greater effect in either directions indicates worse effect on trunk postural control for individuals with versus without low back pain. Sizing of circles reflects the weight (WT) of the contribution of a study on the pooled meta-analysis (weighted average) in percentage. I2 reflects the percentage of total variability due to heterogeneity between studies. 95% prediction interval (95% PI) reflects how much the effect size varies across studies. (JPG) [file pone.0296968.s051.jpg]

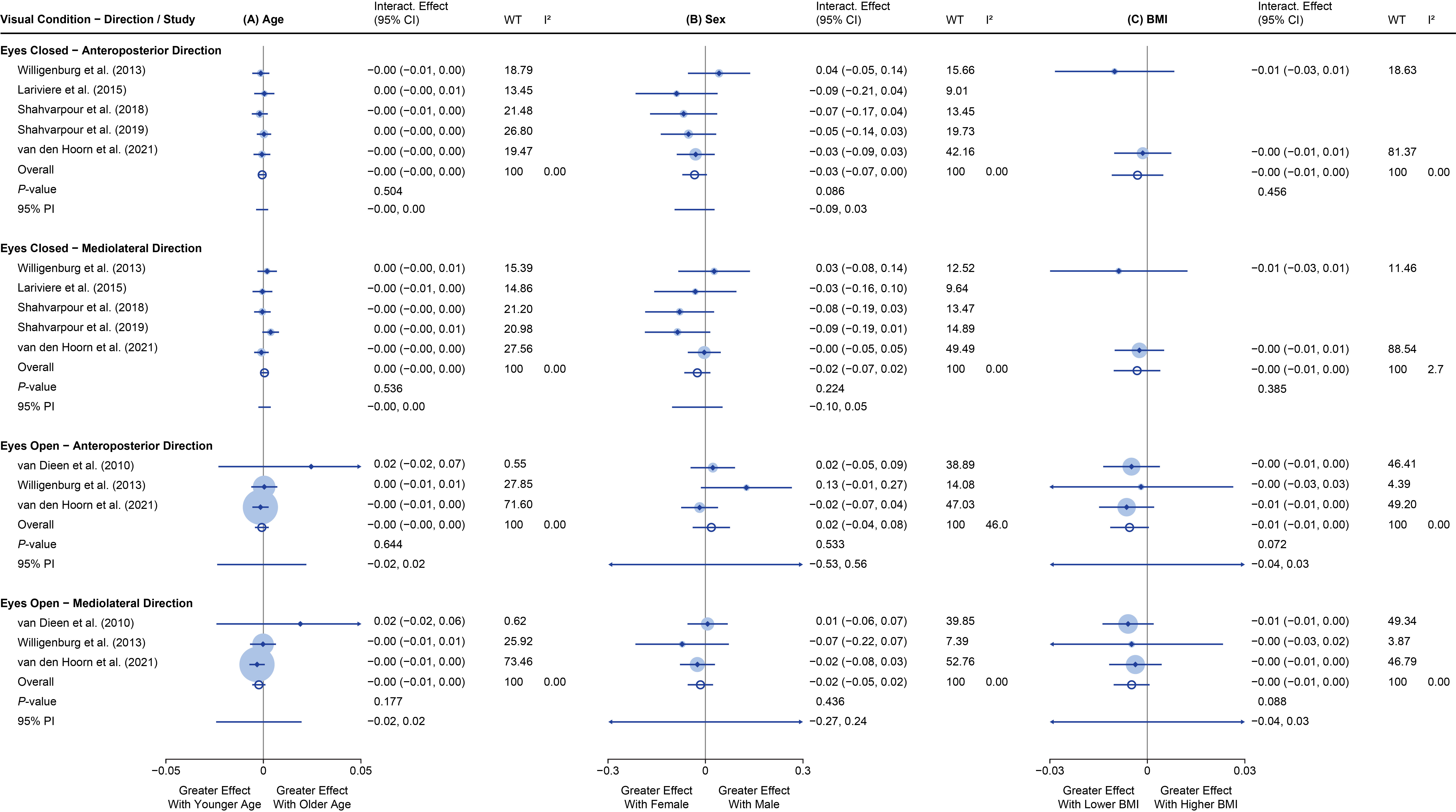

Supplement: S17 Fig — (A) age, (B) sex, and (C) body mass index (BMI). The results are presented as interaction (interact.) effect coefficients with 95% confidence intervals (95% CIs) using forest plots. Significant overall interact. effects with their respective P-values are highlighted in bold font. Greater effect in either directions indicates worse effect on trunk postural control for individuals with versus without low back pain. Sizing of circles reflects the weight (WT) of the contribution of a study on the pooled meta-analysis (weighted average) in percentage. I2 reflects the percentage of total variability due to heterogeneity between studies. 95% prediction interval (95% PI) reflects how much the effect size varies across studies. (JPG) [file pone.0296968.s052.jpg]

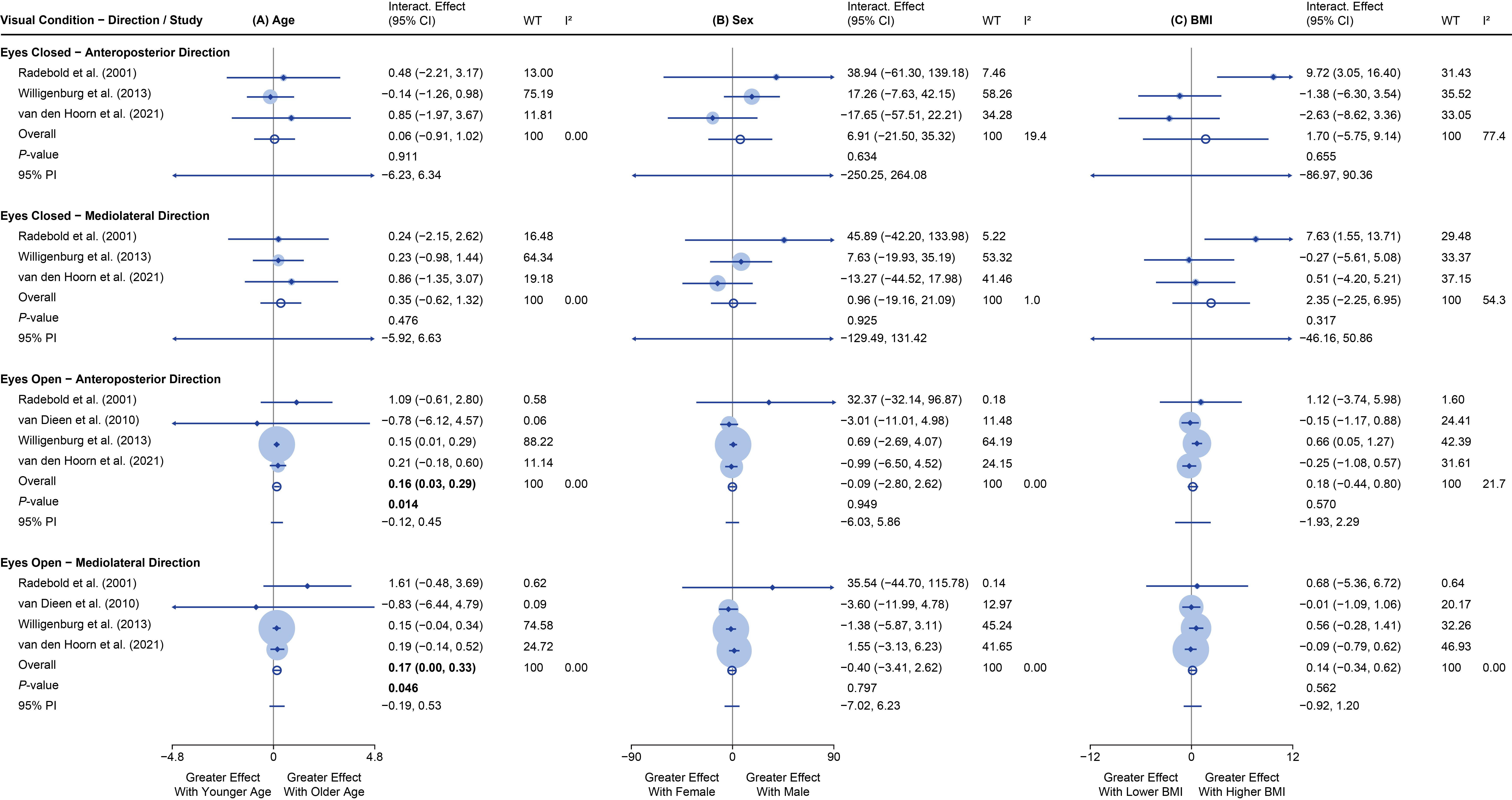

Supplement: S18 Fig — (A) age, (B) sex, and (C) body mass index (BMI). The results are presented as interaction (interact.) effect coefficients with 95% confidence intervals (95% CIs) using forest plots. Significant overall interact. effects with their respective P-values are highlighted in bold font. Greater effect in either directions indicates worse effect on trunk postural control for individuals with versus without low back pain. Sizing of circles reflects the weight (WT) of the contribution of a study on the pooled meta-analysis (weighted average) in percentage. I2 reflects the percentage of total variability due to heterogeneity between studies. 95% prediction interval (95% PI) reflects how much the effect size varies across studies. (JPG) [file pone.0296968.s053.jpg]

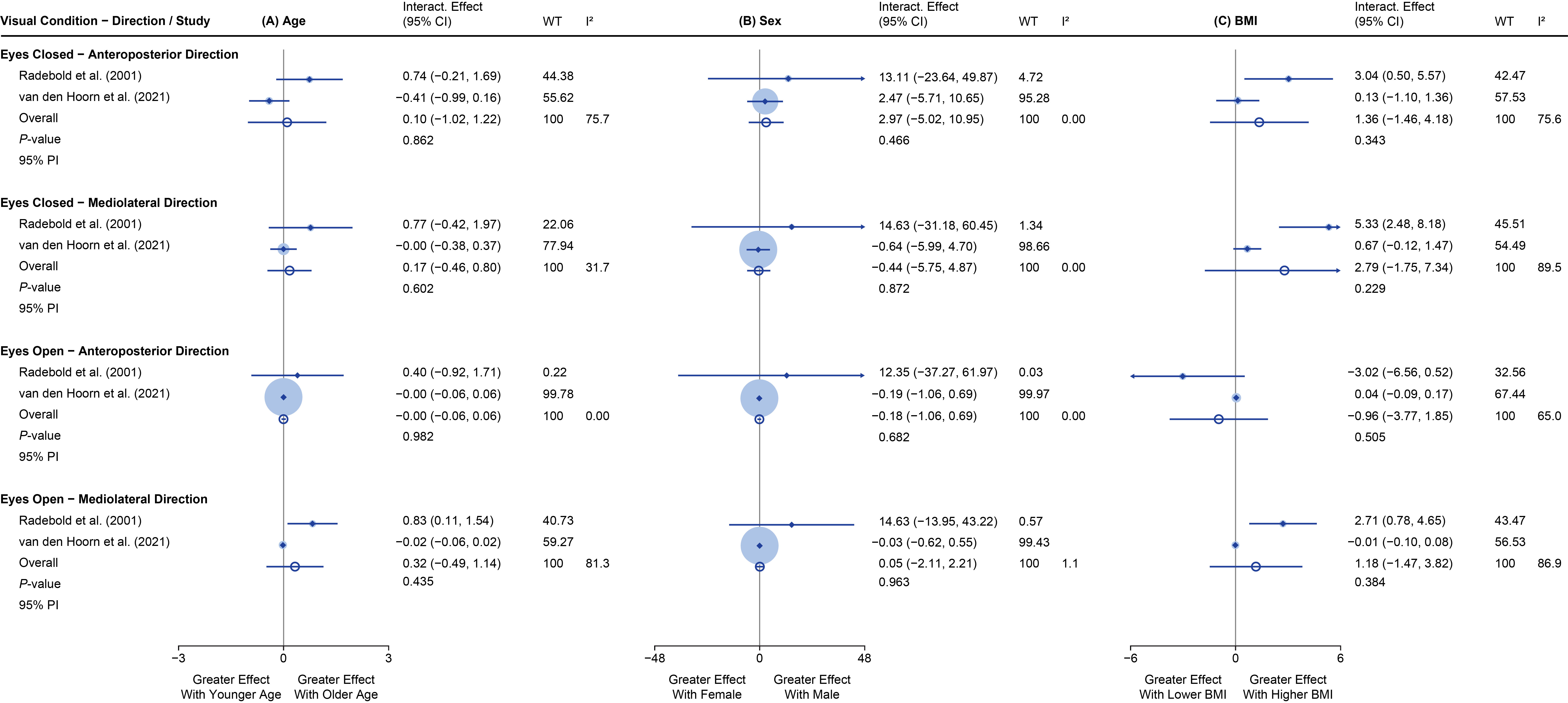

Supplement: S19 Fig — (A) age, (B) sex, and (C) body mass index (BMI). The results are presented as interaction (interact.) effect coefficients with 95% confidence intervals (95% CIs) using forest plots. Significant overall interact. effects with their respective P-values are highlighted in bold font. Greater effect in either directions indicates worse effect on trunk postural control for individuals with versus without low back pain. Sizing of circles reflects the weight (WT) of the contribution of a study on the pooled meta-analysis (weighted average) in percentage. I2 reflects the percentage of total variability due to heterogeneity between studies. 95% prediction interval (95% PI) reflects how much the effect size varies across studies. (JPG) [file pone.0296968.s054.jpg]

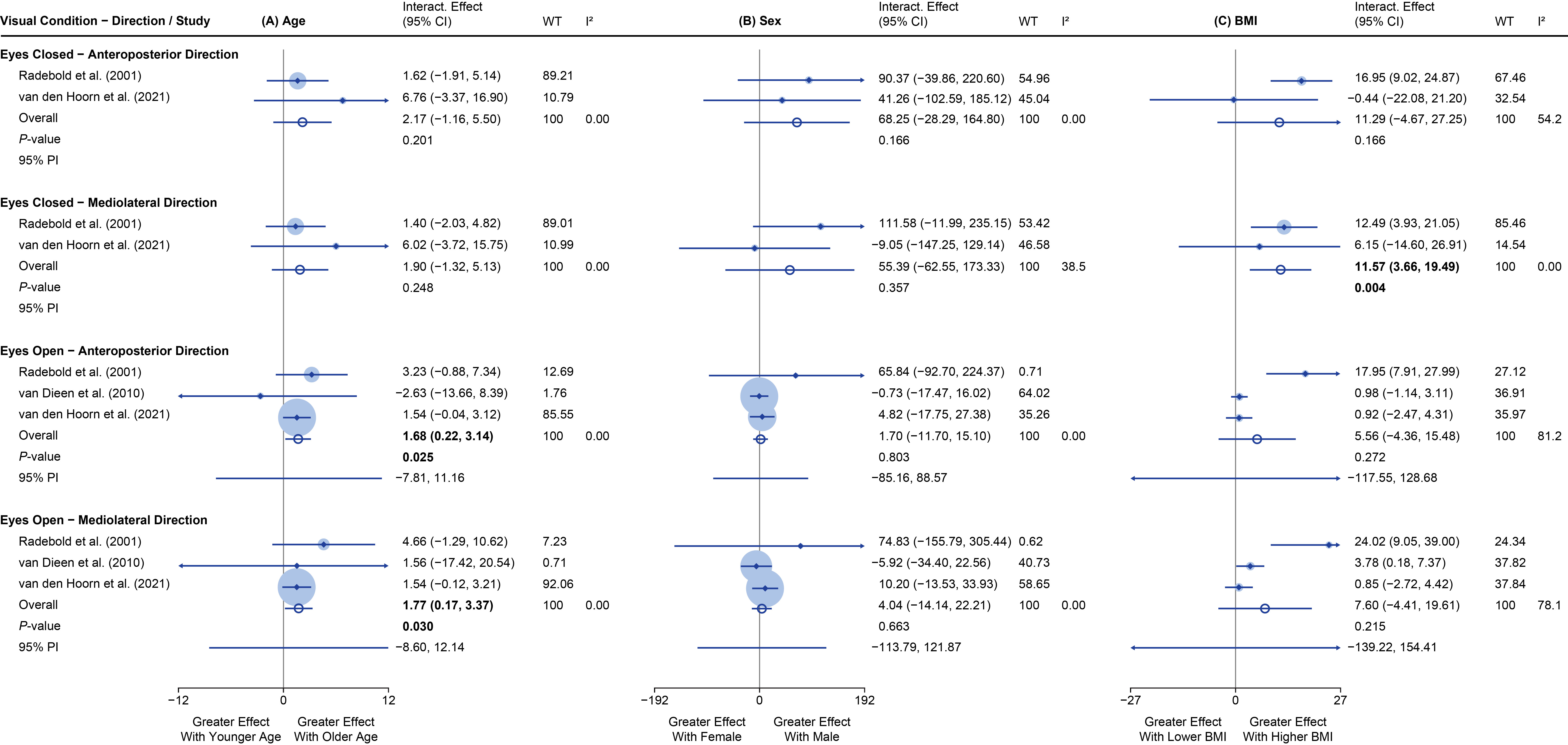

Supplement: S20 Fig — (A) age, (B) sex, and (C) body mass index (BMI). The results are presented as interaction (interact.) effect coefficients with 95% confidence intervals (95% CIs) using forest plots. Significant overall interact. effects with their respective P-values are highlighted in bold font. Greater effect in either directions indicates worse effect on trunk postural control for individuals with versus without low back pain. Sizing of circles reflects the weight (WT) of the contribution of a study on the pooled meta-analysis (weighted average) in percentage. I2 reflects the percentage of total variability due to heterogeneity between studies. 95% prediction interval (95% PI) reflects how much the effect size varies across studies. (JPG) [file pone.0296968.s055.jpg]

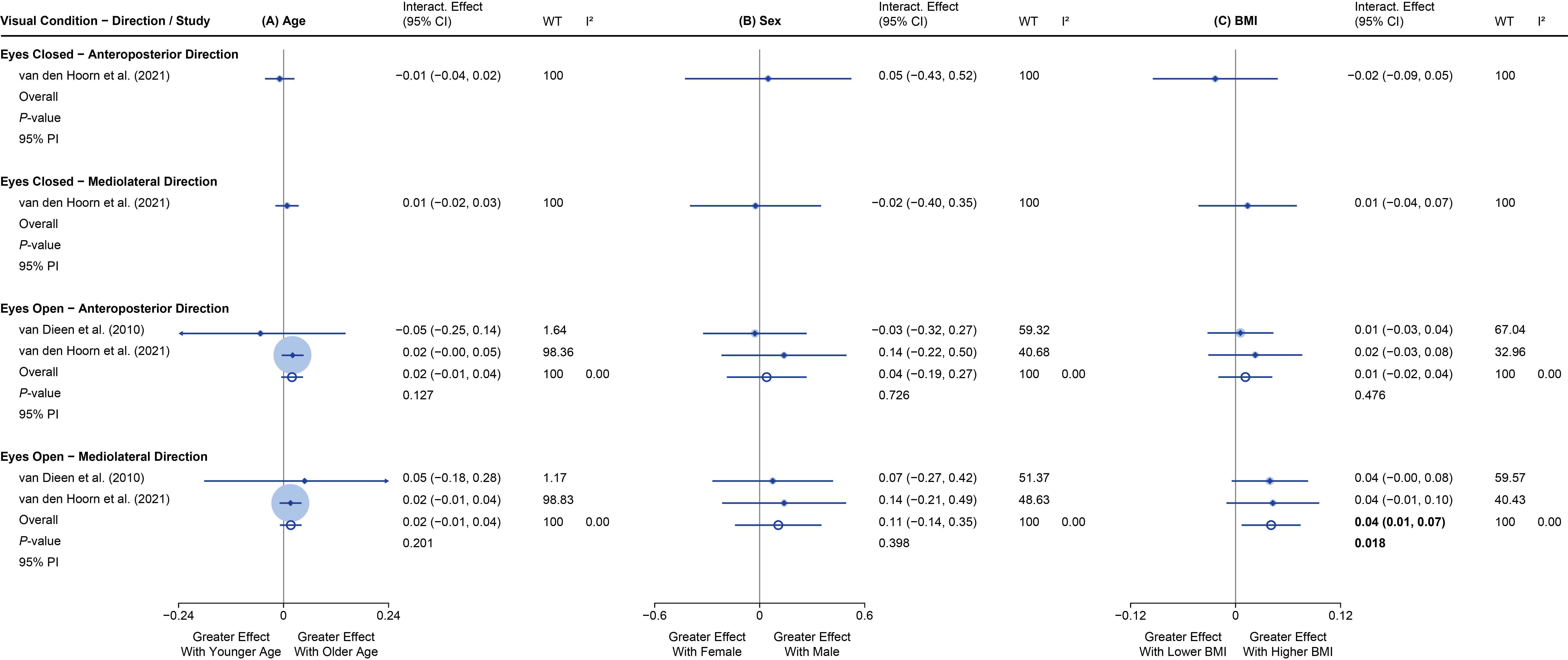

Supplement: S21 Fig — (A) age, (B) sex, and (C) body mass index (BMI). The results are presented as interaction (interact.) effect coefficients with 95% confidence intervals (95% CIs) using forest plots. Significant overall interact. effects with their respective P-values are highlighted in bold font. Greater effect in either directions indicates worse effect on trunk postural control for individuals with versus without low back pain. Sizing of circles reflects the weight (WT) of the contribution of a study on the pooled meta-analysis (weighted average) in percentage. I2 reflects the percentage of total variability due to heterogeneity between studies. 95% prediction interval (95% PI) reflects how much the effect size varies across studies. (JPG) [file pone.0296968.s056.jpg]

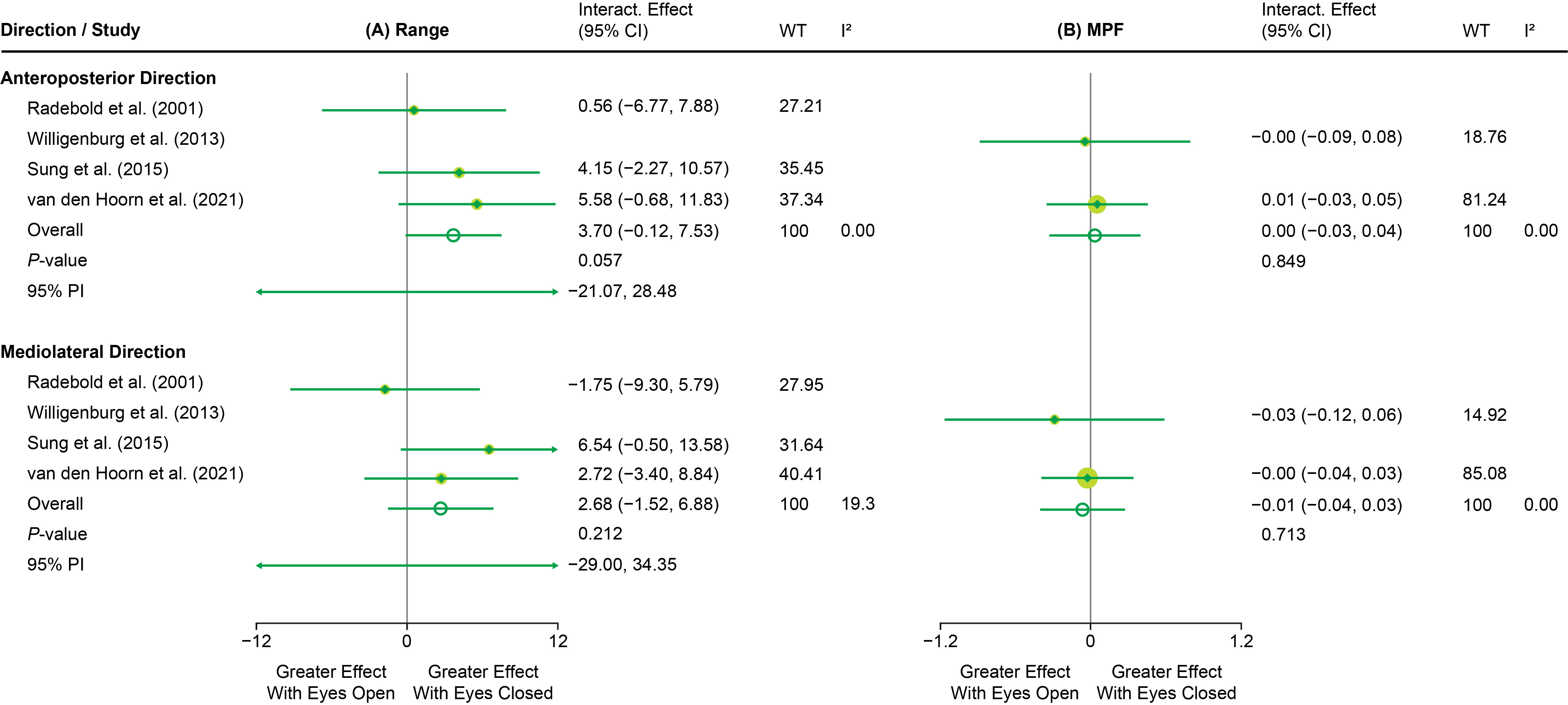

Supplement: S22 Fig — (A) range and (B) mean power frequency (MPF). The results are presented as interaction (interact.) effect coefficients with 95% confidence intervals (95% CIs) using forest plots. Significant overall interact. effects with their respective P-values are highlighted in bold font. Greater effect in either directions indicates worse effect on trunk postural control for individuals with versus without low back pain. Sizing of circles reflects the weight (WT) of the contribution of a study on the pooled meta-analysis (weighted average) in percentage. I2 reflects the percentage of total variability due to heterogeneity between studies. 95% prediction interval (95% PI) reflects how much the effect size varies across studies. (JPG) [file pone.0296968.s057.jpg]

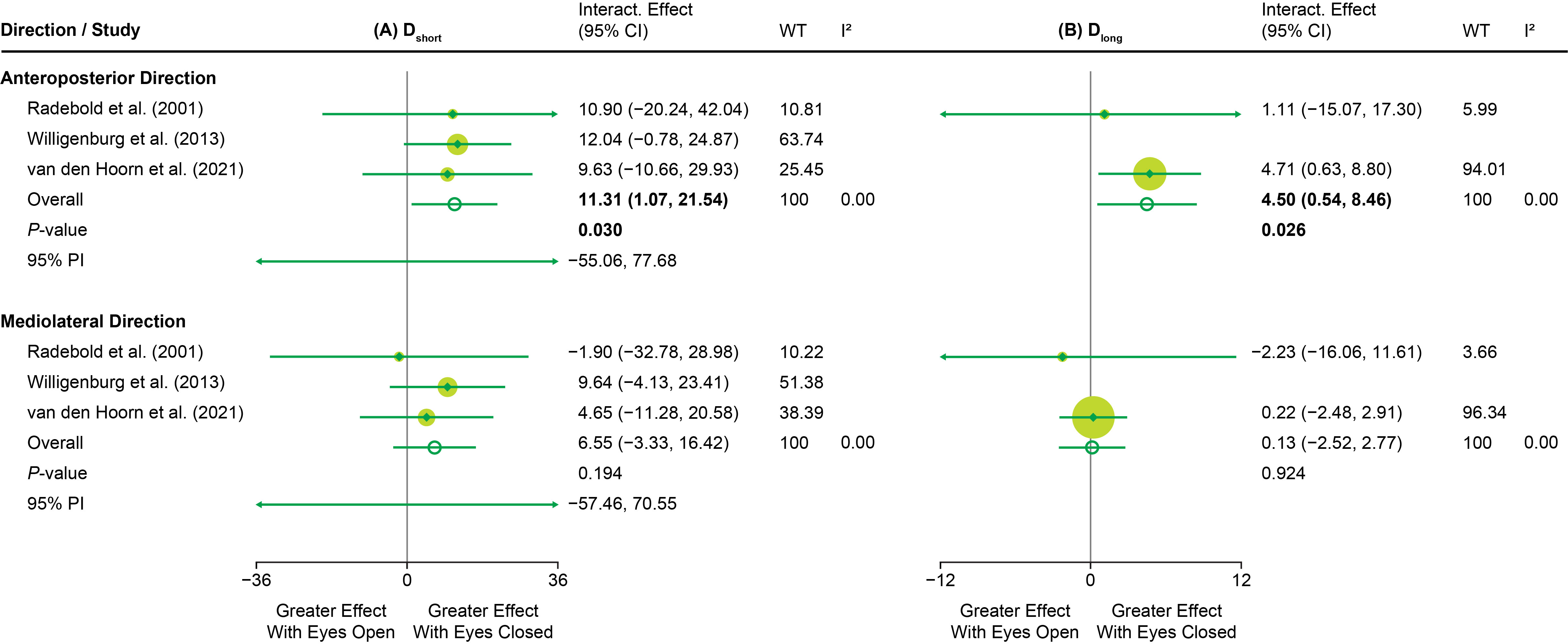

Supplement: S23 Fig — (A) short-term diffusion (Dshort) and (B) long-term diffusion (Dlong). The results are presented as interaction (interact.) effect coefficients with 95% confidence intervals (95% CIs) using forest plots. Significant overall interact. effects with their respective P-values are highlighted in bold font. Greater effect in either directions indicates worse effect on trunk postural control for individuals with versus without low back pain. Sizing of circles reflects the weight (WT) of the contribution of a study on the pooled meta-analysis (weighted average) in percentage. I2 reflects the percentage of total variability due to heterogeneity between studies. 95% prediction interval (95% PI) reflects how much the effect size varies across studies. (JPG) [file pone.0296968.s058.jpg]

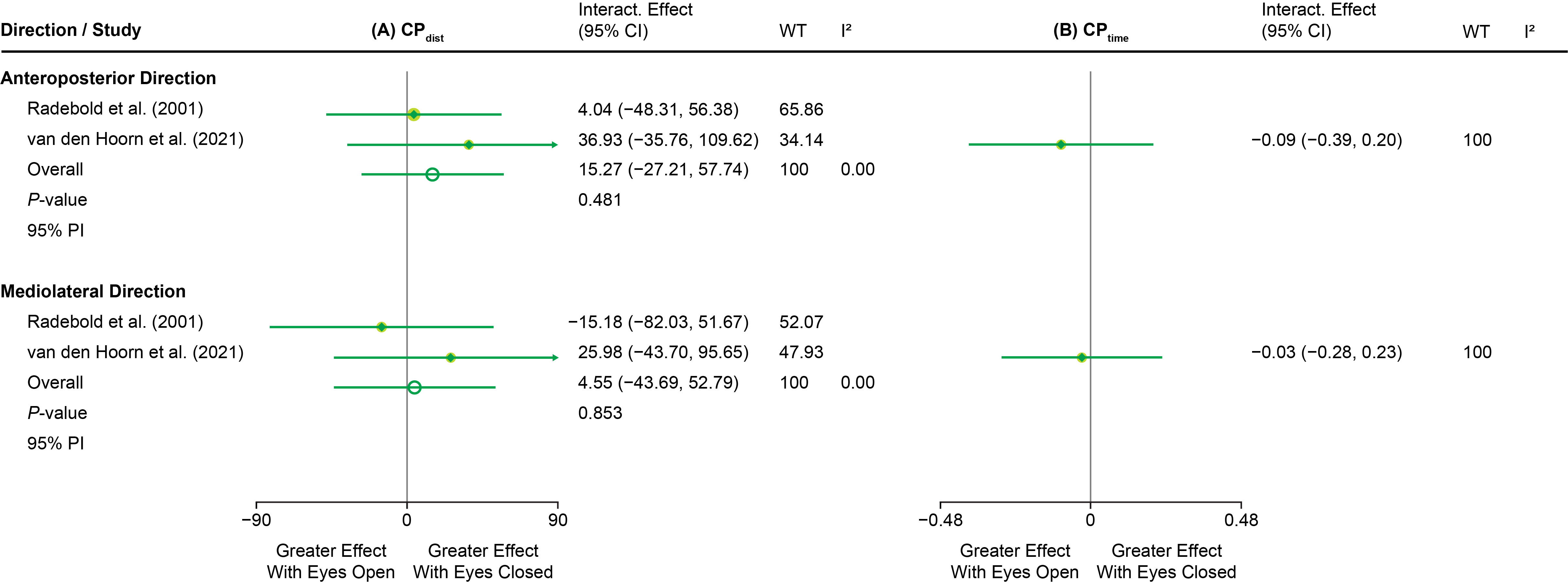

Supplement: S24 Fig — (A) mean squared distance coordinate of the critical point (CPdist) and (B) mean time coordinate of the critical point (CPtime). The results are presented as interaction (interact.) effect coefficients with 95% confidence intervals (95% CIs) using forest plots. Significant overall interact. effects with their respective P-values are highlighted in bold font. Greater effect in either directions indicates worse effect on trunk postural control for individuals with versus without low back pain. Sizing of circles reflects the weight (WT) of the contribution of a study on the pooled meta-analysis (weighted average) in percentage. I2 reflects the percentage of total variability due to heterogeneity between studies. 95% prediction interval (95% PI) reflects how much the effect size varies across studies. (JPG) [file pone.0296968.s059.jpg]
